# Novel Substrates for Kinases Involved in the Biosynthesis of Inositol Pyrophosphates and Their Enhancement of ATPase Activity of a Kinase

**DOI:** 10.3390/molecules26123601

**Published:** 2021-06-11

**Authors:** Raja Mohanrao, Ruth Manorama, Shubhra Ganguli, Mithun C. Madhusudhanan, Rashna Bhandari, Kana M. Sureshan

**Affiliations:** 1 School of Chemistry, Indian Institute of Science Education and Research Thiruvananthapuram, Kerala 695551, India; rajamohanrao1@gmail.com (R.M.); mithun716@iisertvm.ac.in (M.C.M.); 2 Laboratory of Cell Signalling, Centre for DNA Fingerprinting and Diagnostics, Hyderabad 500039, India; manorama@cdfd.org.in (R.M.); shubhraganguli@cdfd.org.in (S.G.); 3 Manipal Academy of Higher Education, Manipal 576104, India

**Keywords:** kinase, inositol pyrophosphate, IP6K1, VIP1 KD, ATPase

## Abstract

IP6K and PPIP5K are two kinases involved in the synthesis of inositol pyrophosphates. Synthetic analogs or mimics are necessary to understand the substrate specificity of these enzymes and to find molecules that can alter inositol pyrophosphate synthesis. In this context, we synthesized four *scyllo*-inositol polyphosphates—*scyllo*-IP_5_, *scyllo*-IP_6_, *scyllo*-IP_7_ and Bz-*scyllo*-IP_5_—from *myo*-inositol and studied their activity as substrates for mouse IP6K1 and the catalytic domain of VIP1, the budding yeast variant of PPIP5K. We incubated these *scyllo*-inositol polyphosphates with these kinases and ATP as the phosphate donor. We tracked enzyme activity by measuring the amount of radiolabeled *scyllo*-inositol pyrophosphate product formed and the amount of ATP consumed. All *scyllo*-inositol polyphosphates are substrates for both the kinases but they are weaker than the corresponding *myo*-inositol phosphate. Our study reveals the importance of axial-hydroxyl/phosphate for IP6K1 substrate recognition. We found that all these derivatives enhance the ATPase activity of VIP1. We found very weak ligand-induced ATPase activity for IP6K1. Benzoyl-*scyllo*-IP_5_ was the most potent ligand to induce IP6K1 ATPase activity despite being a weak substrate. This compound could have potential as a competitive inhibitor.

## 1. Introduction

**Figure 1:**
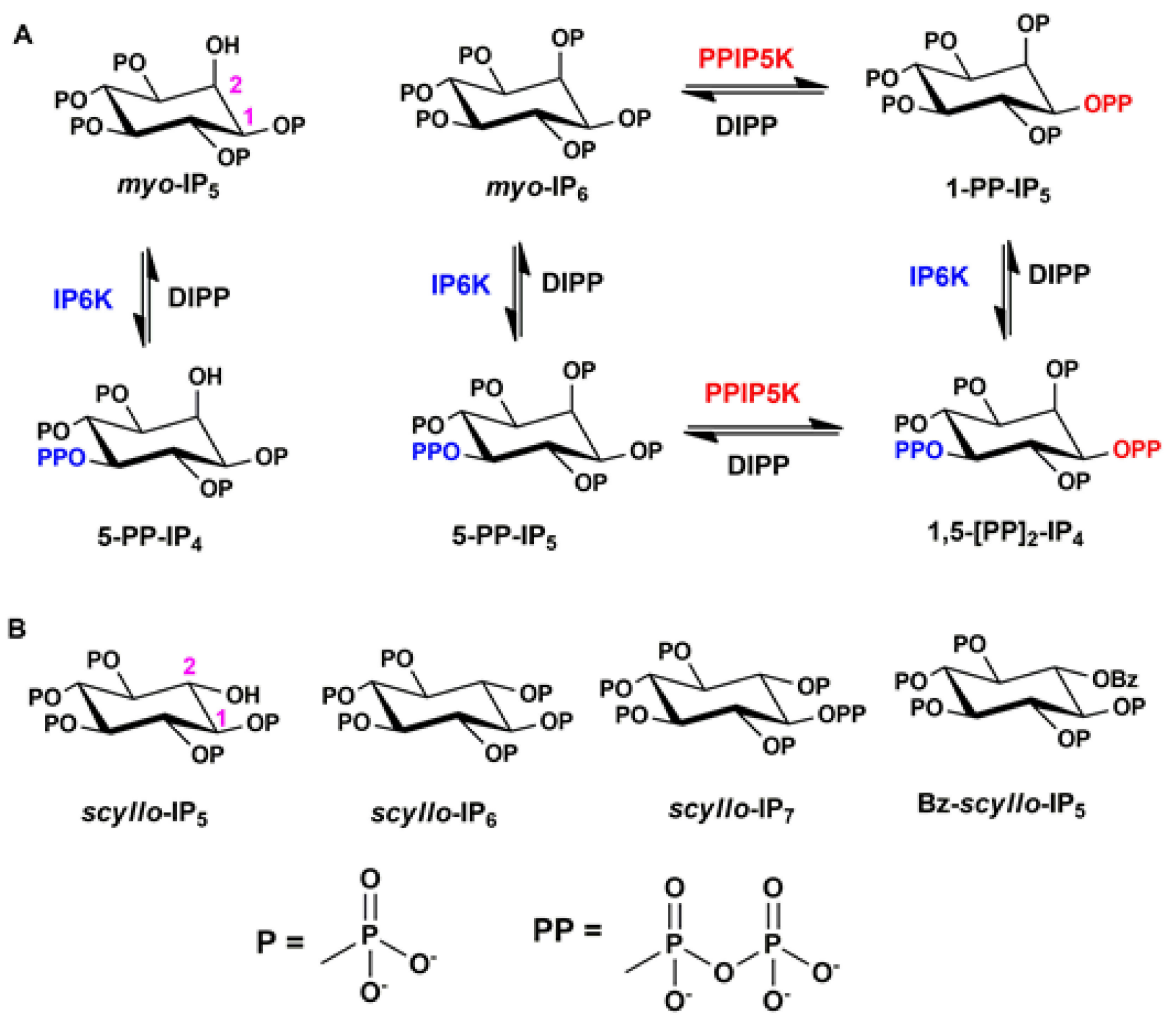
(**A**) Biosynthesis of inositol pyrophosphates in mammalian cells. (**B**) Proposed ligands to probe kinase activity.

Phosphoinositols play a variety of roles in cellular signaling [1,2]. Diphospho-inositol polyphosphates (PPIPns) are a class of highly phosphorylated *myo*-inositol derivatives that have one or two pyrophosphate moieties and occur only in eukaryotes [3,4,5]. PPIPns present in mammals include 5-PP-IP_4_, 5-PP-IP_5_, 1-PP-IP_5_ and 1,5-[PP]_2_-IP_4_, and their cellular levels are regulated by (i) IP6Ks, kinases which pyrophosphorylate the 5-position of *myo*-inositol, (ii) PPIP5Ks, kinases which pyrophosphorylate the 1-position and (iii) DIPPs, phosphatases which dephosphorylate the pyrophosphate to phosphate (Figure 1A) [6,7,8]. PPIPns play vital roles in apoptosis, cellular signaling, vesicular trafficking, metabolic homeostasis, cytoskeletal dynamics, exocytosis, insulin signaling, DNA damage repair, telomere maintenance, stress response, etc. [4,9,10,11,12,13,14,15,16,17,18,19,20,21,22,23,24]. Clear mechanisms of their wide-ranging functions are yet to be ascertained [23,25,26,27]. For biochemical and genetic exploration to unravel the structure-activity relationships (SAR) and mechanism of their actions, it is necessary to synthesize these molecules and their analogs [2,22,28,29,30]. Recently, Jessen et al. reported elegant approaches to synthesize various PPIPn analogs [31,32,33]. These analogs served as tools to unravel various biological roles played by inositol pyrophosphates [34]. Potter et al. [35] and Fiedler et al. [29,36,37] independently synthesized PPIPn analogs having non-hydrolyzable pyrophosphate surrogates and established, through biochemical assays, that this modification does not prevent target recognition and action of metabolizing enzymes. Both of these studies have also established that the recognizing enzymes can tolerate 2-*O*-substitution with a hydrophobic group. An extension of this study not only validated the previously reported [38] ligand induced ATPase activity of one of the kinases, PPIP5K2, but also unraveled the mechanism for the same [39]. Here we report the syntheses and biochemical studies of four novel substrate analogs of kinases involved in PPIPn biosynthesis, and ATPase activities of both these kinases.

As mentioned earlier, IP6Ks and PPIP5Ks are involved in the biosynthesis of PPIPns. IP_5_, IP_6_ and 1-PP-IP_5_ are the natural substrates for IP6Ks and get phosphorylated to 5-PP-IP_4_, 5-PP-IP_5_ and 1,5-[PP]_2_-IP_4_, respectively (Figure 1A). Similarly, IP_6_ and 5-PP-IP_5_ are the natural substrates for PPIP5Ks and get phosphorylated to 1-PP-IP_5_ and 1,5-[PP]_2_-IP_4_, respectively (Figure 1A) [40,41,42,43]. There is great interest in understanding the mechanism, function and substrate-specificity of these enzymes using various analogs as synthetic tools [30,35,36,39,44]. An important strategy to make analogs of IPns is to alter the inositol core. We chose *scyllo*-inositol, the C2-epimer of *myo*-inositol as the central core to synthesize PPIPn analogs. *Scyllo*-inositol phosphates constitute the second naturally abundant inositol phosphates [45,46,47] and many *scyllo*-inositol phosphates have been found in living organisms and in the environment [48,49]. Utilizing these *scyllo*-inositol phosphates as substrates for the PPIPn synthesizing kinases, would provide valuable information regarding enzyme function. In this context, we planned to synthesize *scyllo*-IP_5_, *scyllo*-IP_6_ and *scyllo*-IP_7_ (Figure 1B) to test them as substrates for Vip1 (budding yeast S. cerevisiae ortholog of PPIP5K) and mouse IP6K1. While the first two are mimics of IP_5_ and IP_6_ respectively, *scyllo*-IP_7_ is a mimic of both 1-PP-IP_5_ and 5-PP-IP_5_. We also planned to synthesize Bz-*scyllo*-IP_5_, as 2-*O*-benzyl/2-*O*-benzoyl substituted ligands are known to activate PPIP5K2 [39].

## 2. Results and Discussion

### 2.1. Synthesis of Scyllo-Inositol Phosphates

**Scheme 1:**
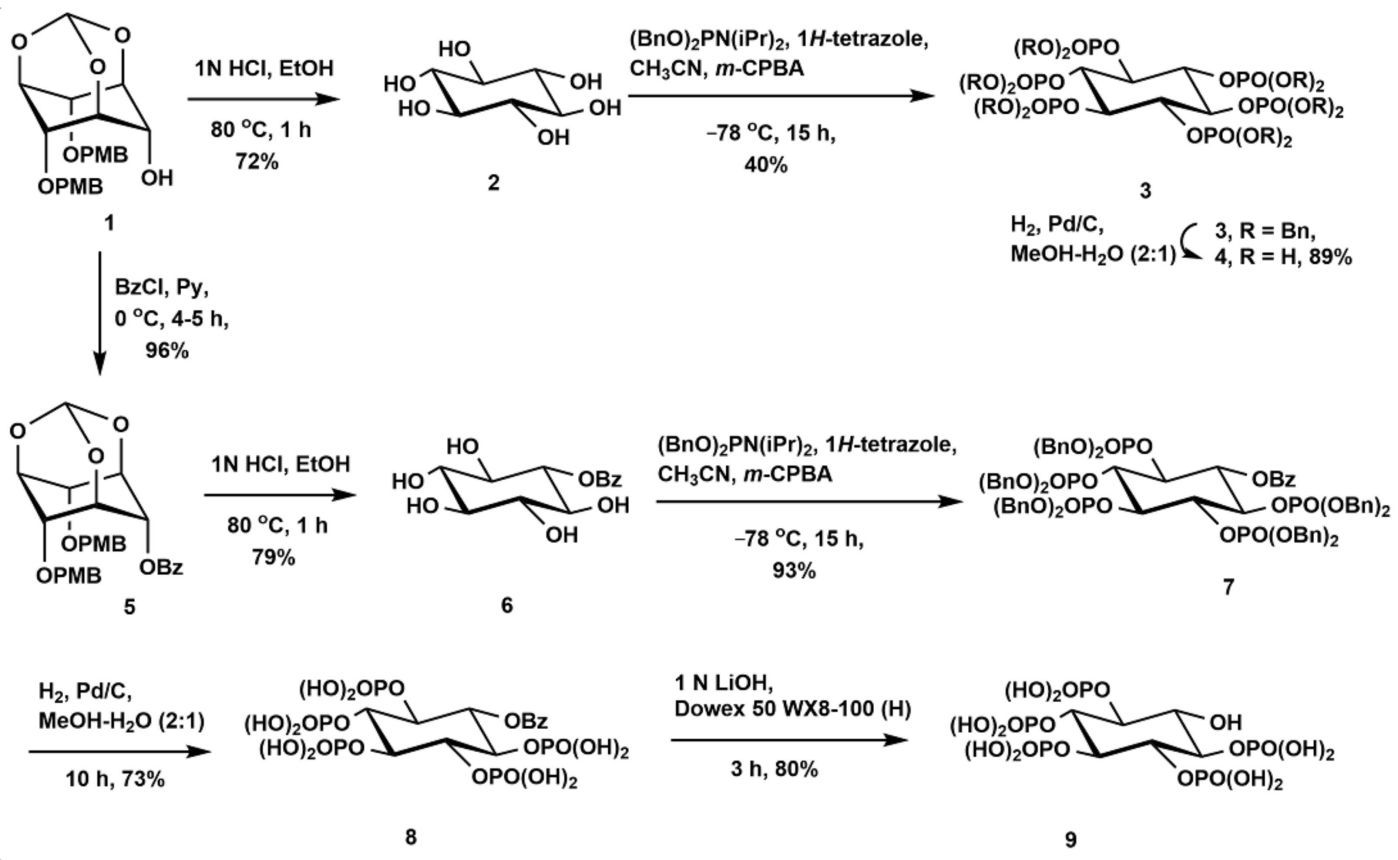
Synthesis of *scyllo*-IP_6_ (**4**), Bz-*scyllo*-IP_5_ (**8**) and *scyllo*-IP_5_ (**9**).

*Scyllo*-inositol derivative **1** was synthesized from *myo*-inositol in four steps by following the reported procedure [45]. Acidic hydrolysis of **1** with 1 N HCl in ethanol yielded *scyllo*-inositol (**2**), which, on phosphitylation with N, *N*-dibenzyl diisopropylphosphoramidite in the presence of 1*H*-tetrazole followed by oxidation with *m*-CPBA at −78 °C, gave hexaphosphorylated *scyllo*-inositol derivative **3** (Scheme 1). Hydrogenolysis of **3** yielded *scyllo*-IP_6_ (**4**). Benzoylation of **1** followed by acidic hydrolysis of the ester **5** gave benzoyl-*scyllo*-inositol (**6**). Phosphorylation of **6** resulted in the fully protected *scyllo*-inositol pentakisphosphate (**7**), which, on hydrogenolysis, yielded the benzoyl-*scyllo*-IP_5_ (**8**). Compound **8** on treatment with 1 N LiOH gave *scyllo*-IP_5_ (**9**), which was purified by passing through Dowex H^+^ resin.

**Scheme 2:**
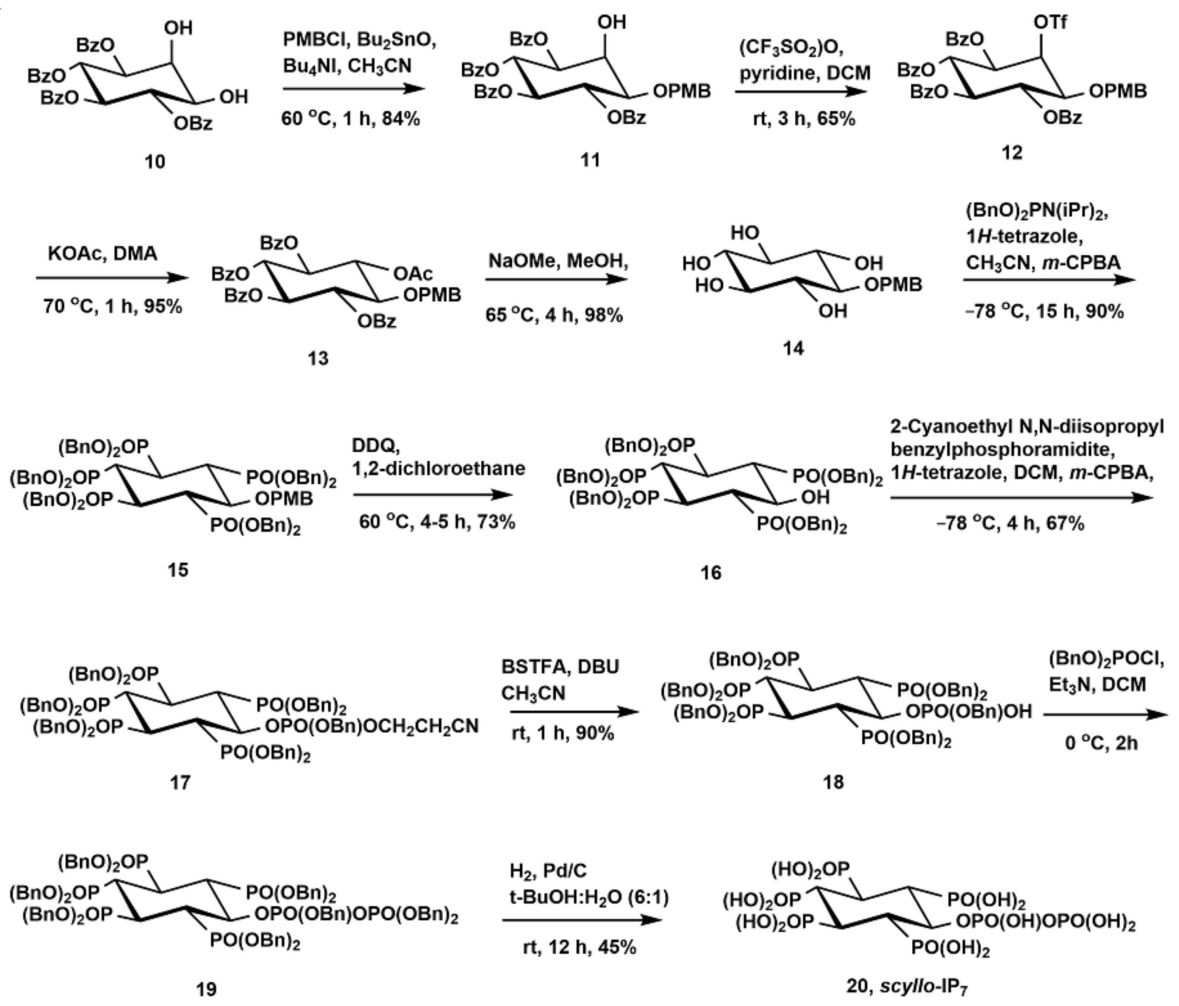
Synthesis of *scyllo*-IP_7_ (**20**).

**Scheme 3:**
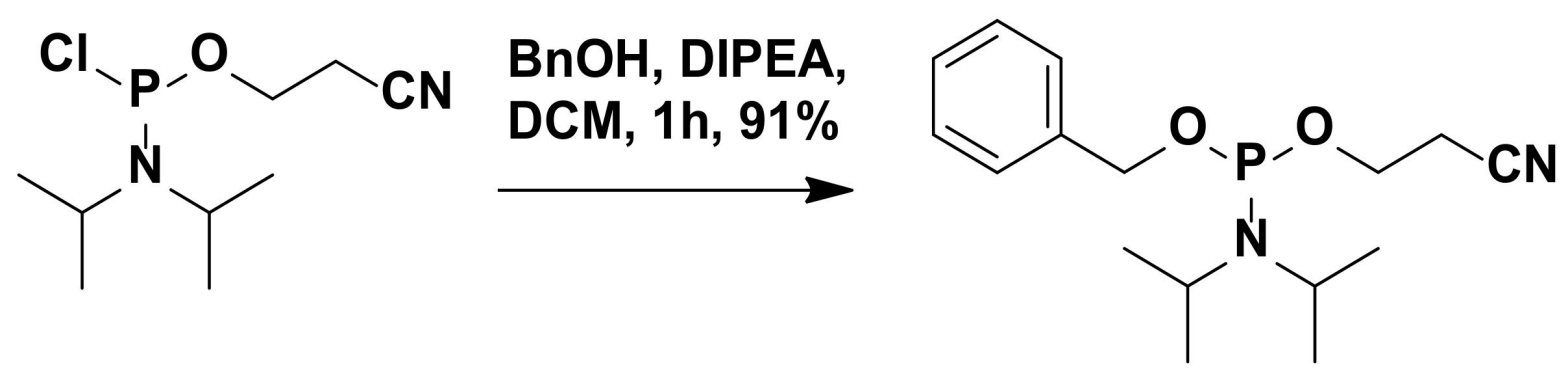
Synthesis of 2-Cyanoethyl-*N*,*N*-Diisopropylbenzylphosphoramidite.

Next, we turned our attention to synthesize *scyllo*-IP_7_ (Scheme 2). Attempts to remove the benzoyl group selectively from compound **7** failed. This prompted us to pursue another approach for the synthesis of *scyllo*-IP_7_ (**20**) starting from *myo*-inositol derivative **10** [50]. Selective *O*-alkylation of the equatorial hydroxyl group using *p*-methoxybenzyl chloride gave compound **11**, which, on triflylation, gave the triflate **12**. Nucleophilic displacement of the trifllate with acetate gave *scyllo*-inositol derivative **13**, which, on saponification, yielded the pentol **14**. Phosphitylation of pentol **14** followed by in situ oxidation resulted in the formation of fully protected pentakisphosphate **15**. Oxidative removal of the PMB group afforded compound **16**, whose structure was unambiguously confirmed by single crystal XRD analysis (see Figure 7 in Section 3.2.15., Crystal Data for **16**). Compound **16** was then phosphitylated with the unsymmetrical phosphoramidate, 2-cyanoethyl-*N*,*N*-diisopropyl benzylphosphoramidte (see Scheme 3 in Section 3.2.16.), followed by oxidation to get differently protected hexakisphosphate derivative **17**. Deprotection of the cyanoethyl group gave the phosphodiester **18**. Phosphorylation of **18** using dibenzylphosphoryl chloride [30,51] yielded fully protected *scyllo*-IP_7_ derivative **19**, which was used without purification for hydrogenolysis to get the *scyllo*-IP_7_ (**20**).

### 2.2. Analysis of Scyllo-Inositol Phosphates as Substrates for IP6K and PPIP5K

**Figure 2:**
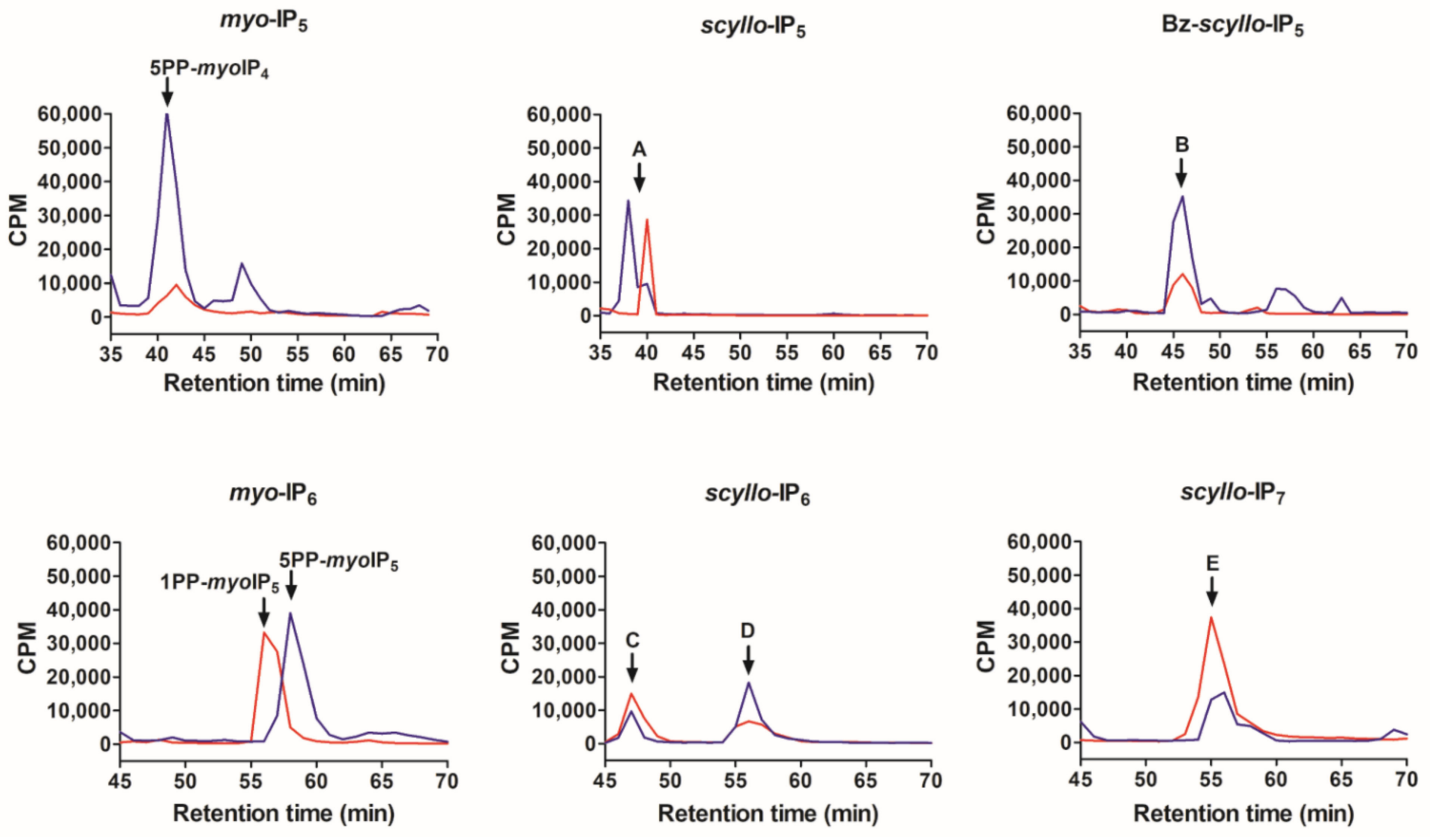
Chromatograms of the phosphorylated PPIPn products (indicated by arrows) formed by the catalysis of IP6K1 (blue) and VIP1-KD (red) on various IPn substrates. The IPn substrate used in each case is indicated above the chromatogram. The reaction products were resolved by strong anion exchange HPLC as described in Section 3.3. Retention times for standard *myo*-IPs were marked using radiolabeled inositol phosphates extracted from budding yeast (Section 3.3 and Supplementary Figure S2B). The traces represent one of four independent experiments. The well-characterized products of *myo*-IP_5_ and *myo*-IP_6_ are indicated. The PPIPn products of *scyllo*-IPs are likely to be as follows: A. PP-*scyllo*-IP_4_; B. PP-Bz-*scyllo*-IP_4_; C. PP-*scyllo*-IP_5_; D. [PP]_2_-*scyllo*-IP_4_/PPP-*scyllo*-IP_5_; E. [PP]_2_-*scyllo*-IP_4_/PPP-*scyllo*-IP_5_. The corresponding PPIPn products observed by PAGE analysis are similarly annotated in Figure S1. See Figure S2B,C for chromatograms of other *myo*-IPn and *myo*-PPIPn standards, and ATP, under the same conditions.

We investigated the ability of these *scyllo*-inositol phosphates to act as substrates for mouse IP6K1 (UniProt entry Q6PD10) and *S. cerevisiae* VIP1 (UniProt entry Q06685) in comparison with *myo*-IP_5_ and IP_6_. All these substrate analogs were incubated with ATP and purified *N*-terminally 6xHis-tagged mouse IP6K1 or the kinase domain (amino acid residues 1–535) of *S. cerevisiae* VIP1 fused *N*-terminally to GST (VIP1-KD). The IPn substrates and their PPIPn products were resolved by polyacrylamide gel electrophoresis (PAGE) and detected by staining with toluidine blue dye (Section 3.3). Upon the incubation of *myo*-IP_5_ or *myo*-IP_6_ with IP6K1 or VIP1-KD, we detected one or more *myo*-PPIPn products (Supplementary Figure S1). Our observations are in concordance with an earlier report demonstrating multiple PP-IP products arising from the action of these IP kinases on *myo*-IP_5_ or *myo*-IP_6_, many of which are uncharacterized [52]. All *scyllo*-IPns served as substrates for both IP6K1 and VIP1-KD (Supplementary Figure S1). PAGE analysis of PP-IPs is not suitable for the quantitative comparison of *scyllo*- vs. *myo*-IPs as substrates, owing to the variation in staining of different IPns by toluidine blue dye. Therefore, to quantify the PPIPn products arising from *myo*- and *scyllo*-IPn substrates, we resorted to the use of radiolabeled ATP. Equimolar quantities of the different IPn substrates were incubated with either enzyme in the presence of [γ-^32^P]ATP; the enzymatic reactions were quenched by adding perchloric acid and then neutralized with potassium carbonate and EDTA (Section 3.3). The products were separated by strong anion exchange HPLC, and 1 mL fractions were collected and counted using a liquid scintillation counter. The substrates and the corresponding chromatograms showing the peaks due to phosphorylated products are shown in Figure 2. Each HPLC peak represents the addition of a radiolabeled phosphate onto the IPn substrate. Owing to the inability to use an ATP regeneration system along with radiolabeled ATP (see Section 3.3), in many cases, we observed fewer products by HPLC compared with PAGE analysis.

We have proposed probable structures for the *scyllo*-PPIPn products seen by gel electrophoresis and HPLC (see legends for Figure 2 and Supplementary Figure S1). Our proposed structures are based on the known catalytic properties of IP6K1 and VIP1 towards *myo*-inositol substrates. IP6K1 catalyzes the addition of a β-phosphate onto an α- phosphate, and a γ-phosphate onto a β-phosphate on C5 of *myo*-IP_5_ and *myo*-IP_6_ [19,53], whereas VIP1 acts to add a β-phosphate on C1 of *myo*-IP_6_ and *myo*-IP_7_ [13,17]. As all equatorial carbon atoms of *scyllo*-inositol are indistinguishable from each other, we propose that the same *scyllo*-PPIPn products arise by the action of both enzymes. Therefore, IP6K1 and VIP1 probably act on *scyllo*-IP_5_, Bz-*scyllo*-IP_5_ and *scyllo*-IP_6_ to yield PP-*scyllo*-IP_4_, PP-Bz-*scyllo*-IP_4_ and PP-*scyllo*-IP_5_ (i.e., *scyllo*-IP_7_), respectively (peaks A, B and C in Figure 2). The product formed by the addition of two phosphates onto *scyllo*-IP_6_ (peak D), and one phosphate onto *scyllo*-IP_7_ (peak E), are likely to correspond to a *scyllo*-inositol with 8 phosphate groups. This could be [PP]_2_-*scyllo*-IP_4_ or PPP-*scyllo*-IP_5_. The only unambiguous means of identifying the structures of any of the *scyllo*-PPIPn products is via NMR. However, it is not trivial to obtain sufficient quantities of purified, enzymatically synthesized PPIPn products required for such an analysis. The masses of the products we have proposed are based on the bands visualized by PAGE (separated by a single phosphate units), and the resolution of ^32^P-labeled inositol phosphates by HPLC. Despite repeated attempts we were unable to detect enzymatically synthesized *scyllo*-PPIPn products by mass spectrometry—HPLC-purified radiolabeled products cannot be loaded onto a mass spectrometer due to radiation safety protocols, and contaminating substances from polyacrylamide gels interfered with our attempts to analyze gel-eluted *scyllo*-IPs. For the above reasons, the proposed structures of the *scyllo*-PPIPn products detected by PAGE and HPLC analysis are likely, but not certain.

**Figure 3:**
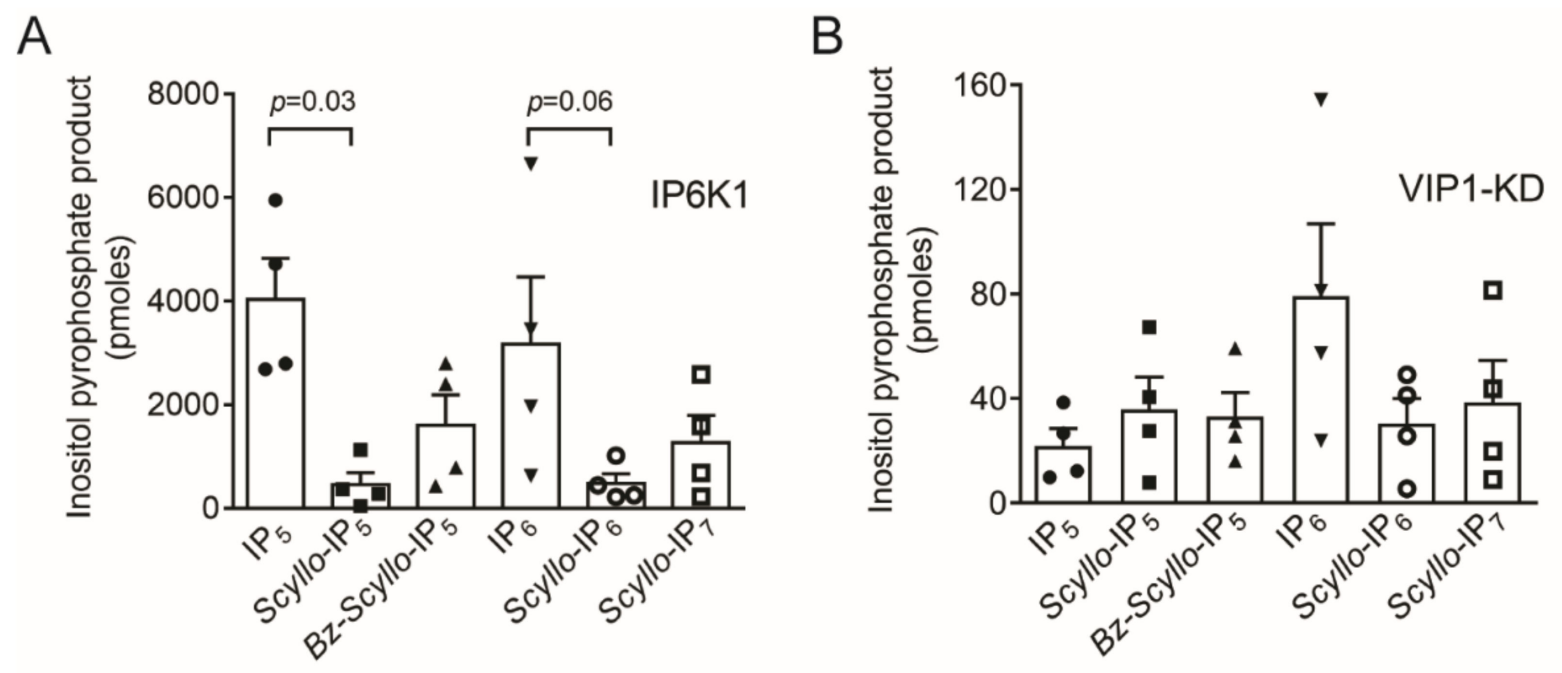
Extent of conversion of the substrates to products by (**A**) IP6K1 in presence of ATP (1 mM) and IPn (200 µM). (**B**) VIP1-KD in presence of ATP (40 µM) and IPn (100 µM). PPIPn product formation was calculated as described in Section 3.3. The graphs show individual data points and mean ± SEM from four independent experiments. Data was analyzed using a two-tailed Mann–Whitney test to compare the PPIPn product of *myo*-IPn with its corresponding *scyllo*-derivative. *p* values are indicated where significant.

Analysis of the conversion of the substrates to products indicates that all *scyllo*-IPns tested are substrates for IP6K1, but they are relatively poorer substrates than the corresponding *myo*-inositol phosphates (Figure 3A). For IP6K1, *myo*-IP_5_ shows the best conversion rate and is approximately 1.5-fold the conversion rate of *myo*-IP_6_. This is in agreement with the trend reported previously [53]. Interestingly, *scyllo*-IP_5_ and *scyllo*-IP_6_ are poor substrates for IP6K1 compared with their *myo*-counterparts. This suggests that an axial C2-hydroxyl/phosphate has an important role in active-site binding. This requirement was however partially offset by the presence of a benzoyl group on *scyllo*-IP_5_. It has been shown that IP6K1 can act on 5PP-*myo*-IP_5_ to generate a triphosphate containing species— 5PPP-*myo*-IP_5_ [19]. We observed that IP6K1 can also act on *scyllo*-IP_7_ to generate a product (Figure 2, peak E), which possibly contains a triphosphate moiety.

In contrast to IP6K1, all *scyllo*-IPns acted as moderate substrates for VIP1 (Figure 3B). *myo*-IP_5_ is the poorest substrate of all those tested. This is in agreement with the fact that 1-PP-IP_4_ has not been found in vivo and also with a previous report that *myo*-IP_5_ is not a substrate for PPIP5Ks, mammalian orthologs of Vip1 [17]. *myo*-IP_6_ is the best substrate; the approximately 3-fold difference between *myo*-IP_5_ and *myo*-IP_6_ phosphorylation by VIP1-KD suggests that the 2-phopshate is crucial for active site binding/effective phosphotransfer. All *scyllo*-IPns tested were weaker substrates for VIP1 compared with *myo*-IP_6_, although the difference was not statistically significant. This suggests that the loss of the axial C2-phosphate can be partially compensated by an equatorial phosphate at that position. Interestingly, the presence of a benzoyl group on *scyllo*-IP_5_ did not interfere with its ability to serve as a substrate for VIP1. It is to be noted that structurally similar 2-*O*-benzyl-*myo*-IP_5_ was reported to be an inhibitor of PPIP5K but an activator of its ATPase activity [39].

**Figure 4:**
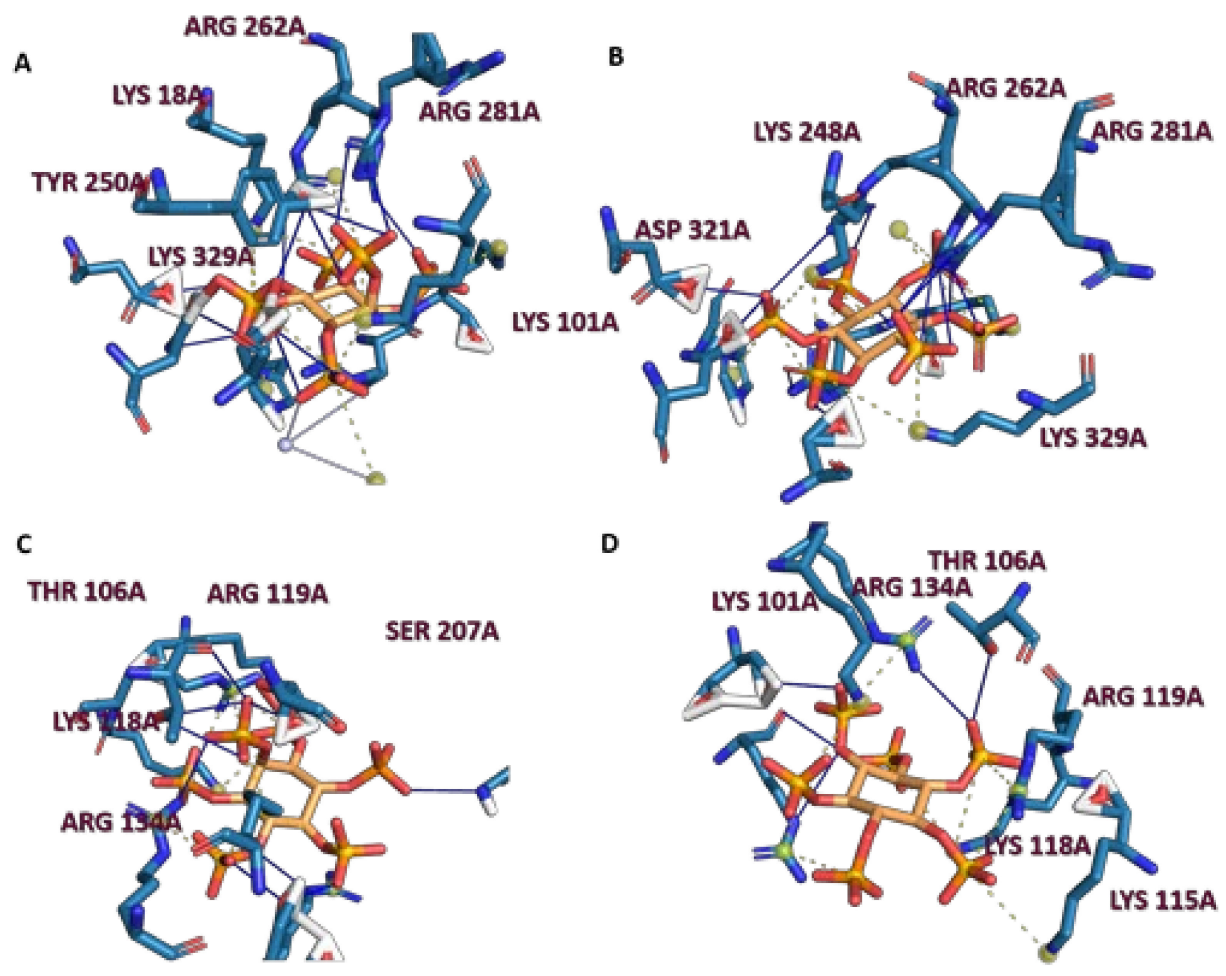
Molecular Docking results of *myo* and *scyllo*-IP_6_ on PPIP5K2 and *Eh*IP6KA (GOLDScore); (**A**) *myo*-IP_6_ at the active site of PPIP5K2; (**B**) *scyllo*-IP_6_ at the active site of PPIP5K2; (**C**) *myo*-IP_6_ at the active site of *Eh*IP6KA; (**D**) *scyllo*-IP_6_ at the active site of *Eh*IP6KA. All interactions were visualized using PLIP while further image processing was carried out using PyMOL [55].

In order to get further insights about the IPn-kinase interactions, we conducted molecular docking with the kinases and *myo*-IP_6_ and *scyllo*-IP_6_ as ligands using CCDC GOLD suit (see Supplementary Materials and Supplementary Figure S3). Since exact structures for ScVIP1 and IP6K1 are not available, the molecular docking experiments were carried out at the active sites of analogous hPPIP5K2 and IP6KA kinase of *Entamoeba histolytica* (PDB code: 3T9C, 4O4F) [17,54]. With both the enzymes, *myo*-IP_6_ showed better fitness score than *scyllo*-IP_6_ and this is in agreement with the experimental observation of higher turnover for the *myo*-IPns. Furthermore, total number of attractive interactions between the enzyme and ligands was more for *myo*-IP_6_ in both the cases. While *myo*-IP_6_ had 21 interactions with IP6K, the *scyllo*-isomer had only 17 interactions. Similarly, PPIP5K2 kinase domain had 28 interactions with the *myo*-IP_6_ and 26 interactions with the *scylllo*-IP_6_. Interestingly, the axial C2 phosphate in *myo*-IP_6_ shows seven interactions with the IP6K side-chains (K101, R119, S207, T106, R134), but the corresponding equatorial phosphate in *scyllo*-IP_6_ shows four interactions (R119, K118, R134, T106). In the case of PPIP5K2, the axial phosphate at C2 position shows seven interactions (K329, R262, K248, R281, Y250) and equatorial phosphate makes five interactions (K329, K248, R262, R281) (Figure 4). This difference in number and strength of such interactions can influence the substrate binding and, thus, the kinase activity.

The non-productive activation of PPIP5K2 by inositol phosphates leading to the hydrolysis of ATP to ADP (ATPase activity) has been reported [38]. Later, biochemical studies and mutagenesis studies, using several synthetic analogs, revealed the existence of a surface-exposed second binding site within the kinase domain of PPIP5K2, which acts as a capture site for the substrate, and that the natural substrate occupies this site only transiently before being delivered to the catalytic site [39]. The ATPase activity was found to be associated with ligand occupancy at the capture site. This study also found that 2-*O*-benzyl-*myo*-IP_5_ interacts weakly with the catalytic site but binds better at the capture site of PPIP5K2, and consequently it is not a substrate of this kinase but a ligand that induces its ATPase activity [39]. We were curious to investigate the ability of *scyllo*-inositol phosphates to induce the ATPase activity of VIP1. A recent report describing a high-throughput assay for IP6K1 activity suggested that measuring ADP production from ATP is an easier alternative to HPLC-based measurement of 5-PP-IP_5_ produced from IP_6_ [56]. By extension, PPIPn products of *scyllo*-IPs should also lead to concomitant ADP release from ATP, which can be used to measure kinase activity against these substrates. Furthermore, many kinases are known to possess inherent ATPase activity [57,58,59,60,61,62,63], and PPIP5K2 shows substrate-stimulated ATP hydrolysis (38, 39). With these considerations, we studied ADP production by both kinases, IP6K1 and VIP1-KD. We ruled out the presence of any contaminating ATPase or phosphatase in the preparation of 6xHis-mIP6K1 or GST-VIP1-KD purified from the *E. coli* expression system (see Section 3.4 and Supplementary Figure S4).

**Figure 5:**
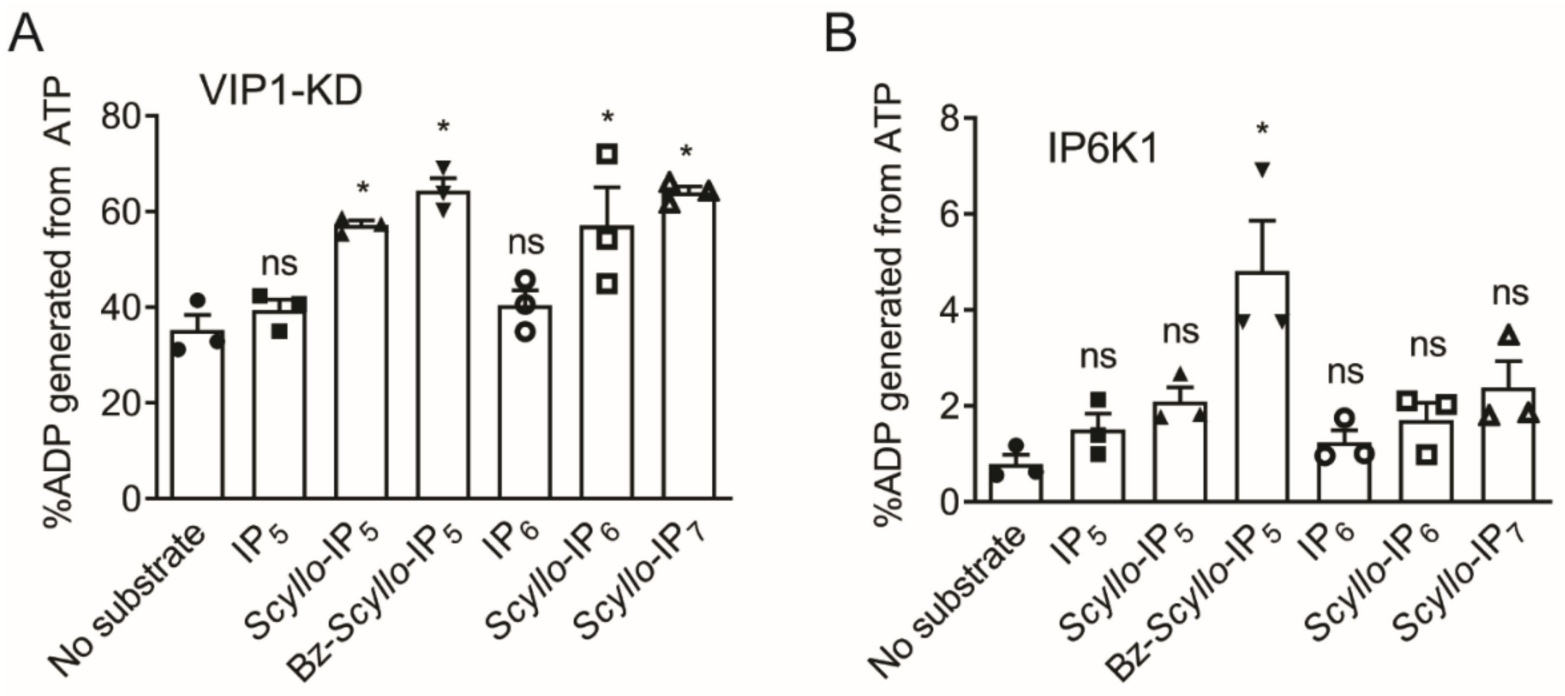
The effects of the *myo*- and *scyllo*-inositol phosphates upon ATPase activity of (**A**) VIP1-KD in presence of ATP (40 µM) and IPn (100 µM). (**B**) IP6K1 in presence of ATP (1 mM) and IPn (200 µM). The graphs show individual data points and mean ± SEM from three independent experiments. Data was analyzed using one-way ANOVA to compare each condition with ATPase activity in the absence of substrate. * *p* ≤ 0.05; ns, not significant, *p* > 0.05.

**Figure 6:**
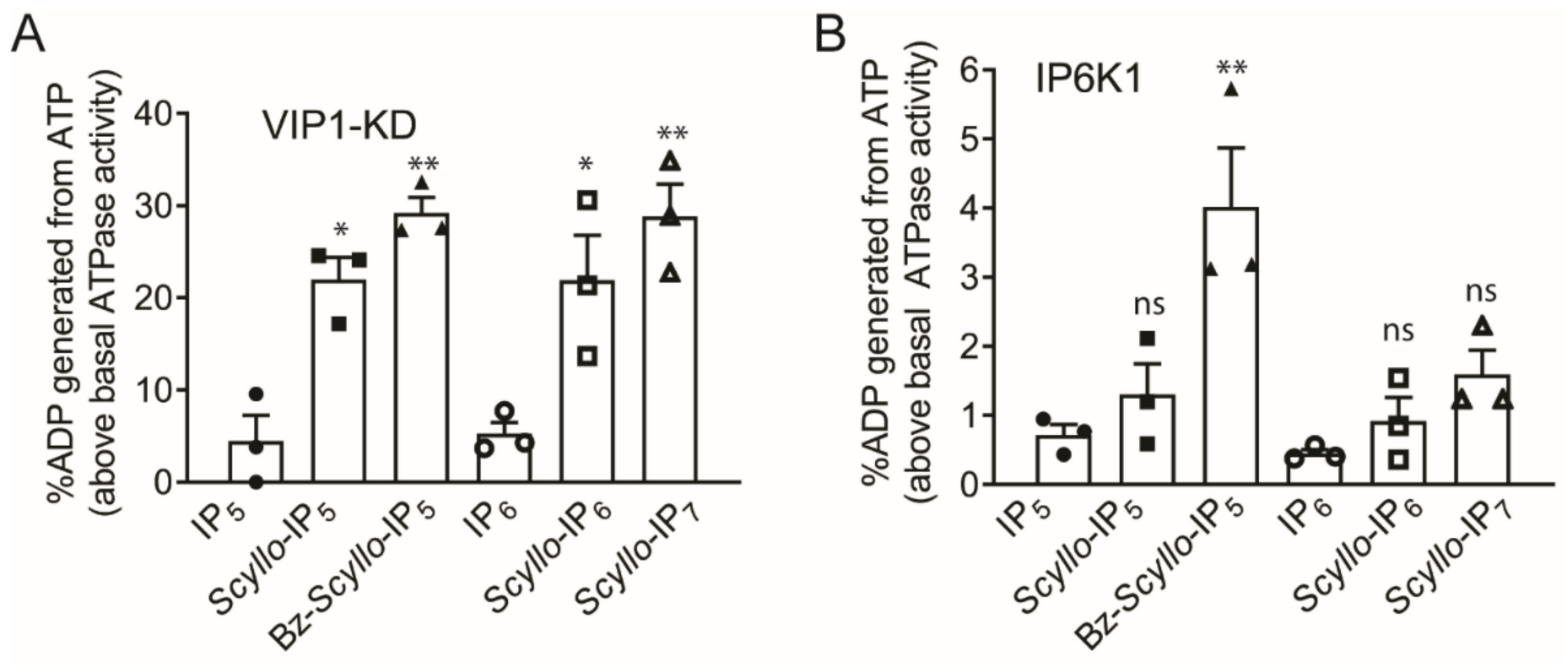
Percentage of ADP generated in the kinase reactions for (**A**) VIP1-KD and (**B**) IP6K1, after subtraction of basal ADP generated in the absence of substrate, for the data shown in Figure 5. The graphs show individual data points and mean ± SEM from three independent experiments. Data was analyzed using one-way ANOVA to compare ATPase activity in the presence of each *scyllo*-IPn with ATPase activity in the presence of either *myo*-IP_5_ or *myo*-IP_6_ (which were not significantly different from each other). ** *p* ≤ 0.01; * *p* ≤ 0.05; ns, not significant, *p* > 0.05.

We used the ADP-GloTM kinase assay kit to monitor the conversion of ATP to ADP by IP6K1 and VIP1-KD in the presence of their IP substrates (Section 3.4) [56]. ADP-GloTM kinase assay is a luminescence detection assay method to measure the percentage of ATP that is converted to ADP during a kinase reaction. VIP1-KD showed considerable basal activity, converting close to 40% of the ATP supplied during the reaction to ADP (Figure 5A), which is in agreement with its known inherent ATPase activity [38]. In contrast to the stimulation of non-productive ATPase activity by IP_5_ and IP_6_ reported in the case of PPIP5K2 [38,39], in our hands, both IP_5_ and IP_6_ showed only negligible ATPase activation of VIP1 over the basal level (Figure 6A). This may be due to differences in the behavior of VIP1 and PPIP5K2. It may also be a reflection of the significantly lower levels of ATP used in our assay—at high concentrations of ATP (5–10 mM), the presence of IP_5_ and IP_6_ leads to a 2- to 4-fold elevation in the release of Pi from ATP by PPIP5K2 (38). Under physiological ATP concentrations, IP_5_ or IP_6_ may stimulate ATPase activity of the VIP1-KD; substrate-independent ATPase activity of VIP1-KD may also increase at physiological [ATP]. The 4–6% of ATP consumed in the presence of IP_6_ (Figure 6A) is sufficient for the conversion of approximately 2% of IP_6_ to 1-PP-IP_5_, in agreement with the extent of product formation shown in Figure 3B. All the *scyllo*-IPns tested showed up to two-fold enhancement in ATPase activity of VIP1 (Figure 5A). Interestingly, *scyllo*-IP_5_ and *scyllo*-IP_6_ enhance ATPase activity four times more than their corresponding *myo*-IPns (Figure 6A). This property of *scyllo*-IP_5_ and *scyllo*-IP_6_ to induce non-productive ATPase activity while being moderate substrates, suggests that these ligands may bind better at the capture site than the catalytic site of VIP1. Bz-*scyllo*-IP_5_ and *scyllo*-IP_7_ are also potent inducers of VIP1 ATPase activity; while the ATPase activity of the former is in agreement with the previously reported effect of 2-*O*-benzyl substituted *myo*-IP_5_ enhancing non-productive ATPase activity of PPIP5K2 [39], in case of the latter, the absence of an axial-phosphate group may weaken its interaction with the active site and thus increase its residence time at the capture site, stimulating futile ATPase activity.

IP6K1 showed a very low basal activity, suggesting that it is not an inherent ATPase (Figure 5B). The *myo*- and *scyllo*-inositol phosphates increased ADP production marginally (Figure 6B), in keeping with the transfer of the gamma phosphate from ATP to generate the corresponding inositol pyrophosphate (Figure 3A). An intriguing exception to this was Bz-*scyllo*-IP_5_, which yielded a low amount of inositol pyrophosphate product, but significantly stimulated ATPase activity in IP6K1. Further studies will be required to establish non-productive ATPase activity in IP6K1.

In conclusion, we synthesized a series of *scyllo*-inositol phosphates as substrates for the two kinases involved in diphosphoinositol polyphosphate synthesis. This study revealed the tolerance of these enzymes for the *myo*- to *scyllo*- isomers. The relative potentials of these compounds as substrates in comparison with natural *myo*-inositol based substrates gave details and structural factors influencing binding of substrate to the enzymes. This study established that the absence of an axial hydroxyl/phosphate at the C2 position of an inositol phosphate reduces its potential as a substrate for IP6K1. In contrast, VIP1 appeared to accommodate the absence of an axial-2-phosphate more easily compared with IP6K1. All the *scyllo*-inositol derivatives tested are efficient in enhancing the ATPase activity of VIP1, which may be due to the preference of these compounds for the capture site on VIP1. Design of novel inhibitors based on these SAR studies and studies to identify the structures and biological effects of the products of these unnatural substrates are underway.

## 3. Materials and Methods

### 3.1. General

All chemicals and solvents were purchased from commercial suppliers and used directly. Solvents were dried using standard laboratory procedures and stored under anhydrous conditions. Reactions were monitored by thin layer chromatography (TLC) using Merck pre-coated silica plates (60 F254) (Darmstadt, Germany). TLC plates were visualized under ultraviolet light at 254 nm and also by charring using cerium ammonium molybdate solution (235 mL of distilled water, 12 g of ammonium molybdate, 0.5 g of cerium sulfate and 15 mL of concentrated sulphuric acid). Column chromatography was performed on silica gel (200–400 mesh). ^1^H, ^19^F, ^13^C and ^31^P-NMR spectra were recorded using 500, 470, 125 and 202.4 MHz NMR spectrometers (Bruker, Mannheim, Germany), respectively. Proton chemical shifts (δ) are relative to tetramethylsilane (TMS, δ = 0.0) as internal standard and expressed in parts per million (ppm). Spin multiplicities were given as s (singlet), d (doublet), t (triplet), (qt) quartet, dd (doublet of doublet), dt (doublet of triplet), ddd (doublet of doublet of doublet) and m (multiplet). Coupling constants (J) are given in Hertz (Hz). The assignment of protons and carbons were done using two-dimensional spectroscopic techniques COSY, HMQC and HMBC. IR spectra were recorded using IR Prestige-21 (Shimadzu) spectrometer. Melting points were determined by using SMP-30 melting point apparatus (Stuart Scientific, Staffordshire, UK). Elemental analyses were done on Elementar, vario MICRO cube elemental analyzer (Elementar Americas, Ronkonkoma, NY, USA). Ion-exchange chromatography was performed on Biologic DuoFlow (Bio-Rad, Hercules, CA, USA) by using Q-Sepharose as Fast Flow resin, 2 M triethylammonium bicarbonate (TEAB) buffer and MilliQ water were used as gradient solvents. Phosphate analysis was done using Briggs and Ames phosphate assays. X-ray intensity data measurements of freshly grown crystals were carried out at 298K on a Bruker-KAPPA APEX II CCD (Bruker, Mannheim, Germany)diffractometer with graphite monochromatized (MoK = 0.71073 Å) radiation. The X-ray generator was operated at 50 kV and 30 mA. Data were collected with scan width of 0.3° at different settings of φ (0°, 90° and 180°), keeping the sample to detector distance fixed at 40 mm. The X-ray data collection was monitored by SMART program (Bruker, 2003) [64]. All the data were corrected for Lorentzian, polarization and absorption effects using SAINT and SADABS programs (Bruker, 2003). SHELX-97 was used for structure solution and full matrix least-squares refinement on F2 [65]. All the hydrogen atoms were placed in geometrically idealized position and refined in the riding model approximation with C-H = 0.95 Å, and with Uiso (H) set to 1.2Ueq (C). Molecular diagrams were generated using ORTEP. Geometrical calculations were performed using SHELXTL (Bruker, 2003) and PLATON. Statistical analysis was performed using GraphPad Prism 5 (GraphPad Software, San Diego, CA, USA). Results are represented as mean ± SEM. The differences between multiple groups were analyzed by one-way ANOVA, using Tukey’s multiple comparison test. *p* ≤ 0.05 is treated as statistically significant.

### 3.2. Synthesis

#### 3.2.1. Synthesis of *Scyllo*-Inositol (**2**)

To a solution of compound **1 [45]** (1 g, 2.33 mmol) in ethanol (15 mL), 1 N HCl solution (5 mL) was added and refluxed for 1h. After completion of the reaction, the reaction mass was filtered and the resultant solid was washed with DCM, and dried under reduced pressure, to get compound **2** (0.3 g, 72%) as a white solid. ^1^H-NMR of compound **2** was identical with the reported data [46].

#### 3.2.2. Synthesis of 1,2,3,4,5,6-Hexakis-*O*-(dibenzyloxyphosphoryl)-*Scyllo*-Inositol (**3**)

Dibenzyl *N*,*N*-diisopropylphosphoramidite (2.3 g, 6.66 mmol) was added to a mixture of *scyllo*-inositol (**2**) (100 mg, 0.55 mmol) and 1*H*-tetrazole (0.46 g, 6.66 mmol) in acetonitrile (10 mL). The mixture was stirred for 15 h at room temperature. After 15 h, temperature of the reaction mixture was cooled down to −78 °C, then *m*-CPBA (1.3 g, 8.3 mmol) was added and the reaction mixture was slowly warmed to room temperature. The reaction mass was extracted with ethyl acetate and washed successfully with sat. aq. Na_2_SO_3_, sat. aq. NaHCO_3_, water and brine. The organic layer was dried over anhydrous Na_2_SO_4_ and the solvent was evaporated under reduced pressure. The crude product thus obtained was purified by column chromatography using ethyl acetate and petroleum ether (1:1, *v*/*v*) as eluent, to get compound **3** (380 mg, 0.22 mmol, 40%) as sticky liquid. ^1^H and ^31^P-NMR of compound **3** were identical with the reported data [46].

#### 3.2.3. Synthesis of *Scyllo*-Inositol Hexakisphosphate (**4**)

To a solution of 1,2,3,4,5,6-hexakis-*O*-(dibenzyloxyphosphoryl)-*scyllo*-inositol (**3**) (320 mg, 0.18 mmol) in a mixture of MeOH/H_2_O (2:1, *v*/*v*, 10 mL), 10% Pd/C (100 mg) was added at room temperature and stirred under hydrogen atmosphere (using balloon) for 10 h. After completion of the reaction, the reaction mass was filtered through a PTFE filter and the filtrate was concentrated under reduced pressure. Then, the residue was purified by ion-exchange chromatography on Q-sepharose Fast Flow resin eluting with a gradient of aqueous TEAB (0 to 1.0 M) to obtain the triethylammonium salt of *scyllo*-inositol hexakisphosphate (**4**, 104 mg, 0.16 mmol, 89%) as a white foamy solid. ^1^H and ^31^P-NMR of the compound were identical with the reported data [46].

#### 3.2.4. Synthesis of 6-*O*-Benzoyl-2,4-Di-*O*-(p-Methoxybenzyl)-1,3,5-*O*-Methylidyne-*Scyllo*-Inositol (**5**)

To a solution of compound **1** (1 g, 2.33 mmol) in dry pyridine (20 mL) at 0 °C, benzoyl chloride (0.4 mL, 3.48 mmol) and DMAP (50 mg, 0.41 mmol) were added and stirred for 4–5 h. After completion of the reaction, water was added to quench the excess benzoyl chloride. The crude reaction mass was extracted with ethyl acetate and then washed thoroughly with saturated NaHCO_3_ solution, water and brine. The organic layer was dried over anhydrous Na_2_SO_4_ and the solvent was evaporated under reduced pressure. The crude product thus obtained was purified by column chromatography using ethyl acetate and petroleum ether (2:8, *v*/*v*) as eluent, to get compound **5** (1.2 g, 96%) as a white solid. The m. p. = 92 °C; RF = 0.7 (ethyl acetate/petroleum ether, 3:7, *v*/*v*); 1H-NMR (500 MHz, CDCl_3_) δ: 3.69 (s, 6H, 2 x OCH_3_), 4.31 (brs, 2H), 4.37 (d, *J* = 10.4 Hz, 2H, ArCH_2_), 4.48 (d, *J* = 10.4 Hz, 2H, ArCH_2_), 4.56 (brs, 3H), 5.52 (s, 1H), 5.57 (brs, 1H), 6.59 (d, *J* = 8.2 Hz, 4H, ArH), 6.96–7.0 (m, 6H, ArH), 7.33 (t, *J* = 7.4 Hz, 1H, ArH), 7.74 (d, *J* = 7.7 Hz, 2H, ArH). 13C-NMR (CDCl_3_, 125 MHz) δ: 55.2, 66.6, 67.9, 68.4, 71.6 (ArCH_2_), 72.7, 103.1, 113.6, 128.0, 128.5, 129.5, 129.6, 129.62, 130.2, 130.3, 132.8, 159.1, 166.1. Elemental analysis Calcd for C_30_H_30_O_9_: C, 67.41; H, 5.66. Found C, 67.23; H, 5.75.

#### 3.2.5. Synthesis of 1-*O*-Benzoyl-*Scyllo*-Inositol (**6**)

To a solution of compound **5** (1.0 g, 1.87 mmol) in ethanol (15 mL), 1 N HCl (5 mL) was added and refluxed for 1h. After completion of the reaction, the reaction mass was filtered and the obtained solid was washed with DCM and dried under reduced pressure to get compound **6** as a white solid (0.42 g, 79%). ^1^H-NMR of compound **6** was identical with the reported data [46].

#### 3.2.6. Synthesis of 6-*O*-Benzoyl-1,2,3,4,5-Pentakis-*O*-(Dibenzyloxyphosphoryl)-*Scyllo*-Inositol (**7**)

Dibenzyl *N*,*N*-diisopropylphosphoramidite (0.49 g, 1.41 mmol) was added to a mixture of 1-O-benzoy-*scyllo*-inositol (**6**) (40 mg, 0.14 mmol) and 1*H*-tetrazole (99 mg, 1.41 mmol) in dry acetonitrile (10 mL). The mixture was stirred for 15 h at room temperature. After 15 h, temperature of the reaction mixture was cooled down to −78 °C, then *m*-CPBA (60%, 0.33 g, 2.1 mmol) was added and the reaction mixture was slowly warmed to room temperature. The reaction mass was extracted with ethyl acetate and washed successfully with sat. aq. Na_2_SO_3_, sat. aq. NaHCO_3_, water and brine. The organic layer was dried over anhydrous Na_2_SO_4_ and the solvent was evaporated under reduced pressure. The crude product thus obtained was purified by column chromatography using ethyl acetate and petroleum ether (1:1, *v*/*v*) as eluent, to get compound **7** (0.2 g, 0.13 mmol, 93%) as a sticky liquid; ^1^H-NMR (500 MHz, CDCl_3_) δ: 4.46 (dd, *J* = 9.3 Hz, 11.7 Hz, 2H, ArC*H*_2_), 4.67 (d, *J* = 7.4 Hz, 1H, ArC*H*A), 4.70 (d, *J* = 7.4 Hz, 1H, ArC*H*B), 4.74 (d, *J* = 8.1 Hz, 1H, ArC*H*A), 4.76 (d, *J* = 8.1 Hz, 1H, ArC*H*B), 5.01–4.77 (m, 19H, ArC*H*_2_ & H-2, H-3, H-4, H-5, H-6), 5.69 (t, *J* = 7.6 Hz, 1H, H-1), 6.85 (m, 4H, Ar*H*), 6.98 (m, 4H, Ar*H*), 7.05–7.23 (m, 42H, Ar*H*), 7.23 (t, *J* = 7.9 Hz, 1H, Ar*H*), 7.42–7.38 (td, *J* = 1.2 Hz, 7.5 Hz, 1H, Ar*H*), 7.98 (dd, *J* = 1.2 Hz, 8.3 Hz, 1H, Ar*H*). ^13^C-NMR (CDCl_3_, 125 MHz) δ: 69.4, 69.5, 69.6, 69.7, 69.9, 69.92, 69.97, 70.0, 70.04, 72.1, 75.9 (^31^P coupled), 76.3 (^31^P coupled), 127.7, 127.9, 128.1, 128.15, 128.2, 128.28, 128.31, 128.35, 128.4, 128.42, 128.44, 129.3, 130.2, 133.2, 135.3, 135.37, 135.6, 135.66, 135.7, 165.2. ^31^P-NMR (202.4 MHz, CDCl_3_) δ: −1.77 (2P), −1.84 (3P). Elemental analysis Calcd for C_83_H_81_O_22_P_5_: C, 62.88; H, 5.15. Found C, 62.76; H, 5.32.

#### 3.2.7. Synthesis of 6-*O*-Benzoyl-*Scyllo*-Inositol-1,2,3,4,5-Pentakisphosphate (**8**)

To a solution of 6-*O*-benzoyl-1,2,3,4,5-pentakis-*O*-(dibenzyloxyphosphoryl)-*scyllo*-inositol (**7**) (160 mg, 0.1 mmol) in a mixture of MeOH/H_2_O (2:1, *v*/*v*, 15 mL), 10% Pd/C (50 mg) was added at room temperature and stirred under hydrogen atmosphere (using balloon) for 10 h. After completion of the reaction, the reaction mass was filtered through a PTFE filter and the filtrate was concentrated under reduced pressure. Then the residue was purified by ion-exchange chromatography on Q-sepharose Fast Flow resin eluting with a gradient of aqueous TEAB (0 to 1.0 M) to obtain the triethylammonium salt of 1-*O*-benzoyl-*scyllo*-inositol-2,3,4,5,6-pentakisphosphate (**8**) (50 mg, 0.073 mmol, 73%) as a white foam; ^1^H-NMR (500 MHz, CDCl_3_) δ: 4.44–4.36 (m, 3H, H-3,H-4, H-5), 4.51 (qt, *J* = 9.3 Hz, 2H, H-2, H-6), 5.53 (t, *J* = 9.6 Hz, 1H, H-1), 7.4 (t, *J* = 7.4 Hz, 2H, Ar*H*), 7.53 (t, *J* = 7.5 Hz, 1H, Ar*H*), 7.95 (t, *J* = 7.9 Hz, 2H, Ar*H*). ^13^C-NMR (CDCl_3_, 125 MHz) δ: 71.8 (C-1), 75.3 (C-2, C-6), 76.3 (C-4), 76.4 (C-3, C-5), 128.5, 128.9, 129.8, 133.9, 167.7. ^31^P-NMR (202.4 MHz, CDCl_3_) δ: 2.12 (1P), 1.91 (2P), 1.61 (2P). Elemental analysis Calcd for C_13_H_21_O_22_P_5_: C, 22.82; H, 3.09. Found C, 22.75; H, 3.33.

#### 3.2.8. Synthesis of *Scyllo*-Inositol-1,2,3,4,5-Pentakisphosphate (**9**)

The 1 N LiOH (5 mL) was added to 6-*O*-benzoyl-*scyllo*-inositol-1,2,3,4,5-pentakisphosphate (**8**) (50 mg, 0.073 mmol) and heated at 80 °C for 3 h. After completion of the reaction the reaction mass was passed through Dowex 50WX8-100 (H+ form) and eluted with water. The filtrate was concentrated under reduced pressure to obtain compound **9** (34 mg, 0.058 mmol, 80%) as a sticky solid. ^1^H and ^31^P-NMR of the compound were identical with the reported data [46].

#### 3.2.9. Synthesis of 1-*O*-*p*-Methoxybenzyl-3,4,5,6-Tetra-*O*-Benzoyl-*Myo*-Inositol (**11**)

To a solution of 1,4,5,6-tetra-*O*-benzoyl-*myo*-inositol (**10**) [50] (7 g, 11.7 mmol) in dry acetonitrile (150 mL), dibutyltinoxide (3.2 g, 12.87 mmol) and tetrabutylammonium bromide (6.49 g, 17.6 mmol) were added at room temperature and the mixture was refluxed for 3–4 h. The reaction mass was then brought to room temperature, *p*-methoxybenzylchloride (2.76 g, 17.6 mmol) was added and heated at 60 °C for 12 h. After completion of the reaction, Et_3_N (1.18 g, 23.4 mmol) was added and refluxed for 1 h. Then the reaction mass was extracted with ethyl acetate, washed successively with sat. aq. NaHCO_3_ solution, water and brine. The organic layer was dried over anhydrous Na_2_SO_4_ and the solvent was evaporated under reduced pressure. The crude product thus obtained was purified by column chromatography using a mixture of ethyl acetate and petroleum ether (3:7, *v*/*v*) as eluent, to get compound **11** (7.04 g, 9.82 mmol, 84%) as a white solid. The m. p. = 188 °C; R_f_ = 0.6 (ethyl acetate/petroleum ether, 3:7, *v*/*v*); ^1^H-NMR (500 MHz, CDCl_3_) δ: 2.73 (s, 1H, 2-OH), 3.67 (s, 3H, OC*H*_3_), 3.82 (dd, *J =* 2.2 Hz, 9.75 Hz, 1H, H-1), 4.40 (d, *J* = 11.8 HZ, 1H, C*H*_2_), 4.58 (m, 2H, ArC*H*_2_ & H-2), 5.23 (dd, *J* = 1.9 Hz, 10.3 Hz, 1H, H-3), 5.70 (t, *J* = 10 Hz, 1H, H-5), 5.98 (t, *J* = 9.9 Hz, 1H, H-6), 6.23 (t, *J* = 10.2 Hz, 1H, H-4), 6.59 (d, *J* = 6.6 Hz, 2H, Ar*H* of PMB), 7.0 (d, *J* = 8.5 Hz, 2H, Ar*H* of PMB), 7.21 (m, 3H), 7.34–7.29 (m, 7H), 7.47–7.43 (m, 2H), 7.70 (t, *J* = 7.6 Hz, 4H), 7.82 (d, *J* = 7.3 Hz, 2H), 7.93 (d, *J* = 7.3 Hz, 2H). ^13^C-NMR (CDCl_3_, 125 MHz) δ: 55.2, 67.4 (C-2), 69.8 (C-4), 71.2 (C-5), 71.3 (C-6), 71.9 (Ar*C*H_2_), 72.1 (C-3), 76.3 (C-1), 113.9, 128.2, 128.3, 128.5, 128.7, 128.8, 129.0, 129.2, 129.5, 129.6, 129.72, 129.76, 129.8, 129.9, 133.0, 133.1, 133.2, 133.5, 159.6, 165.4, 165.5, 165.8, 166.0. Elemental analysis Calcd for C_42_H_36_O_11_: C, 70.38; H, 5.06. Found C, 70.49; H, 4.82.

#### 3.2.10. Synthesis of 1-*O*-*p*-Methoxybenzyl-3,4,5,6-Tetra-*O*-Benzoyl-2-*O*-Trifluoromethanesulfonyl-*Myo*-Inositol (**12**)

To a solution of 1-*O*-*p*-methoxybenzyl-3,4,5,6-tetra-*O*-benzoyl-*myo*-inositol (**11**) (7.0 g, 9.76 mmol) in a mixture of dry DCM (50 mL) and dry pyridine (20 mL), triflic anhydride (5.51 g, 19.5 mmol) was added at 0 °C. After completion of the reaction, reaction mass was extracted with DCM and the organic layer was washed successfully with sat. aq. NaHCO_3_, water and brine. The organic layer was dried over anhydrous Na_2_SO_4_ and the solvent was evaporated under reduced pressure. The crude product thus obtained was purified by column chromatography using a mixture of ethyl acetate and petroleum ether (2:8, *v*/*v*) as eluent, to get compound **12** (5.38 g, 6.34 mmol, 65%) as a white solid; m. p. = 126 °C; ^19^ F NMR (470 MHz, CDCl_3_) δ: −76.0. ^1^H-NMR (500 MHz, CDCl_3_) δ: 3.68 (s, 3H, OC*H*_3_), 3.97 (dd, *J =* 1.9 Hz, 10.2 Hz, 1H, H-1), 4.34 (d, *J* = 12.2 Hz, 1H, ArC*H*_A_), 4.70 (d, *J* = 12.2 Hz, 1H, ArC*H*_B_), 5.44 (dd, *J* = 2.1 Hz, 10.5 Hz, 1H, H-3), 5.59 (brs, 1H, H-2), 5.72 (t, *J* = 10.1 Hz, 1H, H-5), 5.89 (t, *J* = 10.1 Hz, 1H, H-6), 6.09 (t, *J* = 10.27 Hz, 1H, H-4), 6.58 (d, *J* = 8.5 Hz, 2H, Ar*H* of PMB), 7.0 (d, *J* = 8.5 Hz, 2H, Ar*H* of PMB), 7.17–7.21 (m, 1H), 7.29–7.35 (m, 8H), 7.47 (m, 3H), 7.73 (t, *J* = 6.9 Hz, 4H), 7.78 (d, *J* = 7.3 Hz, 2H), 7.92 (d, *J* = 7.3 Hz, 2H). ^13^C-NMR (CDCl_3_, 125 MHz) δ: 55.0, 55.2, 69.2 (C-3), 69.3 (C-4), 70.1 (C-6), 70.6 (C-5), 72.0 (Ar*C*H_2_), 72.9 (C-1), 82.0 (C-2), 113.9, 127.8, 128.0, 128.3, 128.4, 128.5, 128.6, 129.1, 129.7, 129.8, 129.9, 130.0, 130.1, 133.3, 133.36, 133.4, 134.0, 159.7, 164.9, 165.1, 165.6, 165.7. Elemental analysis Calcd. for C_43_H_35_F_3_O_13_S: C, 60.85; H, 4.16. Found C, 60.68; H, 4.11.

#### 3.2.11. Synthesis of 2-O-Acetyl-1-*O*-*p*-Methoxybenzyl-3,4,5,6-Tetra-*O*-Benzoyl-*Scyllo*-Inositol (**13**)

To a solution of 1-*O*-*p*-methoxybenzyl-3,4,5,6-tetra-*O*-benzoyl-2-*O*-trifluoromethanesulfonyl-*myo*-inositol (**12**) (5 g, 5.89 mmol) in *N*,*N*-dimethylacetamide (30 mL), potassium acetate (2.89 g, 29.5 mmol) was added and heated at 70 °C for 2 h. After completion of the reaction, the reaction mass was extracted with ethyl acetate and thoroughly washed with water and brine. The organic layer was dried over anhydrous Na_2_SO_4_ and the solvent was evaporated under reduced pressure. The crude product thus obtained was purified by column chromatography using a mixture of ethyl acetate and petroleum ether (2:8, *v*/*v*) as eluent, to get compound **13** (4.25 g, 5.59 mmol, 95%) as a white solid; m. p. = 254 °C; ^1^H-NMR (500 MHz, CDCl_3_) δ: 1.83 (s, 3H, OAc), 3.64 (s, 3H, OC*H*_3_), 3.99 (t, *J =* 9.2 Hz, 1H, H-1), 4.47 (d, *J* = 11.8 Hz, 1H, ArC*H*_A_), 4.52 (d, *J* = 11.7 Hz, 1H, ArC*H*_B_), 5.55 (t, *J* = 9.7 Hz, 1H, H-6), 5.65 (t, *J =* 9.7 Hz, 1H, H-5), 5.82–5.71 (m, 3H, H-2, H-3, H-4), 6.59 (d, *J* = 8.5 Hz, 2H, Ar*H* of PMB), 6.96 (d, *J* = 8.5 Hz, 2H, Ar*H* of PMB), 7.33–7.27 (m, 7H), 7.46–7.42 (m, 2H), 7.70 (d, *J =* 7.5 Hz, 4H), 7.82 (t, *J* = 6.6 Hz, 4H). ^13^C-NMR (CDCl_3_, 125 MHz) δ: 20.6, 55.2, 70.6 (C-2 or C-3 or C-4 or C-5), 70.7 (C-3 or C-4 or C-5 or C-2), 71.0 (C-4 or C-5 or C-2 or C-3), 71.9 (C-6), 72.2 (C-5 or C-2 or C-3 or C-4), 74.5 (Ar*C*H_2_), 77.2 (C-1), 113.7, 128.2, 128.3, 128.4, 128.5, 128.6, 128.7, 129.1, 129.2, 129.7, 129.8, 129.9, 133.2, 133.3, 133.5, 159.3, 165.2, 165.3, 165.5, 165.6, 169.4. Elemental analysis Calcd. for C_44_H_38_O_12_: C, 69.65; H, 5.05. Found C, 69.59; H, 4.83.

#### 3.2.12. Synthesis of 1-*O*-*p*-Methoxybenzyl-*Scyllo*-Inositol (**14**)

To a solution of 2-*O*-acetyl-1-*O*-*p*-methoxybenzyl-3,4,5,6-tetra-*O*-benzoyl-*scyllo*-inositol (4.0 g, 5.27 mmol) in methanol (50 mL), sodium methoxide (57 mg, 1.05 mmol) was added and refluxed for 4 h. After completion of the reaction, Dowex^®^ 50WX8 H^+^ (1 g) was added to the reaction mass and stirred at room temperature for 10 min. Then, the mixture was filtered and the resin was washed with methanol. The combined filtrate was evaporated under reduced pressure to get the title compound **14** as a white solid (1.55 g, 5.16 mmol, 98%). Charred at 220 °C without melting; ^1^H-NMR (500 MHz, CDCl_3_) δ: 2.94–3.04 (m, 4H), 3.12–3.17 (m, 2H), 3.73 (s, 3H, OC*H*_3_), 4.69 (s, 2H, ArC*H*_2_), 4.81 (brs, 5H, OH), 6.86 (d, *J* = 8.5 Hz, 2H, Ar*H*), 7.33 (d, *J* = 8.5 Hz, 2H, Ar*H*). ^13^C-NMR (CDCl_3_, 125 MHz) δ: 55.5, 73.7, 74.4, 74.5, 74.8, 83.3, 113.7, 129.6, 132.2, 158.8. Elemental analysis Calcd for C_14_H_20_O_7_: C, 55.99; H, 6.71. Found C, 55.82; H, 6.65.

#### 3.2.13. Synthesis of 6-*O*-*p*-Methoxybenzyl-1,2,3,4,5-Pentakis-*O*-(Dibenzyloxyphosphoryl)-*Scyllo*-Inositol (**15**)

Dibenzyl *N,N*-diisopropylphosphoramidite (5.76 g, 16.7 mmol) was added to a solution of 1-*O*-*p*-methoxybenzyl-*scyllo*-inositol (**14**) (0.5 g, 1.67 mmol) and 1*H*-tetrazole (1.17 g, 16.7 mmol) in dry acetonitrile (30 mL). The mixture was stirred for 15 h at room temperature. After 15 h, the reaction mixture was cooled down to −78 °C, then *m*-CPBA (3.92 g, 25.05 mmol) was added and the reaction mixture was slowly warmed to room temperature. The reaction mass was extracted with ethyl acetate and washed successively with sat. aq. Na_2_SO_3_, sat. aq. NaHCO_3_, water and brine. The organic layer was dried over anhydrous Na_2_SO_4_ and the solvent was evaporated under reduced pressure. The crude product thus obtained was purified by column chromatography using a mixture of ethyl acetate and petroleum ether (1:1, *v*/*v*) as eluent, to get compound **15** (2.4 g, 15.03 mmol, 90%) as a sticky liquid. ^1^H-NMR (500 MHz, CDCl_3_) δ: 3.56 (s, 3H, OC*H*_3_), 4.06 (t, *J* = 4.5 Hz, 1H), 4.4 (brs, 2H), 4.80–4.99 (m, 25H), 6.56 (d, *J* = 8.5 Hz, 2H), 7.1–7.2 (m, 52H). ^13^C-NMR (CDCl_3_, 125 MHz) δ: 55.1, 69.6, 69.7, 69.8, 69.9, 72.5, 76.9 (^31^P coupled), 77.3 (^31^P coupled), 79.3 (^31^P coupled), 113.6, 128.0, 128.1, 128.3, 128.4, 128.5, 129.3, 129.5, 135.57, 135.63, 135.65, 135.68, 135.7, 135.71, 135.73, 159.1. ^31^P-NMR (202.4 MHz, CDCl_3_) δ: −1.9, −2.0. Elemental analysis Calcd for C_84_H_85_O_22_P_5_: C, 63.00; H, 5.35. Found C, 62.82; H, 5.61.

#### 3.2.14. Synthesis of 1,2,3,4,5-Pentakis-*O*-(Dibenzyloxyphosphoryl)-*Scyllo*-Inositol (**16**)

To a solution of 6-*O-p*-methoxybenzyl-1,2,3,4,5-pentakis-*O*-(dibenzyloxyphosphoryl)-*scyllo*-inositol (**15**) (1 g, 0.62 mmol) in anhydrous 1,2-dichloroethane (20 mL), DDQ (170.0 mg, 0.75 mmol) was added and heated at 60 °C for 3–4 h. After completion of the reaction, the reaction mass was concentrated under reduced pressure and the crude residue was purified by column chromatography using a mixture of ethyl acetate and petroleum ether (1:1, *v*/*v*) as eluent to get compound **16** (670 mg, 0.45 mmol, 73%) as a white solid. The m. p. = 112 °C; ^1^H-NMR (500 MHz, CDCl_3_) δ: 3.98 (td, *J* = 2.5 Hz, 8.2 Hz, 1H, H-1), 4.65 (qt, *J* = 8.1 Hz, 2H, H-2 & H-6), 4.65 (m, 1H, H-4), 4.73 (qt, *J* = 7.8 Hz, 2H, H-3 & H-5), 4.91–5.13 (m, 20H), 5.29 (d, *J =* 2.6 Hz, 1H, OH-1), 7.15–7.29 (m, 50H). ^13^C-NMR (CDCl_3_, 125 MHz) δ: 69.64, 69.68, 69.72, 69.77, 69.85, 69.89, 69.96, 70.01, 70.05, 70.09, 72.4 (C-1), 75.4 (C-4, ^31^P coupled), 75.9 (C-3 & C-5, ^31^P coupled), 79.1 (C-2 & C-6, ^31^P coupled), 127.9, 128.03, 128.07, 128.1, 128.3, 128.33, 128.38, 128.4, 128.47, 128.5, 135.5, 135.62, 135.69, 135.71, 135.76, 135.8, 135.86. ^31^P-NMR (202.4 MHz, CDCl_3_) δ: −1.55, −1.50, −0.79. Elemental analysis Calcd for C_76_H_77_O_21_P_5_: C, 61.62; H, 5.24. Found C, 61.85; H, 4.87.

#### 3.2.15. Crystal Data for **16**

**Figure 7:**
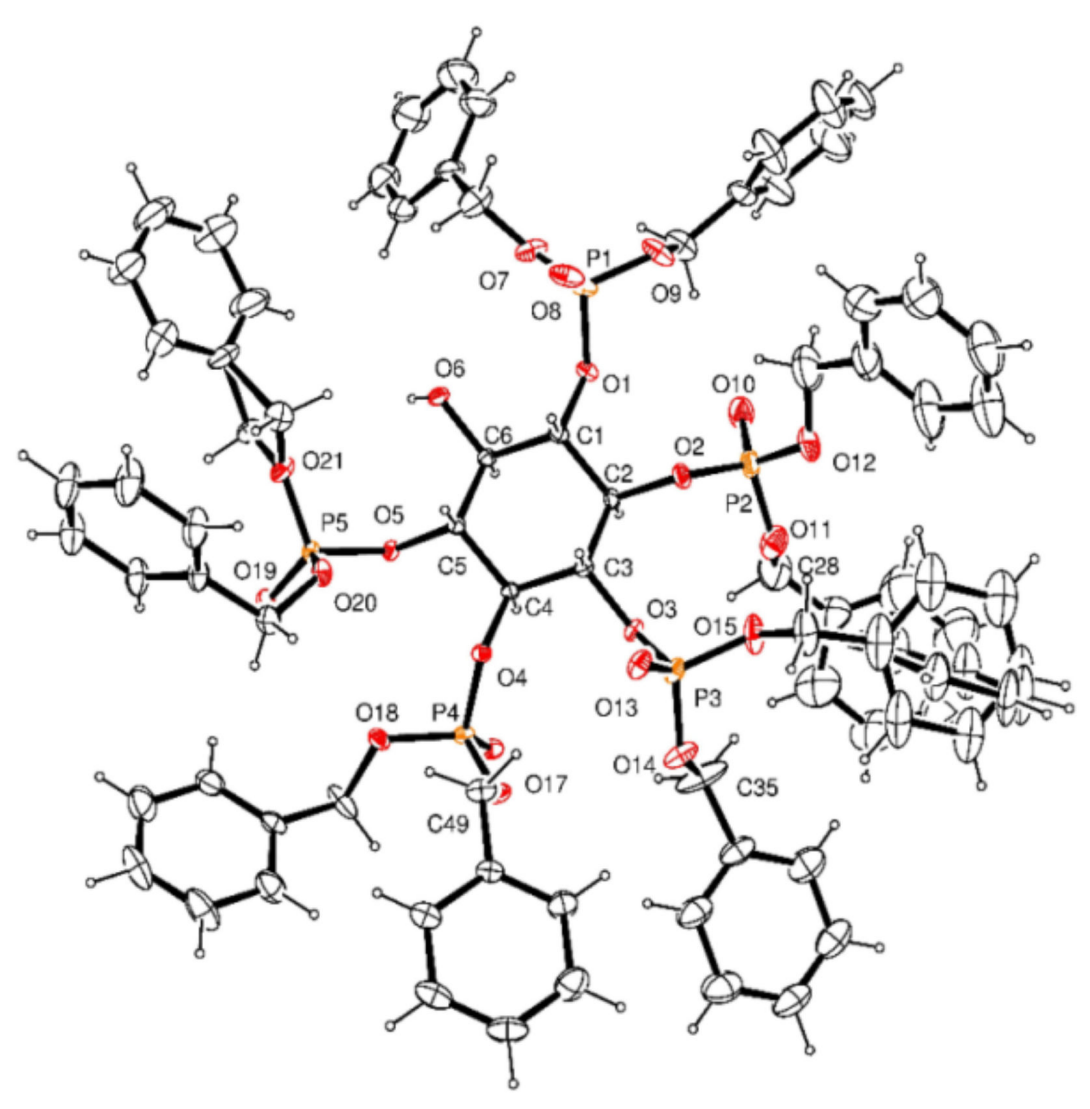
ORTEP diagram of compound **16** at 30% probability level.

CCDC No. 1946543 (Figure 7) Molecular formula = C_76_H_77_O_21_P_5_, Formula weight = 1481.23, colorless blocks, 0.25 × 0.20 × 0.15 mm, Triclinic, space group P-1, 12.9915(9), b = 16.3817(11), c = 19.4914(14) Å, V = 3759.2(5) Å^3^, Z = 2, T = 293(2) K, 2θmax = 50.00°, D calc (g cm^−3^) = 1.309, F(000) = 1552, μ (mm^−1^) = 0.194, 55445 reflections measured, 13192 unique reflections (R_int_ = 0.0907), multi-scan absorption correction, T_min_ = 0.9530, T_max_ = 0.9714, number of parameters = 847, number of restraints = 396, GoF = 0.986, R1 = 0.0884, wR2 = 0.2343, R indices based on 13192 reflections with I > 2s(I). Δρmax = 0.555, Δρmin = −0.358 (eÅ^−3^).

#### 3.2.16. Synthesis of 2-Cyanoethyl-*N*,*N*-Diisopropylbenzylphosphoramidite

Diisopropylethylamine (0.87 mL, 5.0 mmol) was added to a mixture of chloro-2-cyanoethyl-*N*,*N*-diisopropylphosphoramidite (1.1 mL, 5.0 mmol) and benzyl alcohol (0.52 mL, 5.0 mmol) at room temperature and stirred for 1 h (Scheme 3). The reaction mixture was extracted with DCM, washed with sat. aq. NaHCO_3_ and the organic layer was dried over anhydrous Na_2_SO_4_. The organic layer was concentrated under reduced pressure and used without any further purification. ^1^H and ^31^P-NMR of the compound (1.4 g, 4.54 mmol, 91%) were identical with the reported data [66].

#### 3.2.17. Synthesis of 6-((Benzyloxy)(2-Cyanoethoxy)Phosphoryl)-1,2,3,4,5-Pentakis-*O*-(Dibenzyloxyphosphoryl)-*Scyllo*-Inositol (**17**)

To a solution of 1,2,3,4,5-pentakis-*O*-(dibenzyloxyphosphoryl)-*scyllo*-inositol (**16**) (0.2 g, 0.135 mmol) in dry DCM (10 mL), 2-cyanoethyl *N*,*N*-diisopropylbenzylphosphoramidite (0.41 g, 1.35 mmol) and 1*H*-tetrazole (0.094 g, 1.35 mmol) were added. The mixture was stirred for 4 h at room temperature and then *m*-CPBA (0.32 g, 2.03 mmol) was added at −78 °C and the reaction mixture was slowly warmed to room temperature. The reaction mass was then extracted with ethyl acetate and washed successively with sat. aq. Na_2_SO_3_, sat. aq. NaHCO_3_, water and brine. The organic layer was dried over anhydrous Na_2_SO_4_ and the solvent was evaporated under reduced pressure. The crude product thus obtained was purified through column chromatography using a mixture of acetone and petroleum ether (3:7, *v*/*v*) as eluent, to get compound **17** (160 mg, 0.09 mmol, 67%) as a sticky liquid; ^1^H-NMR (500 MHz, CDCl_3_) δ: 2.29 (t, *J =* 6.4 Hz, 2H), 3.93 (m, 2H), 4.87–5.08 (m, 28H), 7.12–7.20 (m, 55H). ^13^C-NMR (CDCl_3_, 125 MHz) δ: 62.4, 62.5, 69.9, 70.0, 70.1, 70.3, 70.4, 76.3, 76.4, 116.6, 128.0, 128.11, 128.19, 128.3, 128.5, 128.52, 128.6, 128.66, 128.7, 128.8, 135.2, 135.3, 135.52, 135.58. ^31^P-NMR (202.4 MHz, CDCl_3_) δ: −1.98, −2.78. Elemental analysis Calcd for C_86_H_87_NO_24_P_6_: C, 60.60; H, 5.14; N, 0.82. Found C, 60.53; H, 5.27; N. 0.88.

#### 3.2.18. Synthesis of 6-*O*-Benzyloxyphosphoryl-1,2,3,4,5-Pentakis-*O*-(Dibenzyloxyphosphoryl)-*Scyllo*-Inositol (**18**)

To a solution of 6-((benzyloxy)(2-cyanoethoxy)phosphoryl)-1,2,3,4,5-pentakis-*O*-(dibenzyloxyphosphoryl)-*scyllo*-inositol (**17**) (0.1 g, 0.059 mmol) in dry acetonitrile (1.0 mL), DBU (35 μL, 0.24 mmol) was added followed by the addition of bis(trimethylsilyl)trifluoroacetamide (0.5 mL) under nitrogen atmosphere. After 1 h, the reaction mixture was filtered through Dowex-H^+^ resin. The filtrate was concentrated under reduced pressure and the crude reaction mass was purified by column chromatography using methanol and dichloromethane (1:9, *v*/*v*) as eluent to afford compound **18** (88 mg, 0.053 mmol, 90%) as a sticky solid; ^1^H-NMR [500 MHz, CDCl_3_:CD_3_OD (3:1)] δ: 1.61–1.70 (m, 5H), 2.48–2.50 (m, 2H), 3.31–3.37 (m, 4H), 3.75 (m, 1H), 4.77–4.88 (m, 24H), 4.93 (m, 2H), 5.17 (m, 2H), 6.99–7.13 (m, 55H). ^13^C-NMR (CDCl_3_:CD_3_OD (3:1), 125 MHz) δ: 23.1, 26.5, 28.1, 29.7, 31.8, 36.1, 36.7, 45.3, 49.9, 67.6, 67.7, 69.8, 69.9, 69.98, 70.0, 70.2, 70.3, 70.36, 70.4, 74.7 (^31^P coupled), 76.8 (^31^P coupled), 77.0 (^31^P coupled), 77.5 (^31^P coupled), 112.4, 114.6, 117.0, 119.2, 127.3, 127.4, 127.9, 127.98, 128.0, 128.1, 128.3, 128.4, 128.47, 134.9, 135.0, 135.07, 135.2, 135.22, 135.26, 137.5, 159.1, 159.4, 159.7, 159.8, 160.0, 177.9. ^31^P-NMR (202.4 MHz, CDCl_3_) δ: −1.92, −2.55, −3.03, −3.0. Elemental analysis Calcd for C_83_H_84_NO_24_P_6_: C, 60.37; H, 5.13. Found C, 60.31; H, 5.19.

#### 3.2.19. Synthesis of 6-*O*-Diphospho-*Scyllo*-Inositol-1,2,3,4,5-Pentakisphosphate (*Scyllo*-IP7) (**20**)

To a solution of 6-*O*-benzyloxyphosphoryl-1,2,3,4,5-pentakis-*O*-(dibenzyloxyphosphoryl)-*scyllo*-inositol (**18**) (50 mg, 0.03 mmol) and trimethylamine (9 µL, 0.06 mmol) in anhydrous DCM, dibenzylphosphoryl chloride (0.18 mL, 10% in toluene, 0.06 mmol) was added at 0 °C. After 2 h, the solvents were removed by applying vacuum, and the residue was completely dried at 10^−2^ mbar. The residue (**19**) was dissolved in *t*-BuOH/H_2_O (6:1, *v*/*v*, 14 mL) to which 10% Pd/C (100 mg) was added and stirred under hydrogen atmosphere (balloon pressure) for 10 h. The catalyst was removed by filtration through a PTFE syringe filter and the filtrate was concentrated under reduced pressure. The obtained residue was purified by ion-exchange chromatography on Q-sepharose Fast Flow resin eluting with a gradient of triethylammonium bicarbonate (TEAB) buffer (0 to 2.0 M) and water to obtain the triethylammonium salt of **20** (10 mg, 44%) as a white solid. ^31^P-NMR (202.4 MHz, D_2_O) δ: 1.99 (5P), −6.1 (1P), −11.0 (1P); HRMS (ESI) mass calculated for C_6_H_19_O_27_P_7_ [M − H]^+^, 738.8199, found 738.5057.

### 3.3. Generation and Detection of PPIPn Products

The 6xHis-tagged mouse IP6K1 (mIP6K1) and GST-tagged kinase domain of S. cerevisiae Vip1 (amino acid residues 1–535) (GST-VIP1-KD) were expressed and purified as described earlier [67]. Assays to detect synthesis of PPIP_n_ product were set up in reaction buffer (20 mM Tris-Cl pH 7.4, 60 mM NaCl, 6 mM MgCl_2_, 1 mM DTT) in a 25 μL reaction volume for PAGE analysis and a 50 μL reaction volume for HPLC analysis. For PAGE analysis, the reactions were supplemented with 1 mM Mg^2+^-ATP, an ATP regeneration system (6 mM phosphocreatine and 25 U/mL creatine phosphokinase), ~1.2 µM purified enzyme, and either 200 µM IPn substrate (for 6xHis-IP6K1) or 100 µM IPn substrate (for GST-VIP1 KD), and were incubated at 37 °C for 18 h. The reactions were mixed with 0.1% orange G containing loading dye and were resolved on a 35.8 % polyacrylamide gel, overnight at 500 V in 1X TBE (Tris/borate/EDTA), as described earlier [52]. The gels were stained with 0.1% Toluidine blue (*w*/*v*) in 20% methanol and 2% glycerol and were destained in 20% methanol and 2% glycerol. The gels were scanned on a desktop scanner (Laserjet M1136 MFP).

For HPLC analysis, the reactions were supplemented with 1 mM Mg^2+^-ATP and 200 µM IP_n_ substrate for 6xHis-mIP6K1, and 40 µM Mg^2+^-ATP and 100 µM IP_n_ substrate for GST-VIP1-KD. The concentration of ATP used in the assay was chosen on the basis of the reported affinities of IP6K1 and VIP1-kinase domain for ATP (Km value for ATP for mIP6K1 is 1.1 mM [68]; Km value for ATP has not been measured for S. cerevisiae VIP1, but the kinase domain of human PPIP5K2 has a Km of 20–40 µM for ATP [38]. All reactions contained 2.5 µCi [γ-^32^P]ATP (Regional Centre Hyderabad, BRIT, India) and ~1.2 µM purified enzyme, and were incubated at 37 °C for 18 h. A negative control was set up by incubating *myo*-IP_6_ in the presence of ~1 µM BSA, along with 2.5 µCi [γ-^32^P] ATP and 40 µM Mg^2+^-ATP. The reactions were terminated by the addition of 40 µL of 6 mM EDTA, followed by 100 µL of 0.6 M perchloric acid, followed by 33 µL neutralization solution (1 M potassium carbonate and 5 mM EDTA). The inositol phosphates were resolved by HPLC (515 HPLC pumps, Waters) on a SAX Partisphere column (4.6 mm diameter and 125 mm length column, Whatman) using a gradient of buffer A (1 mM EDTA) and buffer B (1 mM EDTA and 1.3 M (NH_4_)_2_HPO_4_, pH 3.8) as follows: 0–5 min, 0% B; 5–10 min, 0–20% B; 10–70 min, 20–100% B; 70–80 min, 100% B (see Supplementary Figure S2A for the elution gradient profile). One-milliliter fractions were mixed with 4 mL scintillation cocktail (Ultima-Flo AP, Perkin Elmer) and counted for 1 min in a liquid scintillation analyzer (Tri-Carb 2910 TR, Perkin Elmer). To identify the retention times for standard *myo*-IPs, we utilized radiolabelled inositol phosphates extracted from yeast strains harboring deletions of enzymes involved in inositol phosphate synthesis, as described earlier [69]. S. cerevisiae strain BY4741 wild type (WT), *ipk1*Δ, *kcs1*Δ *and vip1*Δ were grown in synthetic medium containing yeast nitrogen base with ammonium sulphate and without inositol (MP Biomedicals), 2% dextrose (Sigma-Aldrich), 76 mg/mL uracil (HiMedia Laboratories), synthetic complete supplement mixture (SCSM; Formedium) without inositol, and supplemented with 5 µCi/mL of *myo*-2-[^3^H] inositol (15–20 Ci/mmol; American Radiolabeled Chemicals). The yeast was inoculated at OD_600_ 0.001 and grown to OD_600_ ~1.0 at 30 °C, harvested by centrifugation (2000× *g*, 3 min), lysed in 350 µL chilled lysis buffer (1 M perchloric acid, 0.2 mg/mL IP_6_, 2 mM EDTA) by bead beating using glass beads (Sigma-Aldrich). The soluble cell extracts were harvested by centrifugation (21,000× *g* for 5 min) and neutralized with 1 M potassium carbonate/5 mM EDTA solution. The extracted inositol phosphates were resolved using the column and gradient described above. One-milliliter fractions were mixed with 3 mL scintillation cocktail and counted for 5 min, as described above. In this way, we were able to identify the retention time for *myo*-IP_5_, *myo*-IP_6_, *myo*-PP-IP_4_, *myo*-[PP]_2_-IP_3_, *myo*-PP-IP_5_ and *myo*-[PP]_2_-IP_4_, based on the IP profile described earlier for these strains [70] (Supplementary Figure S2B). The retention time of [γ-^32^P]ATP, determined from the negative control reaction (see Supplementary Figure S2C), was found to be ~20 min. Therefore, to avoid any carryover of ATP, fractions collected from 35–70 min of the elution gradient were monitored to identify the PPIP_n_ product. Minor variations in column back-pressure during consecutive HPLC runs led to inter-assay variations in retention time. However, the column was calibrated for each experimental set by making a note of the retention time of 5PP-*myo*IP_4_, the product of *myo*-IP_5_ formed in the presence of IP6K1, and of 1PP-*myo*-IP_5_ and 5PP-*myo*-IP_5_, the products of *myo*-IP_6_ formed in the presence of VIP1-KD and IP6K1, respectively, as indicated in Figure 2. The conversion of IP_n_ substrate (10 nmoles and 5 nmoles respectively for IP6K1 and VIP1-KD reactions) to PPIP_n_ product (expressed in pmoles) was calculated based on the specific activity of ATP. All product peaks that were at least 5-fold above the baseline were counted. Data shown reflect the mean ± S.E.M. from four independent assays, and were analyzed using GraphPad PRISM.

### 3.4. Detection and Quantification of ATP Consumed and ADP Generated during the Kinase/ATPase Reaction

To monitor ATP consumption by purified recombinant 6×His-mIP6K1 and GST-VIP1-KD, we used the ATPlite luminescence detection system (Perkin Elmer) as per the manufacturer’s protocol. To rule out the possible co-purification of contaminating ATPases or phosphatases from the E. coli expression system, we used the generic ATPase inhibitor sodium orthovanadate (2 mM), or a mixture of phosphatase inhibitors (Phosphatase Inhibitor Cocktail 3, Sigma-Aldrich P0044, used at the recommended concentration). The reactions were set up with ~0.4 µM 6xHis-mIP6K1 or ~0.2 µM GST-VIP1-KD in 1X reaction buffer (20 mM Tris-Cl pH 7.4, 60 mM NaCl, 6 mM MgCl2, and 1 mM DTT) in a final volume of 25 µL. Bz-*scyllo*-IP_5_ (200 µM for IP6K1 and 100 µM for VIP1-KD) was used to stimulate the ATPase activity of the enzymes. The ATPase or phosphatase inhibitor, or a vehicle control, was added to the reaction for 15 min on ice, prior to the addition of ATP. The reactions were initiated by the addition of 40 µM Mg^2+^-ATP, incubated for 1 h at 37 °C and suitably diluted for measurement of ATP. A standard curve was plotted with increasing amount of ATP and the luminescence values were extrapolated to obtain the amount of ATP remaining after the reaction was terminated. Data shown reflect the mean ± range from two independent experiments and were analyzed using GraphPad PRISM.

The kinase/ATPase reactions for 6xHis-mIP6K1 and GST-VIP1-KD were set up in reaction buffer in a 10 µL or 12.5 µL reaction volume, respectively. The reactions were supplemented with 1 mM ATP and 200 µM IPn substrate for 6xHis-mIP6K1, and 40 µM ATP and 100 µM IPn substrate for GST-VIP1-KD, and ~1.2 µM purified enzyme. The final amount of ATP in the reaction was 10 nmoles and 500 pmoles, respectively, for IP6K1 and Vip1. Reactions were carried out for 18 h at 37 °C, cooled to 25 °C, and the amount of ADP generated was assayed using the ADP-GloTM kinase assay kit (Promega, Cat. # V6930), according to the manufacturer’s protocol. The ADP-GloTM kinase assay is a luminescence-based ADP detection method to measure the amount of ADP generated in the kinase reaction, and also reflects non-productive ATPase activity. After completion of the kinase/ATPase reaction, an equal volume of ADP-GloTM reagent was added and incubated at 25 °C for 30 min to terminate the reaction and to deplete the remaining ATP. The reaction mixture was transferred to a solid white 96-well plate, and double the volume of kinase detection reagent was added under dark, and incubated for 1 h to generate ATP from ADP, and measure the newly synthesized ATP using a luciferase/luciferin reaction. Luminescence was measured using a Multimode plate reader (EnSpire, Perkin Elmer, Waltham, MA, USA). The luminescence values were correlated with the percentage of ADP generated in the reaction by using an ATP-to-ADP standard curve. Appropriate volumes of ATP and ADP stock solutions were mixed to generate an ATP-to-ADP conversion standard curve. Separate standard curves were generated for IP6K1 and VIP1-KD—for IP6K1, 1 mM ATP and ADP solutions were mixed to obtain a range of ADP from 0% to 10% of ATP; and for Vip1, 40 µM ATP and ADP solutions were mixed to obtain a range of ADP from 0% to 40% of ATP. In every sample used for the standard curve, we included 1X reaction buffer and 100 µM or 200 µM IP6 respectively for VIP1-KD or IP6K1 assays, to account for any effects of buffer components or IPns on the luminescence reading. Standard curves were plotted and data was interpolated from three independent assays each for IP6K1 and VIP1-KD. Data shown reflect the mean ± S.E.M. from three independent assays, and were analyzed using GraphPad PRISM.

As shown in Figure 5, we observed significant basal ATPase activity without the addition of IPn substrate, especially in the case of VIP1-KD. The graphs shown in Figure 6 clearly depict the percentage ADP generated in the presence of IPn substrate, after subtracting the amount of ADP generated in the absence of substrate.

## Data Availability

The data presented in this study are available in the Supplementary Materials.

## Supplementary Materials

The following are available online, Section S1. PAGE analysis of PPIPn products; Section S2. Calibration of SAX HPLC for resolution of IPs and PPIPs; Section S3. Molecular Docking; Section S4. Effect of inhibitors on ATPase activity of IP kinases; Section S5. Spectral data. References [52,69,70,71,72,73,74,75] are cited in the Supplementary Materials.

## Sample Availability

Some of the samples are available in small quantities. This may be available for some time.
