# Supplementary material for: Novel Substrates for Kinases Involved in the Biosynthesis of Inositol Pyrophosphates and Their Enhancement of ATPase Activity of a Kinase
Source: Molecules. 2021 Jun 11;26(12):3601. doi: 10.3390/molecules26123601 (PMC8231259; doi:10.3390/molecules26123601)
Supplement: Supplementary file 1 [file molecules-26-03601-s001.zip › molecules-1230488-supplementary.pdf]

Supplementary Data

Novel Substrates for Kinases Involved in the Biosynthesis of Inositol Pyrophosphates and Their Enhancement of ATPase Activity of a Kinase

Raja Mohanrao [1], Ruth Manorama [2], Shubhra Ganguli [2,3], Mithun C. Madhusudhanan [1], Rashna Bhandari [2,*] and Kana M. Sureshan [1,*]

[1] School of Chemistry, Indian Institute of Science Education and Research Thiruvananthapuram, Kerala 695551, India; rajamohanrao1@gmail.com (R.M.), mithun716@iisertvm.ac.in (M.C.M.)
[2] Laboratory of Cell Signalling, Centre for DNA Fingerprinting and Diagnostics, Hyderabad 500039, India; manorama@cdfd.org.in (R.M.), shubhraganguli@cdfd.org.in (S.G.)
[3] Manipal Academy of Higher Education, Manipal, 576104, India.
* Correspondence: kms@iisertvm.ac.in (K.M.S.); rashna@cdfd.org.in (R.B.)

S. No	Table of contents	Page No.
1	PAGE analysis of PPIPn products	S2
2	Calibration of SAX HPLC for resolution of IPs and PPIPs	S3
3	Molecular Docking	S4-S13
4	Effect of inhibitors on ATPase activity of IP kinases	S14
5	Spectral data	S15-S50
6	References	S51

1. PAGE analysis of PPIPn products

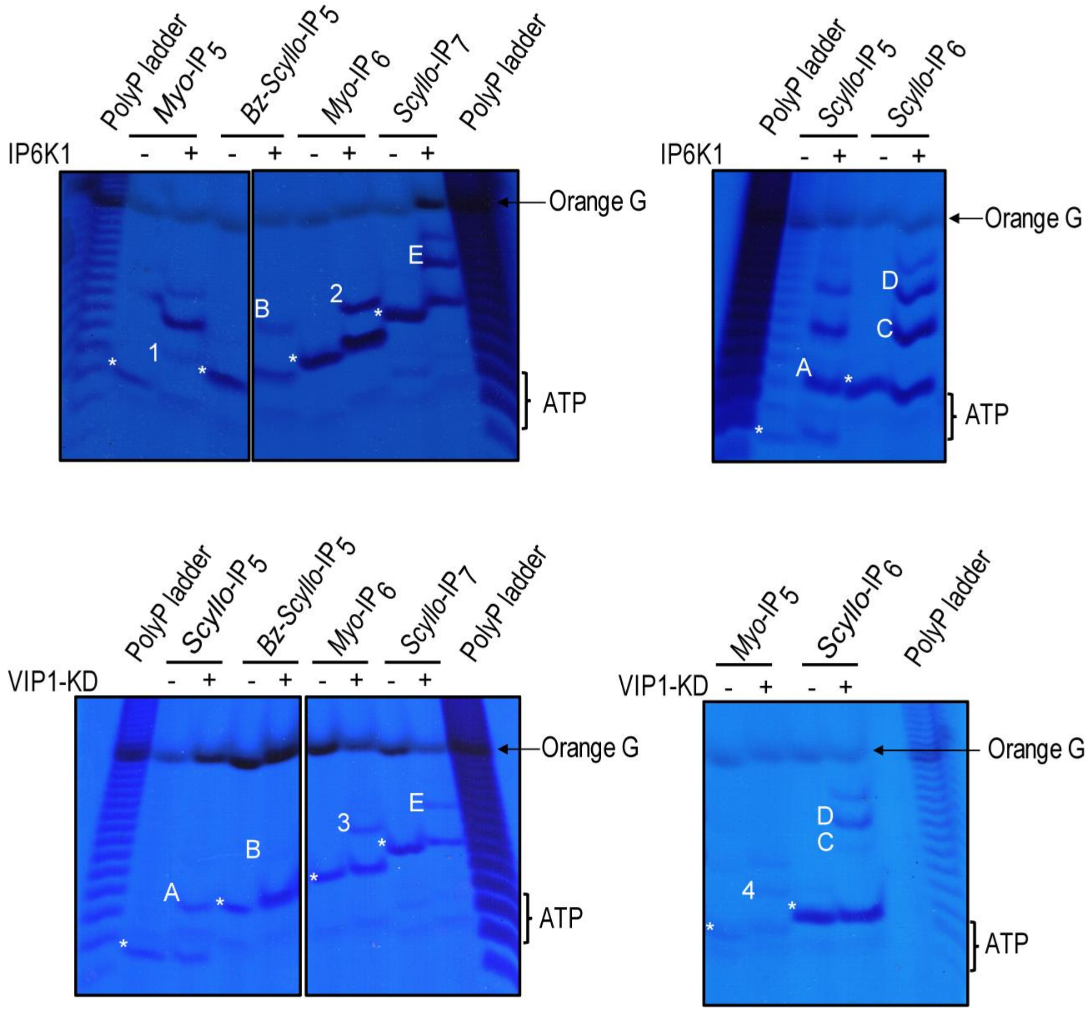

Figure S1. PAGE analysis followed by toluidine blue staining to detect PPIPn products formed by the catalysis of IP6K1 (top) and VIP1-KD (bottom) on various IPn substrates. Reactions were performed as described in Section 3.3. The unreacted IPn substrate (marked by an asterisk*) is loaded to the left of each post-reaction mixture. The migration of Orange G dye and inorganic polyphosphate (polyP) ladder were used as electrophoresis standards. The position of ATP, which has been shown to co-migrate with *myo*-IP_5 [1], is indicated. We observed that *scyllo*-IP_5 migrates faster than ATP. Based on the well characterized ability of IP6K1 and VIP1 to catalyse the addition of a β-phosphate on the 5- and 1-position on *myo*-inositol, the products of *myo*-IP_5 and *myo*-IP_6 marked on the gel are: 1. 5PP-*myo*-IP_4, 2. 5PP-*myo*-IP_5, 3. 1PP-*myo*-IP_5, and 4. 1PP-*myo*-IP_4. Each consecutive band of IPn or PPIPn in the same lane differs by the presence of a single phosphate moiety [1]. This, combined with the demonstrated ability of IP6K1 and VIP1 to catalyse the addition of a β-phosphate onto an α- phosphate on the inositol ring, allows us to propose the structures of the *scyllo*-PPIPn products as follows: A. PP-*scyllo*-IP_4, B. PP-Bz-*scyllo*-IP_4, C. PP-*scyllo*-IP_5, D. $[PP]_2$-scyllo-IP_4 / PPP-scyllo-IP_5, E. $[PP]_2$-scyllo-IP_4 / PPP-scyllo-IP_5. This annotation corresponds to the labelling of peaks in the chromatograms shown in Figure 2. The scanned gels were subjected to ‘brightness and contrast’ adjustment to improve visualisation. A narrow gap marks the removal of a lane from a single gel for better visualisation.

2. Calibration of SAX HPLC for resolution of IPs and PPIPs

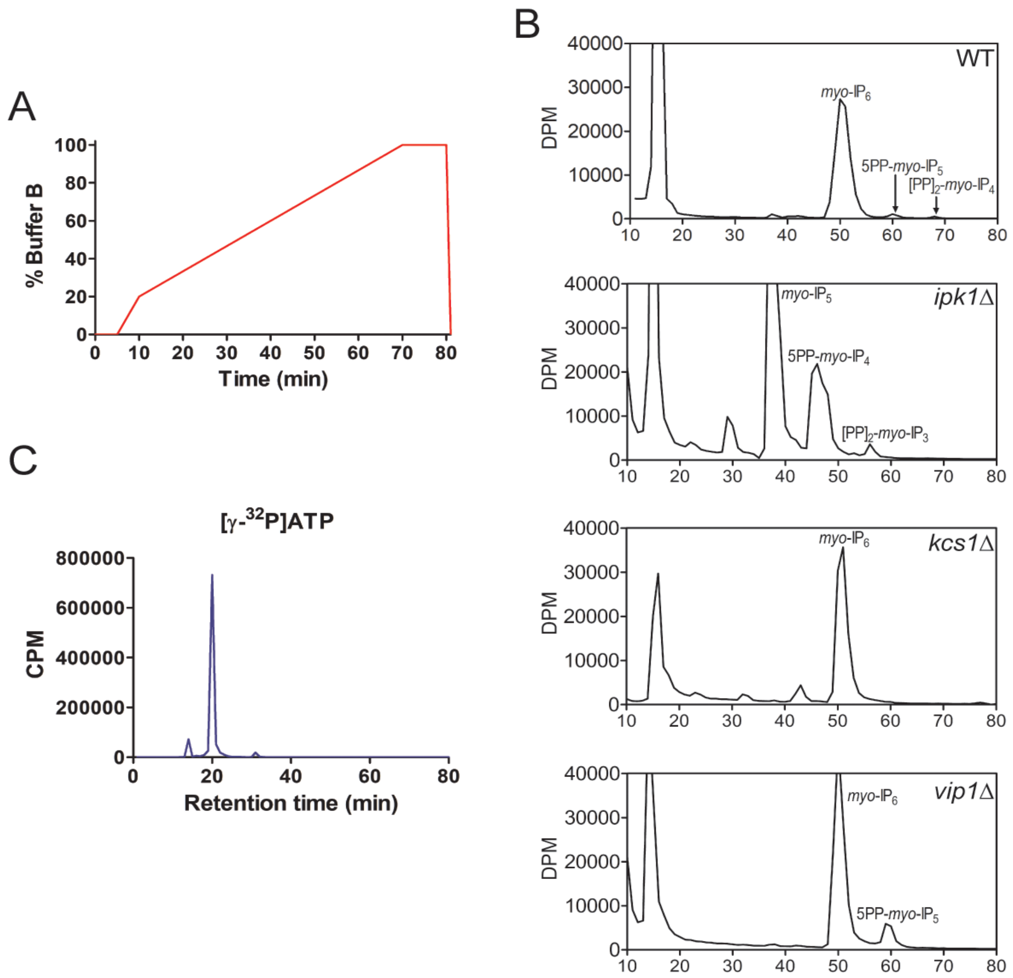

Figure S2. **(A)** Graphical representation of SAX HPLC elution gradient profile described in Section 3.3. **(B)** SAX-HPLC analysis of extracts from [^{3}H]inositol labeled *S. cerevisiae* BY4741 wild type (WT) strain, and deletion mutants lacking the enzymes (i) IPK1 responsible for the conversion of IP_5 to IP_6 (*ipk1*Δ); (ii) KCS1, the *S. cerevisiae* IP6 kinase responsible for the conversion of IP_6 to 5PP-IP_5 (*kcs1*Δ); and (iii) VIP1 (*vip1*Δ). The IPs in the elution profiles are annotated as reported earlier [2,3] Peaks corresponding to *myo*-IP_6, 5PP-*myo*-IP_5 and 1,5[PP]$_2$-*myo*-IP_4 are marked in the WT strain. In *ipk1*Δ yeast, peaks corresponding to *myo*-IP_6 and its PPIP products disappeared, and *myo*-IP_5 and PP-*myo*-IP_4 peaks became abundant. 5PP-*myo*-IP_5 and 1,5[PP]$_2$-*myo*-IP_4 are absent in *kcs1*Δ with a corresponding increase in the *myo*-IP_6 peak. In *vip1*Δ yeast, there was an increase in *myo*-IP_6 and 5PP-*myo*-IP_5, with the disappearance of 1,5[PP]$_2$-*myo*-IP_4. These elution profiles of well characterised yeast strains were used to mark the retention times of the PPIPn products of *myo*-IP_5, myo-IP_6 shown in Figure 2. We observe lower retention times for *scyllo*-PPIPs compared with their myo-counterparts (see Figure 2). **(C)** Chromatogram showing elution profile of radiolabeled ATP in the negative control reaction containing *myo*-IP_6 and [γ^{32}P]ATP incubated in the presence of BSA instead of enzyme.

3. Molecular Docking

Molecular docking studies were carried out by treating *myo*-IP6 and *scyllo*-IP6 as ligands using CCDC GOLD package using GOLDScore as the scoring function [4]. The X-Ray crystal structure of IP6 from the PDB files of PPIPK2 and *Eh*IP6KA complex (PDB code: 3T9C, 4O4F) was used as the initial input, which was modified to *scyllo*-IP6 using Gaussview 6 [5]. Both *myo* and *scyllo* IP6 structures were then energy minimised using MM94 forcefield in Avogadro [6] and thus further utilised for docking experiments. All except three water molecules were removed from both peptide domain, along with the removal of AMPPNP(PPIP5K2) and ATP and PO4 $^{3-}$ (*Eh*IP6KA) and Mg^{2+} ions. In GOLD, the binding site was defined as a sphere of 6 Å radius and water molecules were included in the docking protocol (30,35,462 for PPIP5K2 and 587,620,667 for *Eh*IP6KA) these water molecules were identified near the axial site of IP6 and was toggled on and off, thus necessitating to evaluate their possible role in forming Water Bridges at the axial position. Each ligand was docked 100 times (100 GA runs) using genetic algorithm settings for very flexible ligands. The results of GOLDScore function were observed to be unusually high but such an observation is not new and the probable explanation being the tendency of GOLDScore to reward heavily for hydrogen bonding involving charged phosphates, [7] even though these scoring functions are optimised for accurate pose prediction, they do might have an influence w.r.t. binding affinities of the peptide ligand system. The fitness scores for GOLDScore is given in Figure S2. The binding modes of *myo*-IP6 and *scyllo*-IP6 with the highest docking score against the above-mentioned kinases were obtained and their interactions visualised using Protein Ligand Profiler (PLIP) [8]. Tables S1-S4 give further details on the possible interactions observed at the protein ligand interface.

Table S1. Results of Docking *myo*-IP6 on PPIP5K2- GOLDScore results

Hydrogen Bonds

Index	Residue	AA	Distance H-A	Distance D-A	Donor Angle	Donor Atom	Acceptor Atom
1	250A	TYR	2.47	3.38	151.91	3481 [O3]	5421 [O3]
2	250A	TYR	2.73	3.38	125.44	5421 [O3]	3481 [O3]
3	250A	TYR	2.64	3.35	129.62	5400 [O3]	3481 [O3]
4	250A	TYR	3.03	3.66	123.76	5399 [O3]	3481 [O3]
5	262A	ARG	2.95	3.61	123.43	3657 [Ng+]	5412 [O3]
6	262A	ARG	2.46	2.89	104.56	3654 [N3]	5406 [O3]

Index	Residue	AA	Distance H-A	Distance D-A	Donor Angle	Donor Atom	Acceptor Atom
7	281A	ARG	2.61	3.14	111.78	3974 [N3]	5399 [O3]
8	281A	ARG	3.00	3.67	124.73	3972 [N3]	5397 [O2]
9	321A	ASP	2.39	2.73	101.60	4642 [O3]	5415 [O3]
10	323A	ASN	3.06	3.43	102.65	4672 [Nam]	5416 [O3]
11	323A	ASN	2.94	3.58	124.81	5420 [O3]	4671 [O2]
12	326A	SER	3.26	3.97	131.26	5425 [O3]	4717 [O3]
13	326A	SER	2.30	3.28	171.47	4717 [O3]	5424 [O2]

*Distance in angstrom (Å), angles in degrees

Water Bridges

Index	Residue	AA	Dist. A-W	Dist. D-W	Donor Angle	Water Angle	Donor Atom	Acceptor Atom	Water Atom
1	54A	LYS	2.74	4.05	103.98	88.39	199 [N3+]	5425 [O3]	5444
2	54A	LYS	4.04	4.05	112.41	137.19	199 [N3+]	5426 [O3]	5444

*Distance in angstrom (Å), angles in degrees

Salt Bridges

Index	Residue	AA	Distance	Ligand Group	Ligand Atoms
1	54A	LYS	4.62	Phosphate	5423, 5423, 5422, 5425, 5426, 5424

Index	Residue	AA	Distance	Ligand Group	Ligand Atoms
2	194A	HIS	4.65	Phosphate	5413, 5413, 5412, 5414, 5415, 5416
3	213A	ARG	4.20	Phosphate	5413, 5413, 5412, 5414, 5415, 5416
4	213A	ARG	4.48	Phosphate	5408, 5408, 5407, 5409, 5410, 5411
5	214A	LYS	3.36	Phosphate	5403, 5403, 5402, 5404, 5405, 5406
6	248A	LYS	4.58	Phosphate	5413, 5413, 5412, 5414, 5415, 5416
7	248A	LYS	3.66	Phosphate	5418, 5418, 5417, 5419, 5420, 5421
8	248A	LYS	5.15	Phosphate	5398, 5398, 5397, 5401, 5399, 5400
9	262A	ARG	4.91	Phosphate	5408, 5408, 5407, 5409, 5410, 5411
10	262A	ARG	5.31	Phosphate	5398, 5398, 5397, 5401, 5399, 5400
11	329A	LYS	3.37	Phosphate	5423, 5423, 5422, 5425, 5426, 5424
12	329A	LYS	5.22	Phosphate	5418, 5418, 5417, 5419, 5420, 5421
13	329A	LYS	3.58	Phosphate	5398, 5398, 5397, 5401, 5399, 5400

*Distance in angstrom (Å), angles in degrees

Table S2. Results of Docking *scyllo*-IP6 on PPIP5K2- GOLDScore results Hydrogen Bonds

Index	Residue	AA	Distance H-A	Distance D-A	Donor Angle	Donor Atom	Acceptor Atom
1	213A	ARG	3.28	3.95	125.23	2850 [Ng+]	5421 [O3]
2	214A	LYS	1.74	2.63	142.89	2864 [Nam]	5411 [O3]
3	262A	ARG	3.51	3.93	106.86	3657 [Ng+]	5415 [O3]
4	262A	ARG	2.69	3.60	149.59	3654 [N3]	5428 [O3]
5	262A	ARG	2.44	3.45	170.69	3655 [Ng+]	5409 [O2]
6	262A	ARG	2.87	3.68	153.57	3656 [N3]	5430 [O2]
7	262A	ARG	1.85	2.81	174.20	5431 [O3]	3656 [N3]
8	281A	ARG	3.02	4.02	168.84	3974 [N3]	5397 [O3]
9	281A	ARG	2.79	3.60	137.24	3972 [N3]	5431 [O3]
10	281A	ARG	2.10	2.90	138.91	5400 [O3]	3974 [N3]
11	321A	ASP	2.85	3.29	109.97	4642 [O3]	5415 [O3]
12	323A	ASN	2.94	3.44	111.57	4672 [Nam]	5414 [O2]
13	323A	ASN	2.43	3.31	150.35	5415 [O3]	4671 [O2]
14	326A	SER	1.86	2.83	164.96	4717 [O3]	5419 [O2]

*Distance in angstrom (Å), angles in degrees

Salt Bridges

Index	Residue	AA	Distance	Ligand Group	Ligand Atoms
1	194A	HIS	4.68	Phosphate	5413, 5413, 5412, 5414, 5415, 5416
2	213A	ARG	4.02	Phosphate	5413, 5413, 5412, 5414, 5415, 5416
3	213A	ARG	4.53	Phosphate	5408, 5408, 5407, 5409, 5410, 5411
4	214A	LYS	4.04	Phosphate	5398, 5398, 5397, 5401, 5399, 5400
5	214A	LYS	4.31	Phosphate	5403, 5403, 5402, 5404, 5405, 5406
6	248A	LYS	4.04	Phosphate	5418, 5418, 5417, 5419, 5420, 5421
7	248A	LYS	4.50	Phosphate	5413, 5413, 5412, 5414, 5415, 5416
8	248A	LYS	5.09	Phosphate	5429, 5429, 5428, 5430, 5431, 5432
9	262A	ARG	5.47	Phosphate	5398, 5398, 5397, 5401, 5399, 5400
10	262A	ARG	4.26	Phosphate	5403, 5403, 5402, 5404, 5405, 5406
11	329A	LYS	4.99	Phosphate	5418, 5418, 5417, 5419, 5420, 5421
12	329A	LYS	3.34	Phosphate	5429, 5429, 5428, 5430, 5431, 5432

*Distance in angstrom (Å), angles in degrees

Table S3. Results of Docking *myo*-IP6 on *Eh*IP6KA- GOLDScore results Hydrogen Bonds

Index	Residue	AA	Distance H-A	Distance D-A	Donor Angle	Donor Atom	Acceptor Atom
1	23A	GLY	2.35	3.37	175.70	1 [N3]	4190 [O2]
2	106A	THR	3.11	3.91	140.12	4171 [O3]	1375 [O3]
3	106A	THR	3.56	4.02	110.34	1375 [O3]	4170 [O3]
4	106A	THR	2.42	3.27	145.55	4170 [O3]	1373 [O2]
5	134A	ARG	3.16	3.86	127.17	1814 [Ng+]	4168 [O2]
6	136A	SER	2.23	3.16	154.16	1853 [O3]	4181 [O3]
7	136A	SER	2.27	3.16	151.54	4181 [O3]	1853 [O3]
8	136A	SER	2.87	3.50	123.29	4187 [O3]	1853 [O3]
9	152A	ARG	3.22	3.60	104.06	2109 [Ng+]	4178 [O3]
10	207A	SER	2.36	3.34	169.24	3100 [O3]	4170 [O3]

*Distance in angstrom (Å), angles in degrees

Salt Bridges

Index	Residue	AA	Distance	Ligand Group	Ligand Atoms
1	101A	LYS	3.50	Phosphate	4179, 4179, 4178, 4180, 4181, 4182

Index	Residue	AA	Distance	Ligand Group	Ligand Atoms
2	101A	LYS	3.70	Phosphate	4169, 4169, 4168, 4171, 4172, 4170
3	101A	LYS	4.42	Phosphate	4184, 4184, 4183, 4187, 4185, 4186
4	115A	LYS	4.43	Phosphate	4194, 4194, 4193, 4195, 4196, 4197
5	118A	LYS	3.84	Phosphate	4174, 4174, 4173, 4175, 4176, 4177
6	118A	LYS	4.52	Phosphate	4194, 4194, 4193, 4195, 4196, 4197
7	119A	ARG	4.71	Phosphate	4174, 4174, 4173, 4175, 4176, 4177
8	119A	ARG	3.62	Phosphate	4169, 4169, 4168, 4171, 4172, 4170
9	119A	ARG	4.18	Phosphate	4194, 4194, 4193, 4195, 4196, 4197
10	134A	ARG	4.14	Phosphate	4179, 4179, 4178, 4180, 4181, 4182
11	152A	ARG	3.88	Phosphate	4184, 4184, 4183, 4187, 4185, 4186

*Distance in angstrom (Å), angles in degrees

Table S4. Results of Docking *scyllo*-IP6 on EhIP6KA- GOLDScore results Hydrogen Bonds

Index	Residue	AA	Distance H-A	Distance D-A	Donor Angle	Donor Atom	Acceptor Atom
1	106A	THR	3.24	3.65	107.45	4187 [O3]	1375 [O3]
2	134A	ARG	2.71	3.41	125.76	1814 [Ng+]	4187 [O3]
3	136A	SER	2.01	2.98	162.72	1853 [O3]	4202 [O3]
4	136A	SER	2.02	2.98	168.67	4202 [O3]	1853 [O3]
5	152A	ARG	3.57	4.07	114.77	4192 [O3]	2105 [O2]
6	152A	ARG	3.32	3.68	102.71	2109 [Ng+]	4199 [O3]

*Distance in angstrom (Å), angles in degrees

Salt Bridges

Index	Residue	AA	Distance	Ligand Group	Ligand Atoms
1	101A	LYS	3.81	Phosphate	4200, 4200, 4199, 4203, 4201, 4202
2	101A	LYS	4.11	Phosphate	4169, 4169, 4168, 4171, 4172, 4170
3	115A	LYS	4.70	Phosphate	4179, 4179, 4178, 4180, 4181, 4182
4	118A	LYS	5.02	Phosphate	4189, 4189, 4188, 4190, 4191, 4192
5	118A	LYS	4.40	Phosphate	4184, 4184, 4183, 4187, 4185, 4186

Index	Residue	AA	Distance	Ligand Group	Ligand Atoms
6	118A	LYS	3.94	Phosphate	4179, 4179, 4178, 4180, 4181, 4182
7	119A	ARG	4.00	Phosphate	4184, 4184, 4183, 4187, 4185, 4186
8	119A	ARG	3.81	Phosphate	4179, 4179, 4178, 4180, 4181, 4182
9	134A	ARG	4.10	Phosphate	4200, 4200, 4199, 4203, 4201, 4202
10	152A	ARG	4.11	Phosphate	4169, 4169, 4168, 4171, 4172, 4170
11	152A	ARG	4.32	Phosphate	4174, 4174, 4173, 4175, 4176, 4177

*Distance in angstrom (Å), angles in degrees

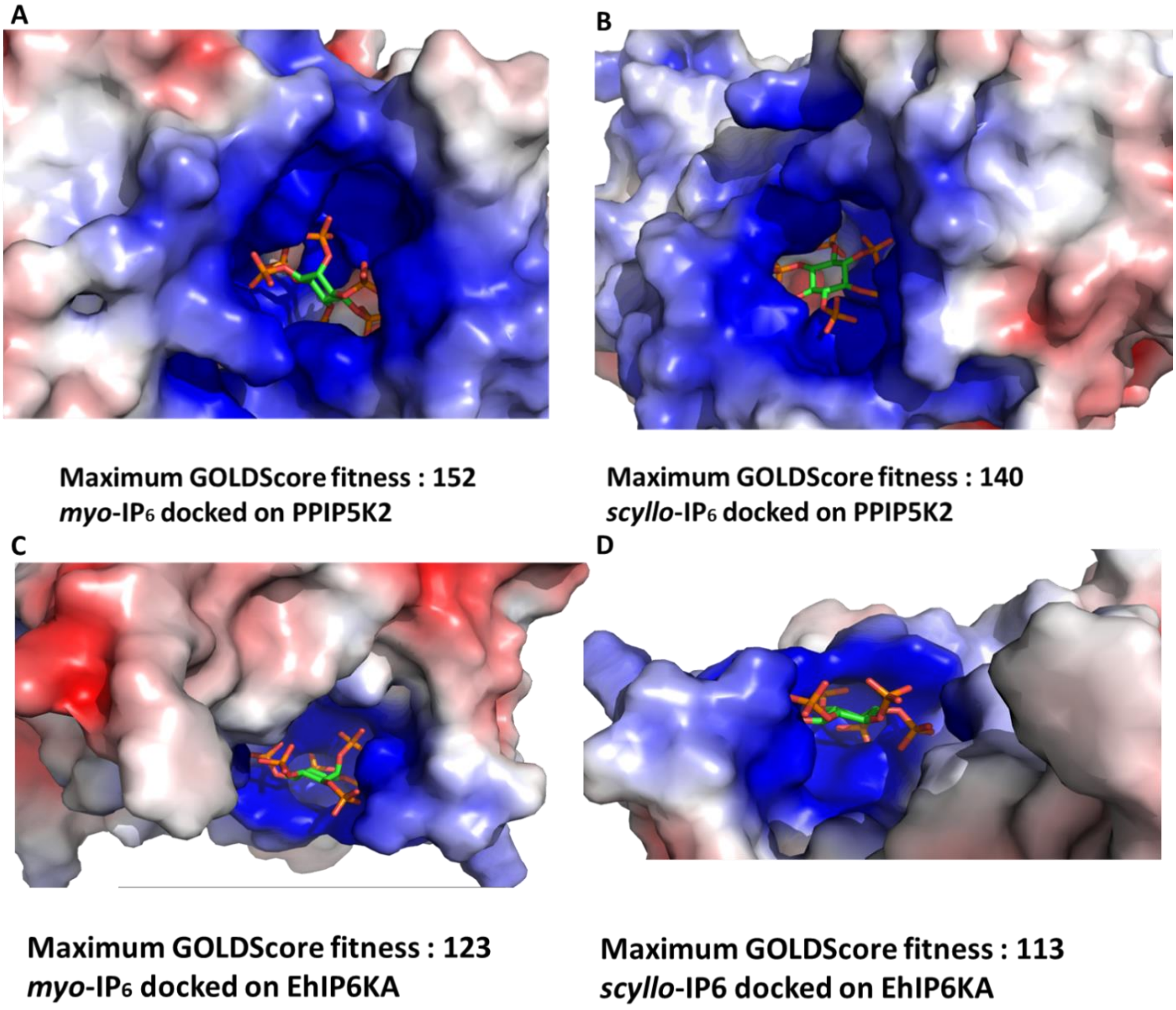

Figure S3. The highest scoring poses of *myo*- and *scyllo*-IP6 docked at the binding site of PPIP5K2 and *Eh*IP6KA. The protein is shown as a solvent-accessible surface, with the electrostatic potential maps being generated using APBS Tools within PyMOL.

4. **Effect of inhibitors on ATPase activity of IP kinases**

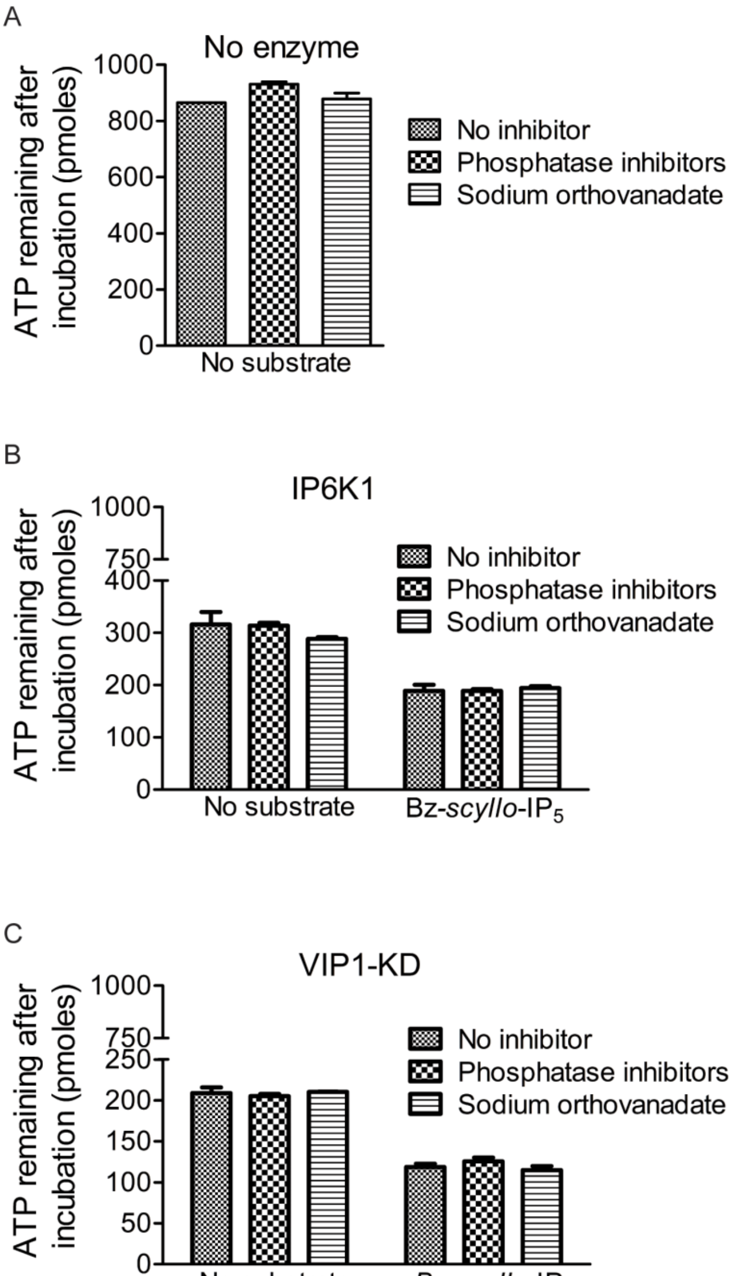

Figure S4: Effect of inhibitors on ATPase activity of IP kinases. Consumption of ATP by 6xHis-mIP6K1 and GST-VIP1-KD was monitored by a luminescence-based assay to measure ATP in the reaction mix after incubation with enzyme. (A) There was no effect of a phosphatase inhibitor cocktail or the generic ATPase inhibitor sodium orthovanadate on the detection of ATP by the assay. (B, C) ATP was consumed in the presence of IP6K1 (compare B with A) and VIP1-KD (compare C with A), and in both cases, ATP consumption was increased in the presence of Bz-*scyllo*-IP_5 (also see Figure 5). The presence of phosphatase inhibitors or sodium orthovanadate had no effect on basal or Bz-*scyllo*-IP_5 -stimulated ATP consumption by IP6K1 or VIP1, indicating the absence of any contaminating phosphatase or ATPase in the purified enzyme preparations. The data show mean ± range from two independent experiments.

5. **Spectral data**

^{1}H NMR of compound 4

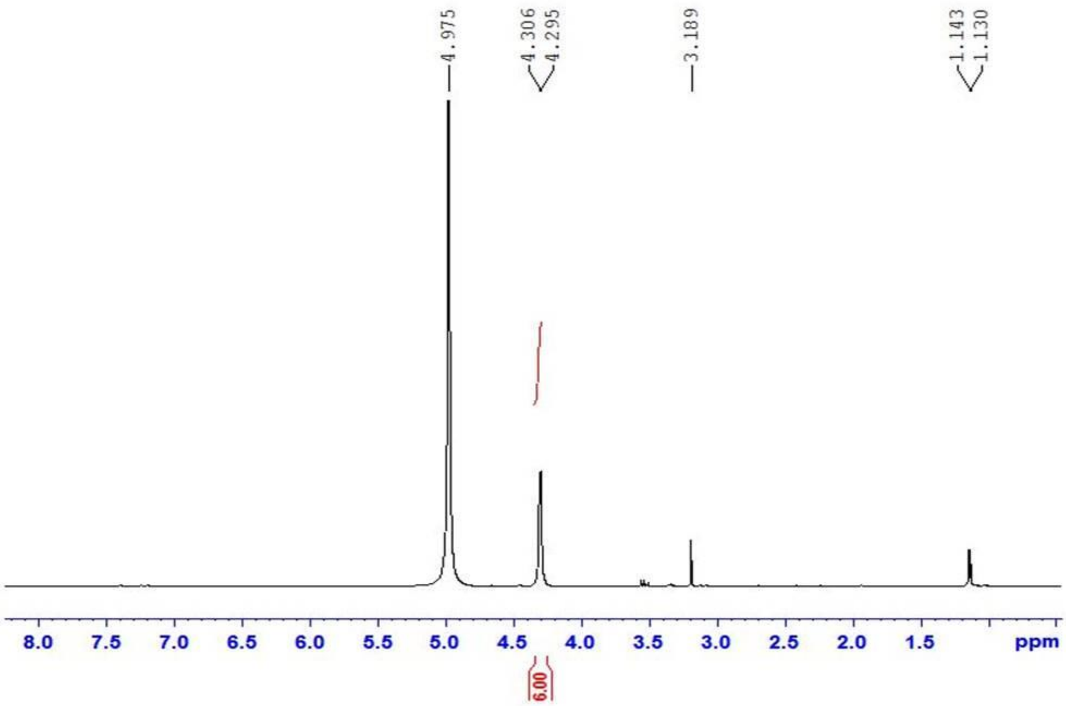

^{31}P NMR of compound 4

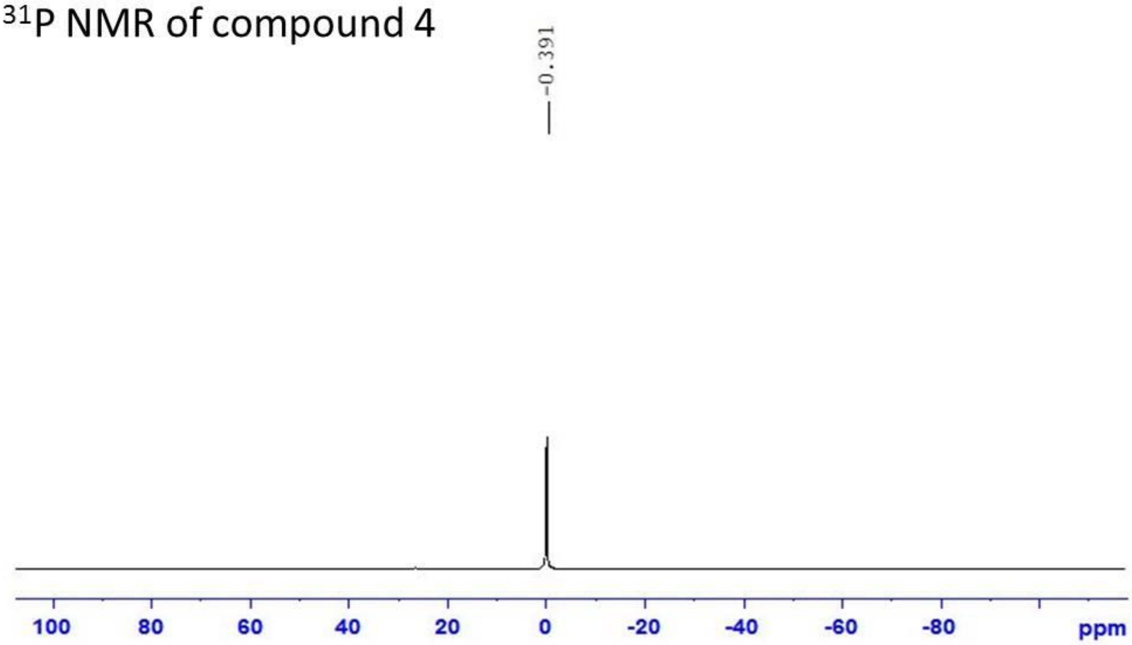

^{1}H NMR of compound 5

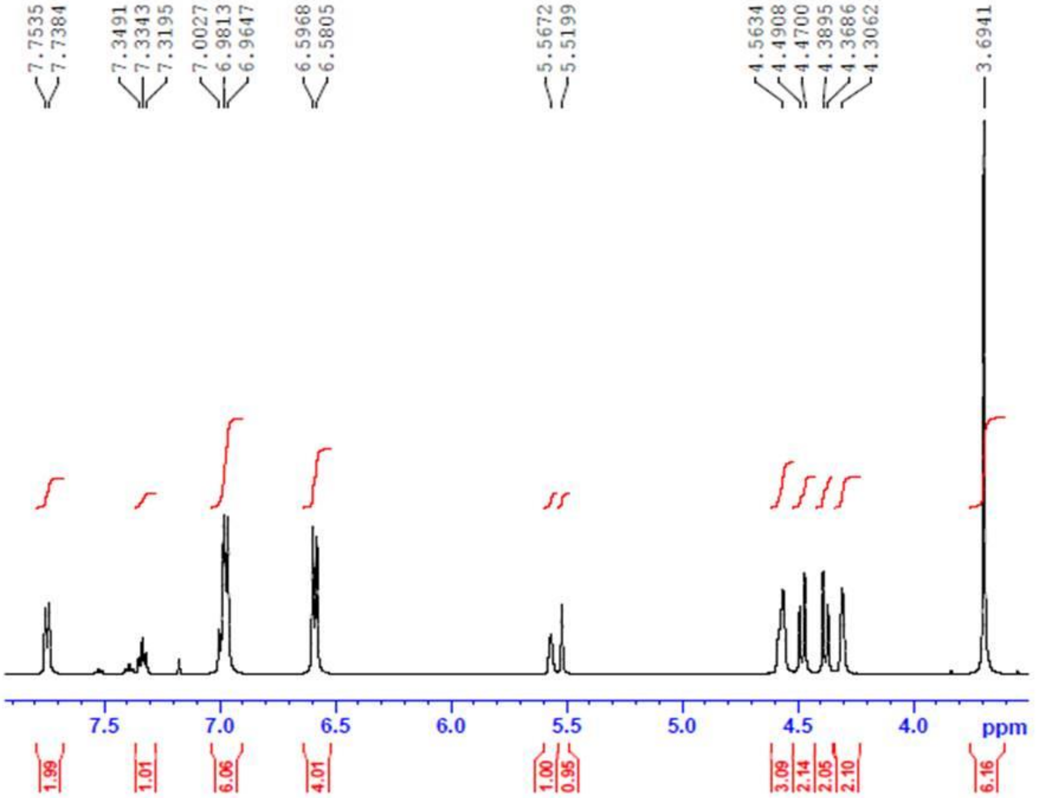

Cosy of compound 5

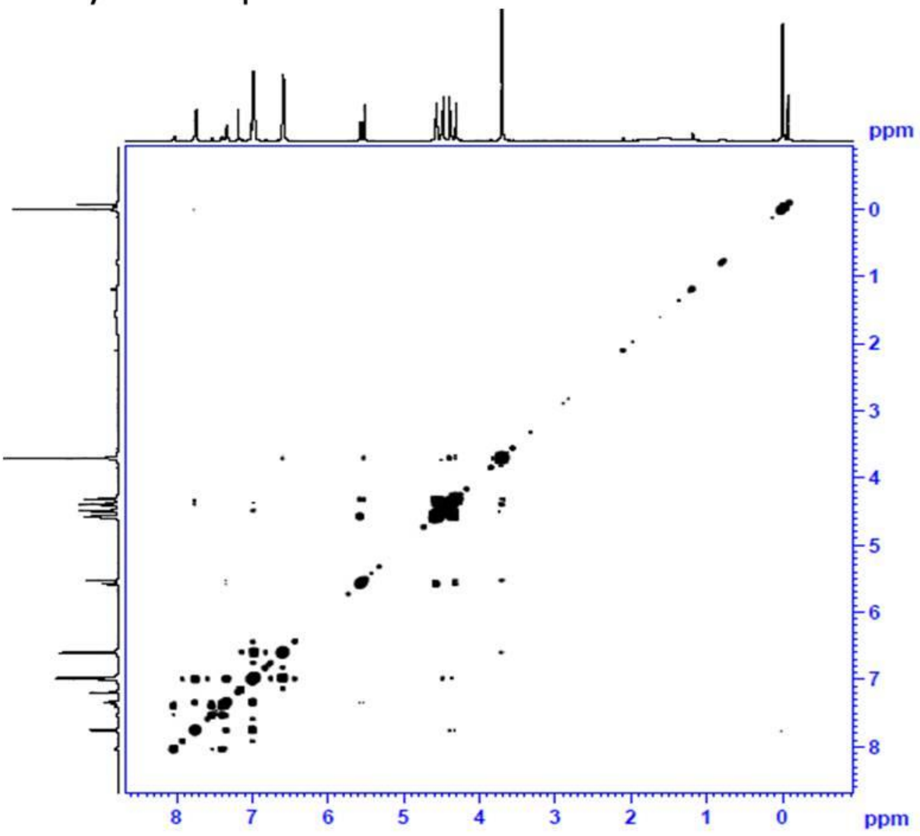

DEPT of compound 5

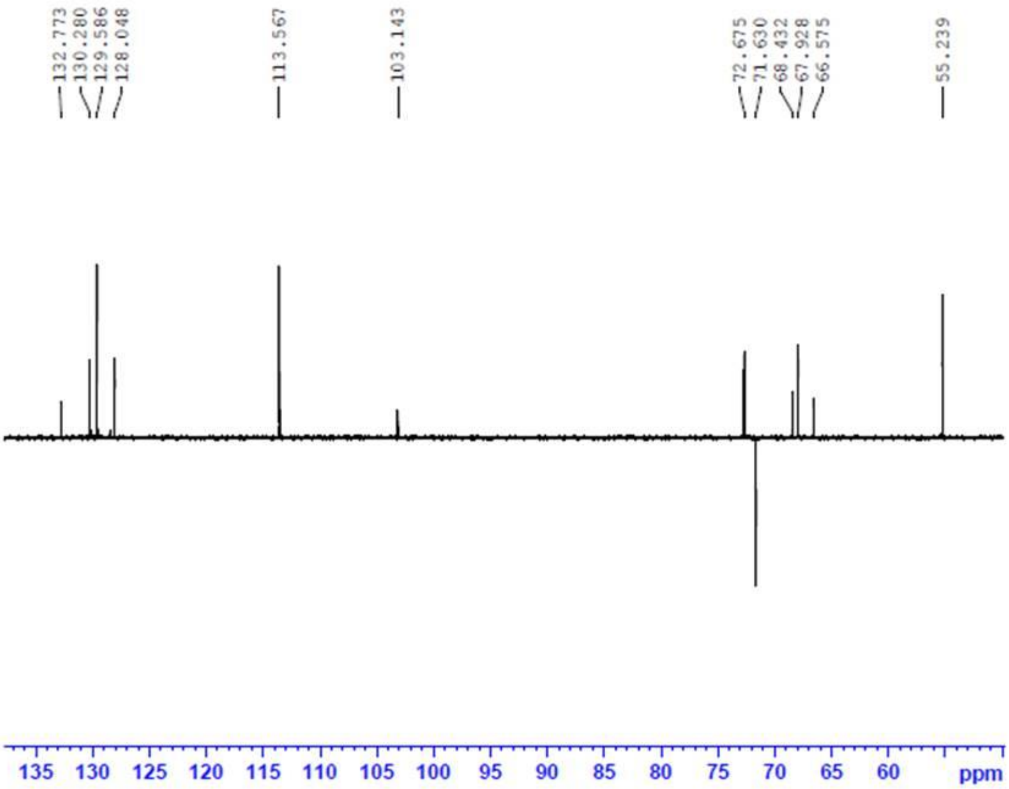

^{13}C of compound 5

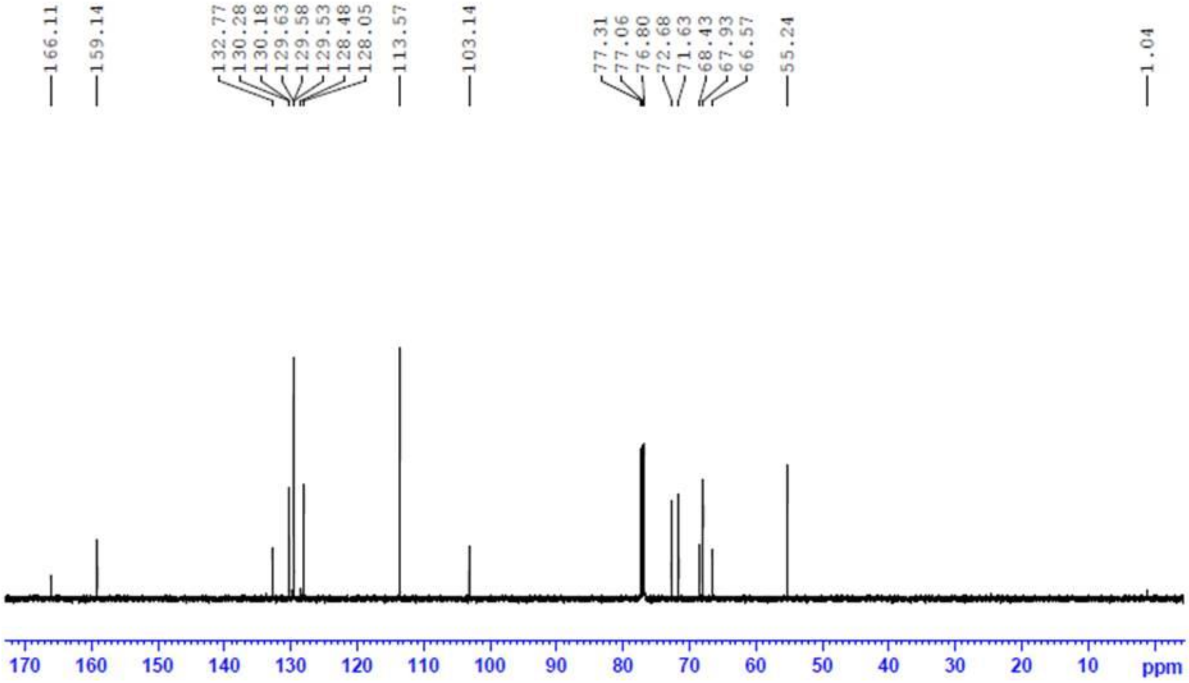

HMQC of compound 5

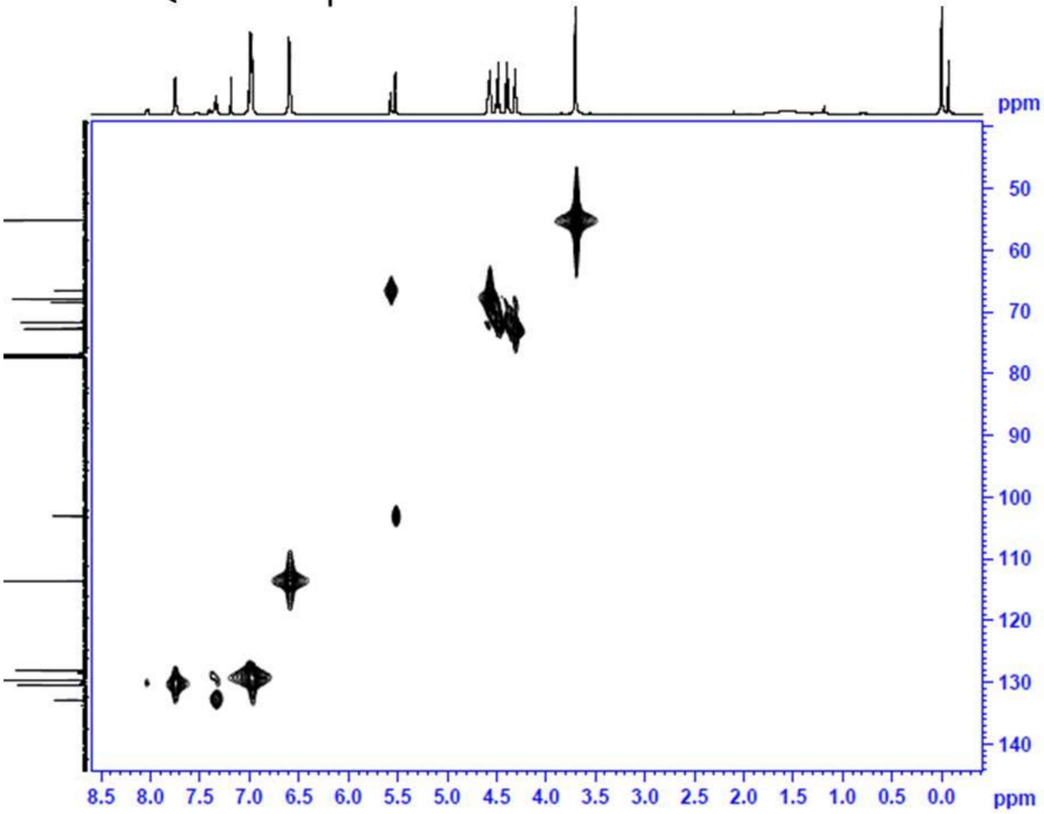

^{1}H NMR of compound 6

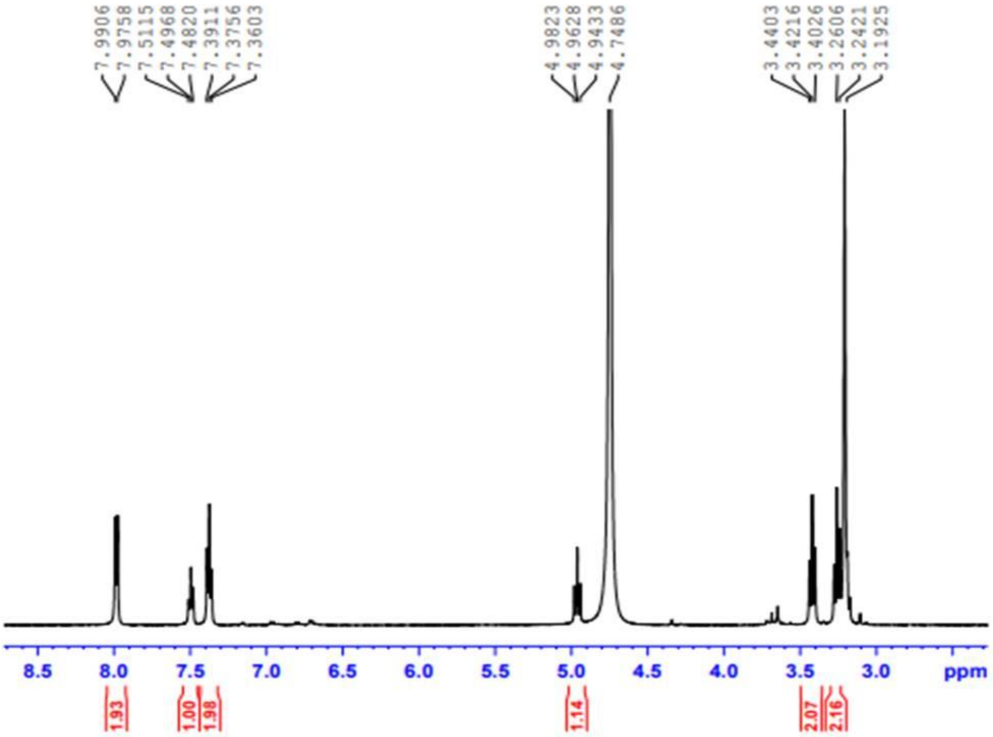

^1H NMR of compound 7

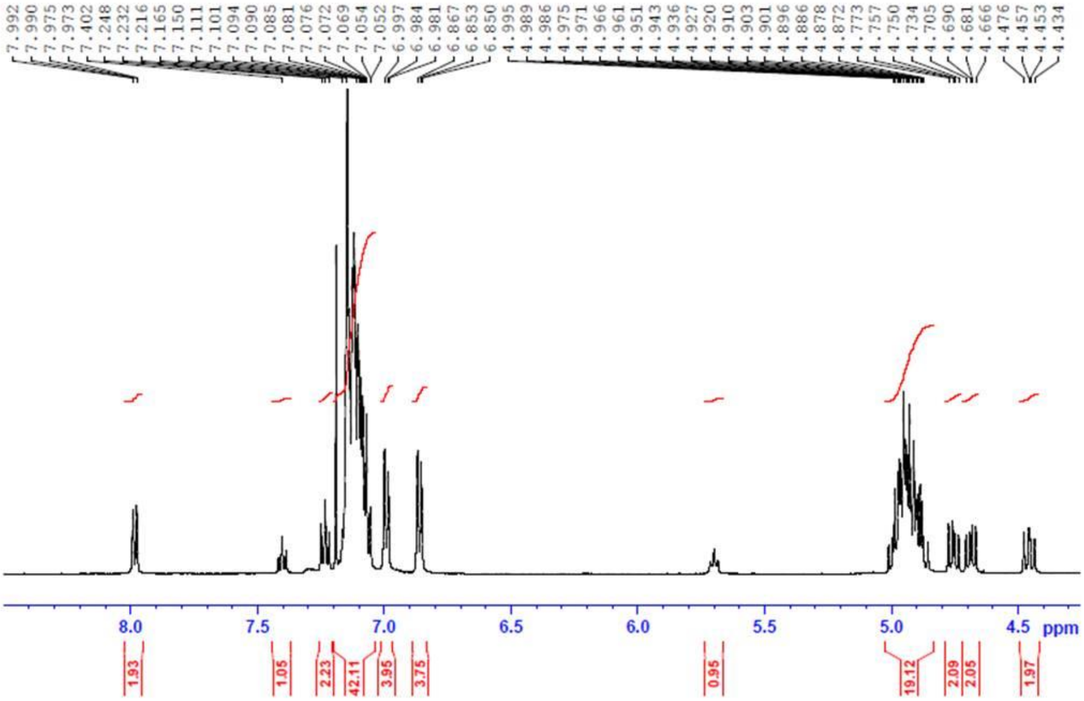

COSY of compound 7

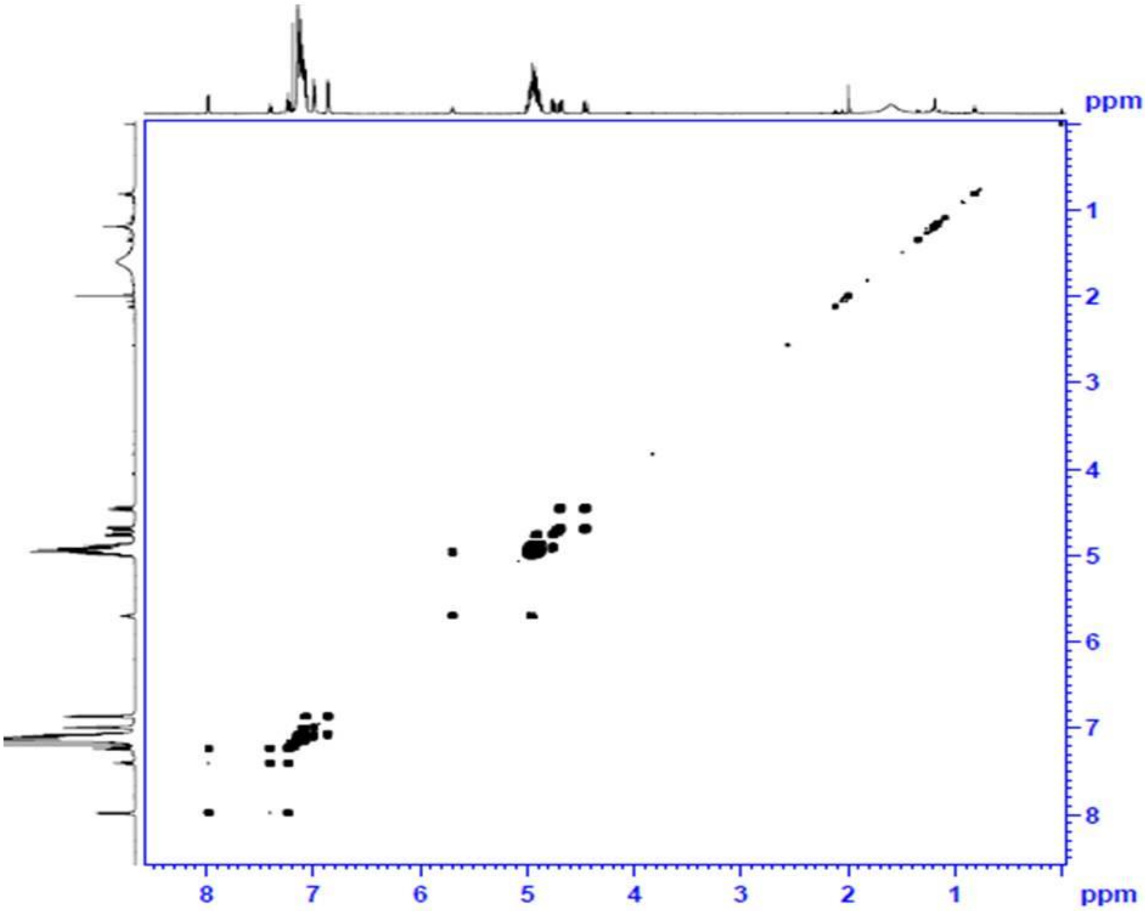

DEPT of compound 7

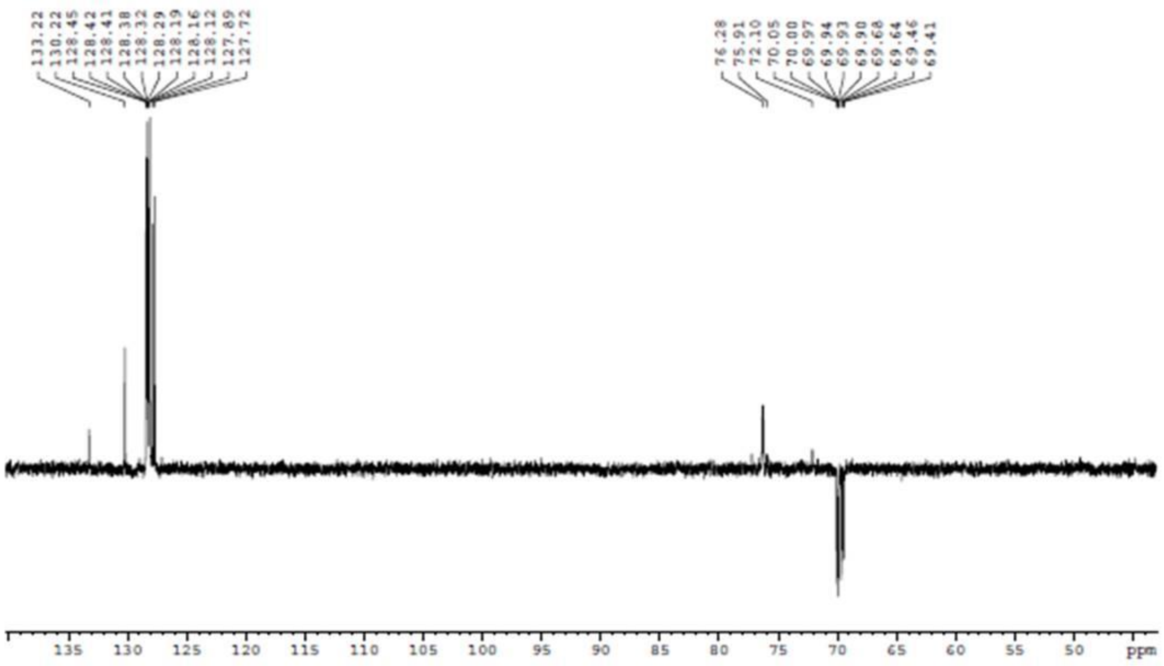

^{13}C of compound 7

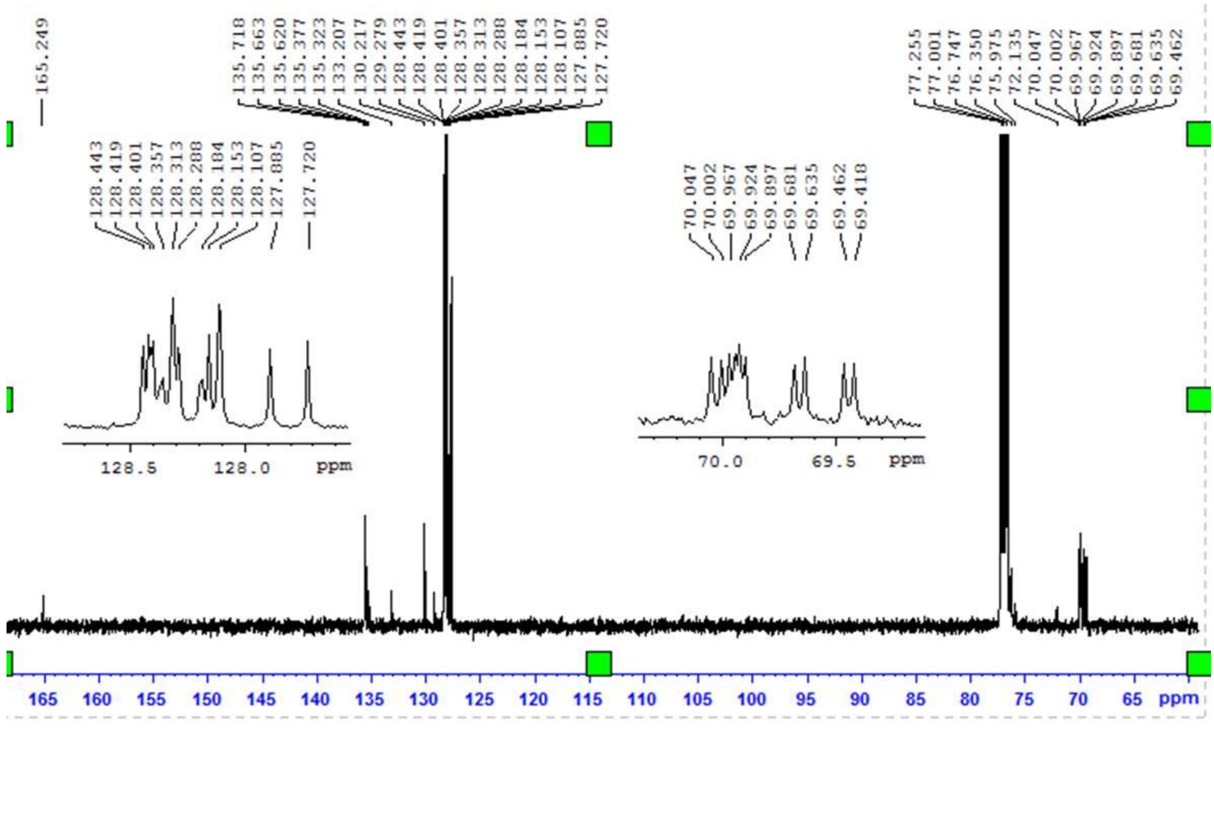

HMQC of compound 7

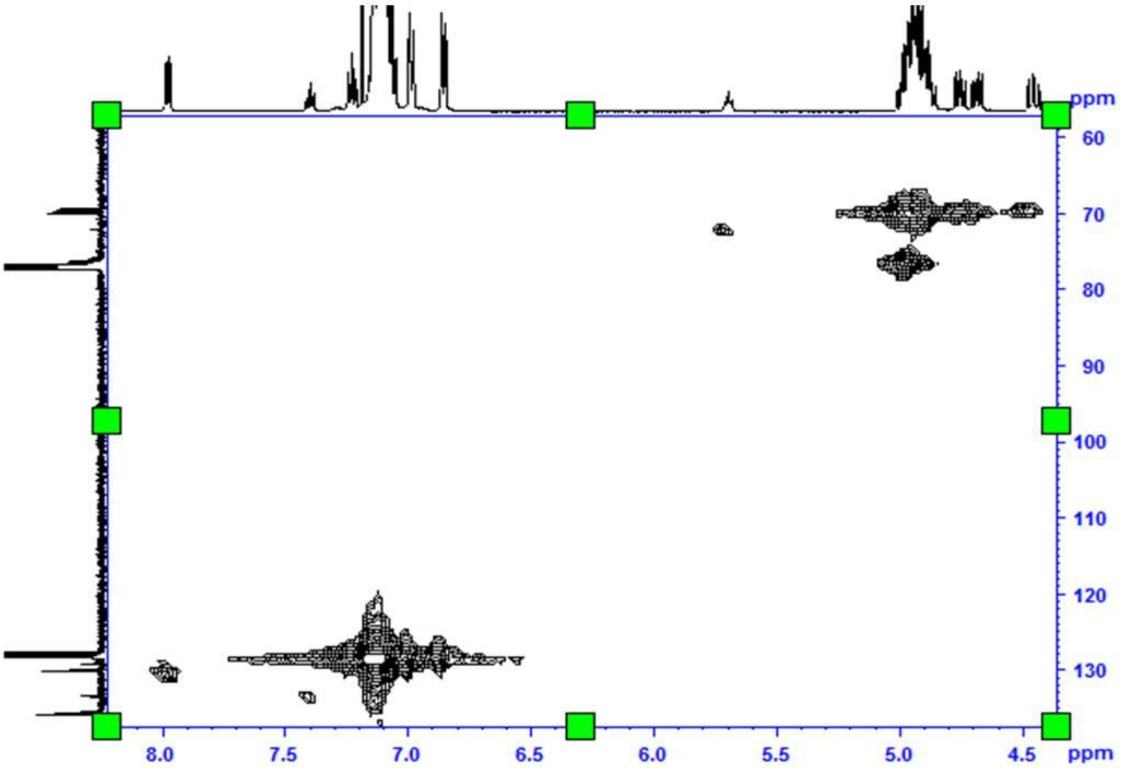

^1H NMR of compound 8 (CD_3OD)

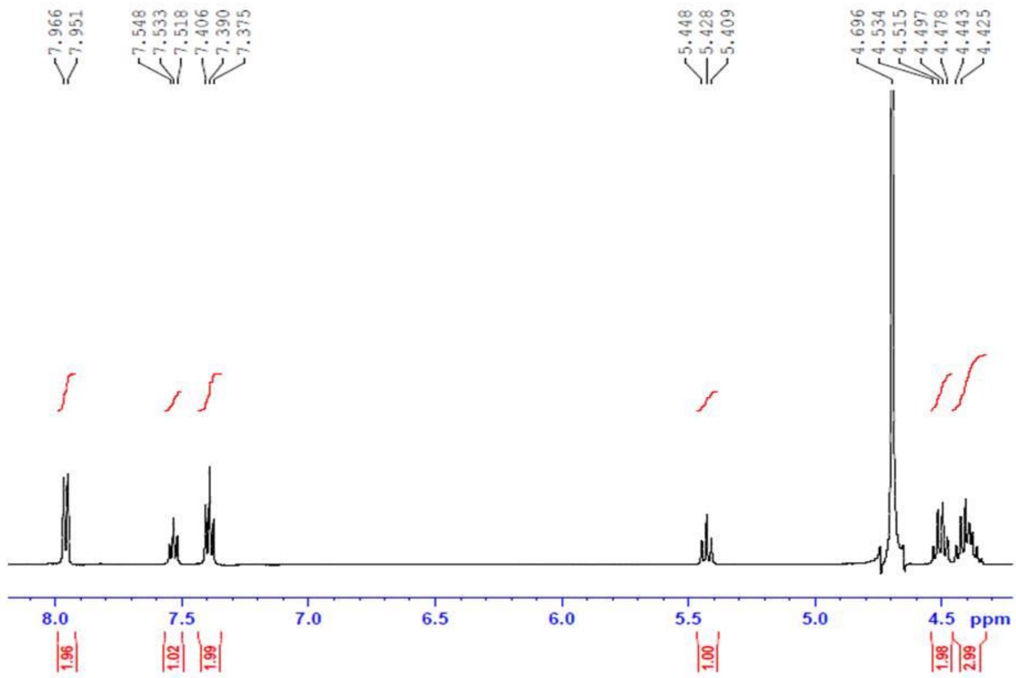

COSY of compound 8

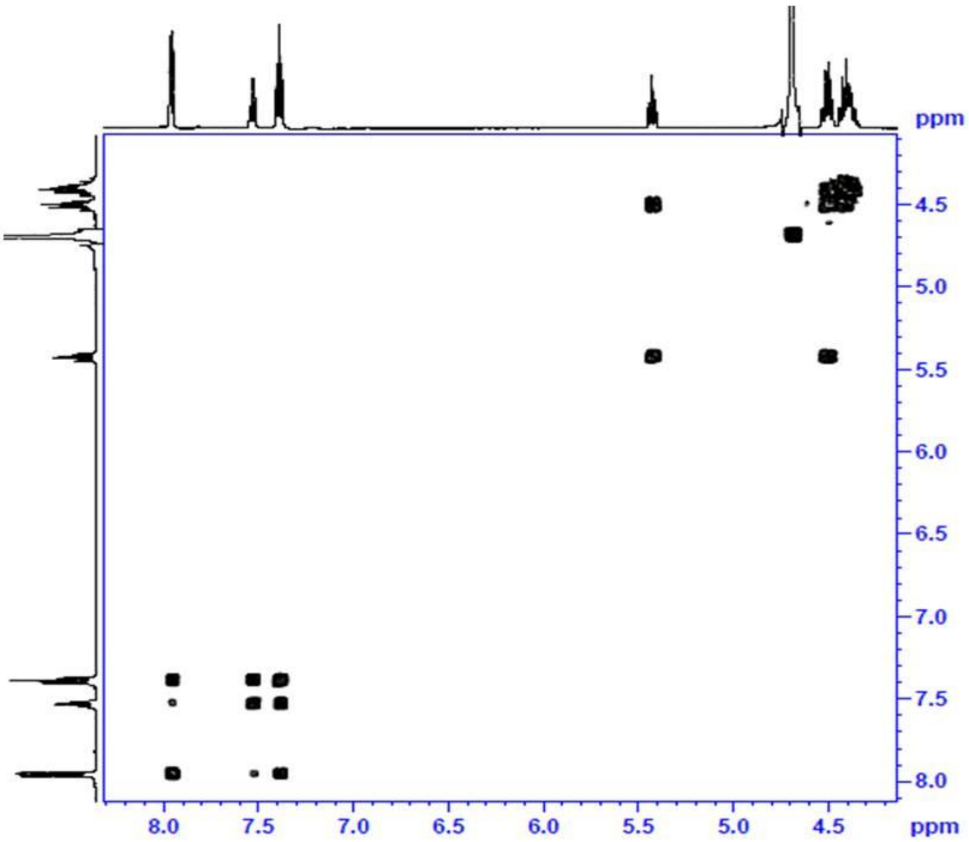

DEPT of compound 8

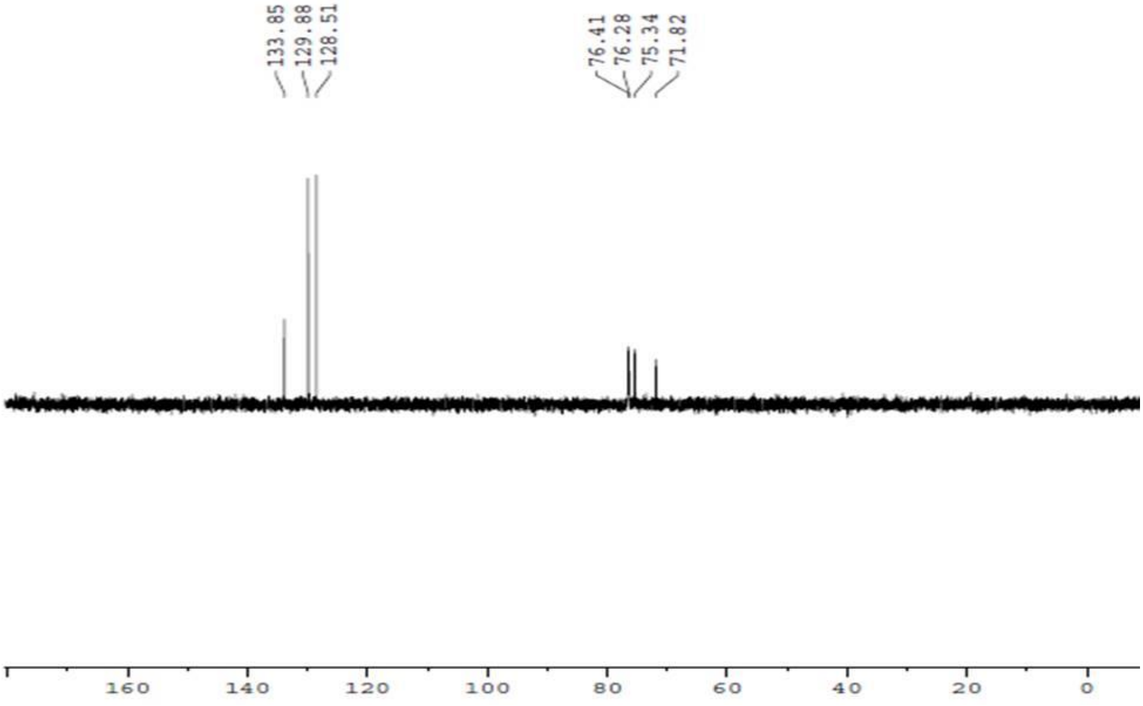

^{13}C of compound 8

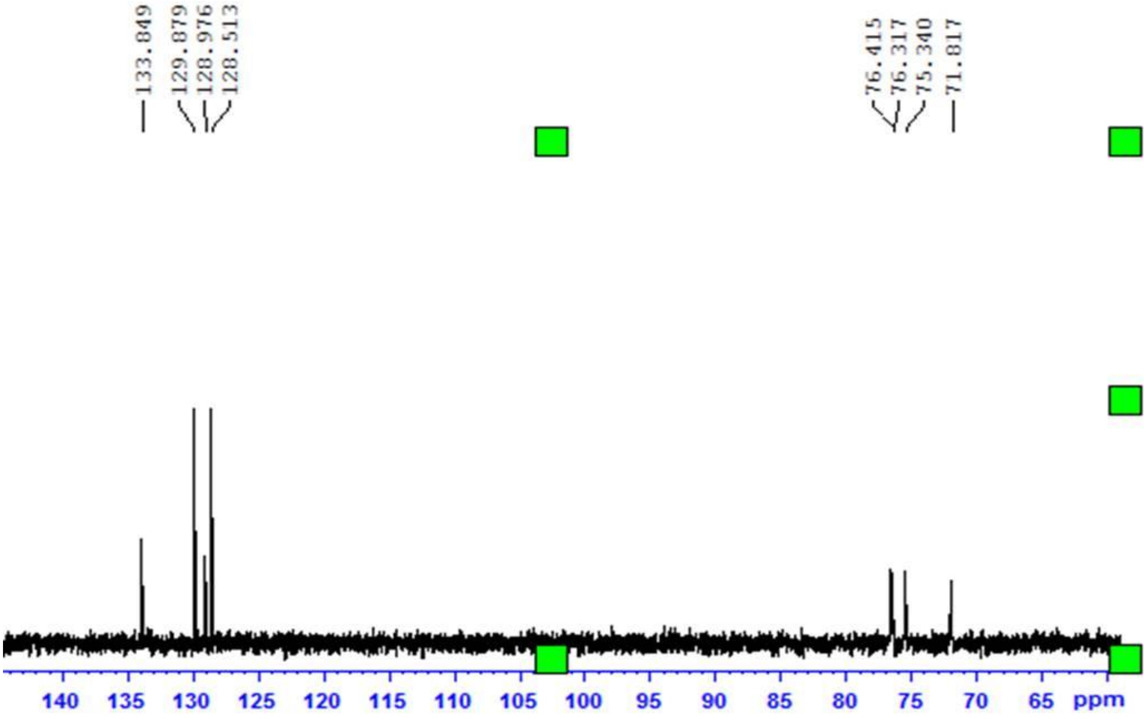

HMQC of compound 8

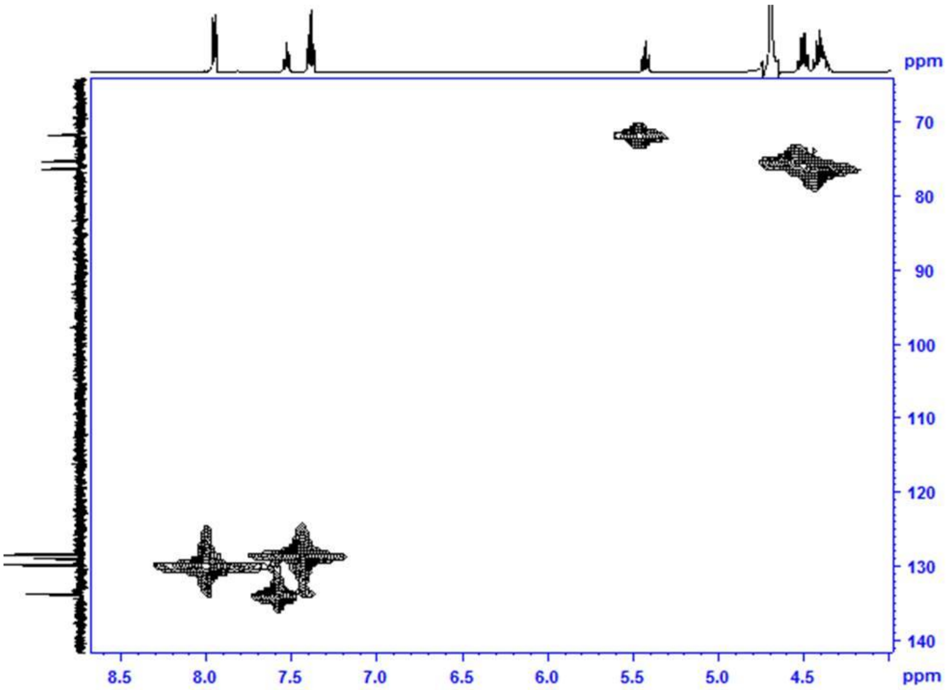

^{1}H NMR of compound 9

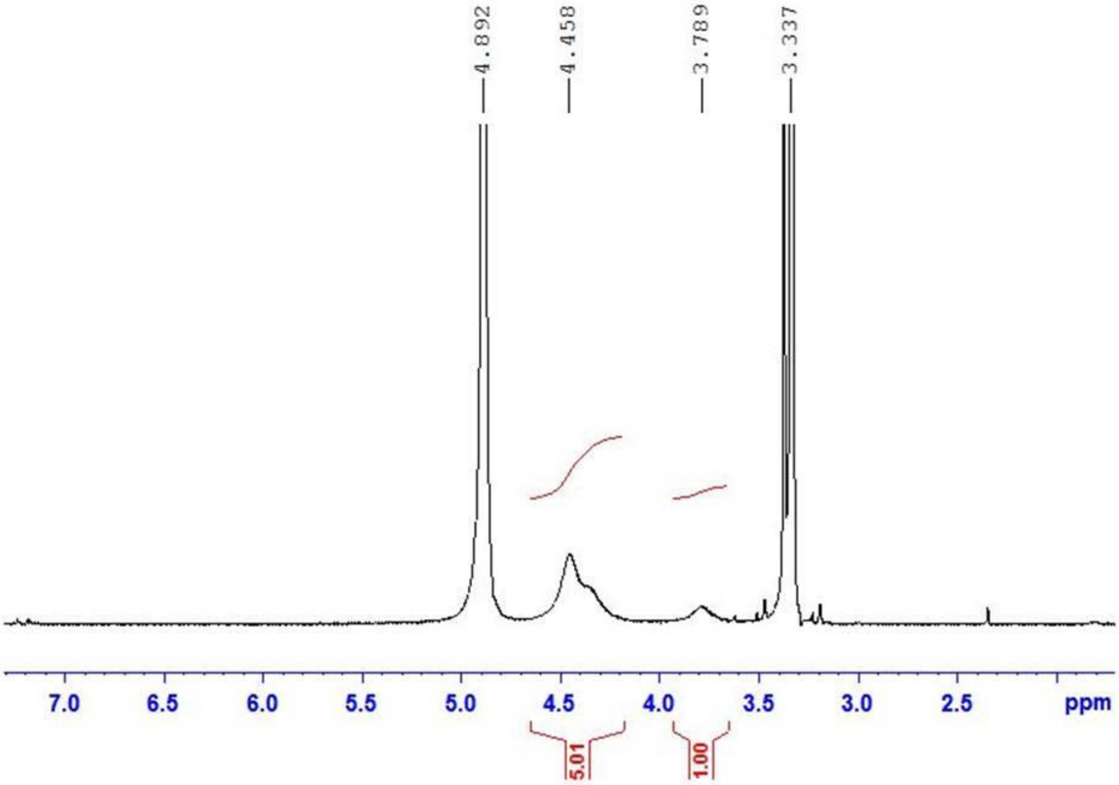

^{31}P of compound 9

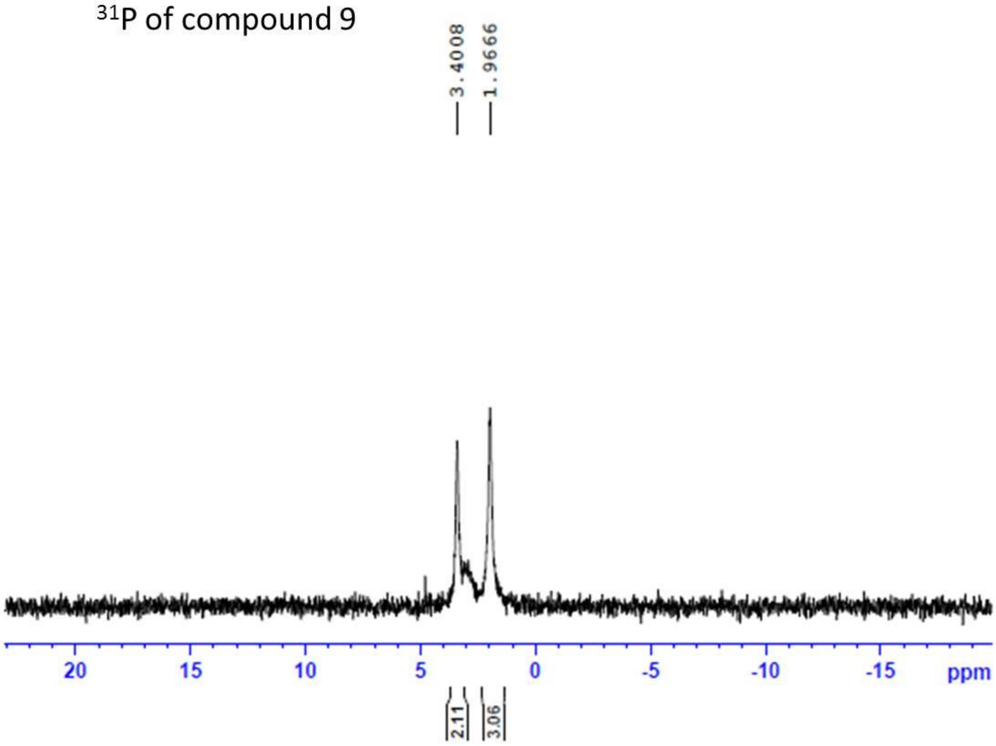

^{1}H NMR of compound 11

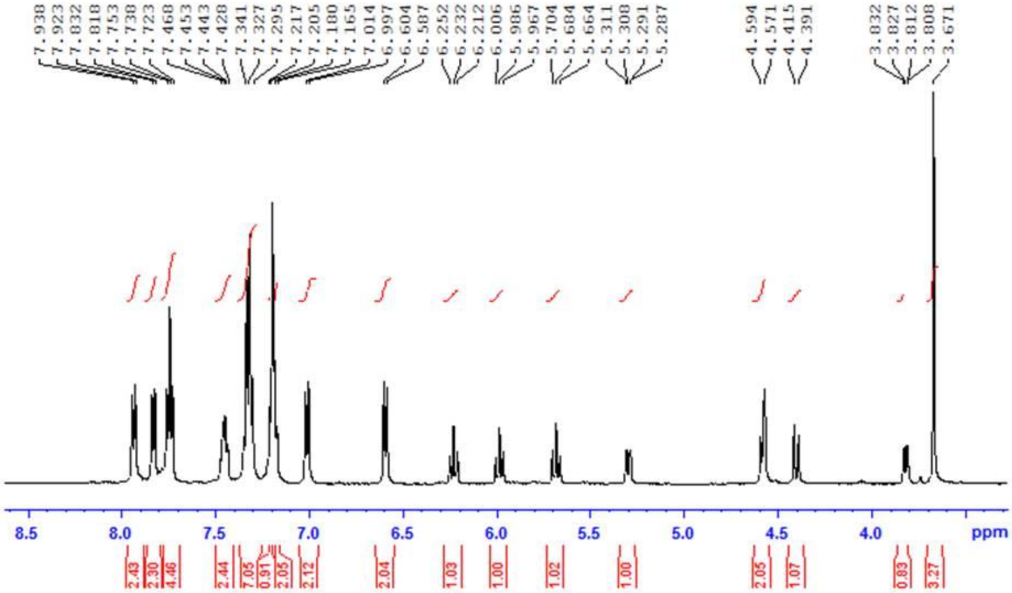

COSY of compound 11

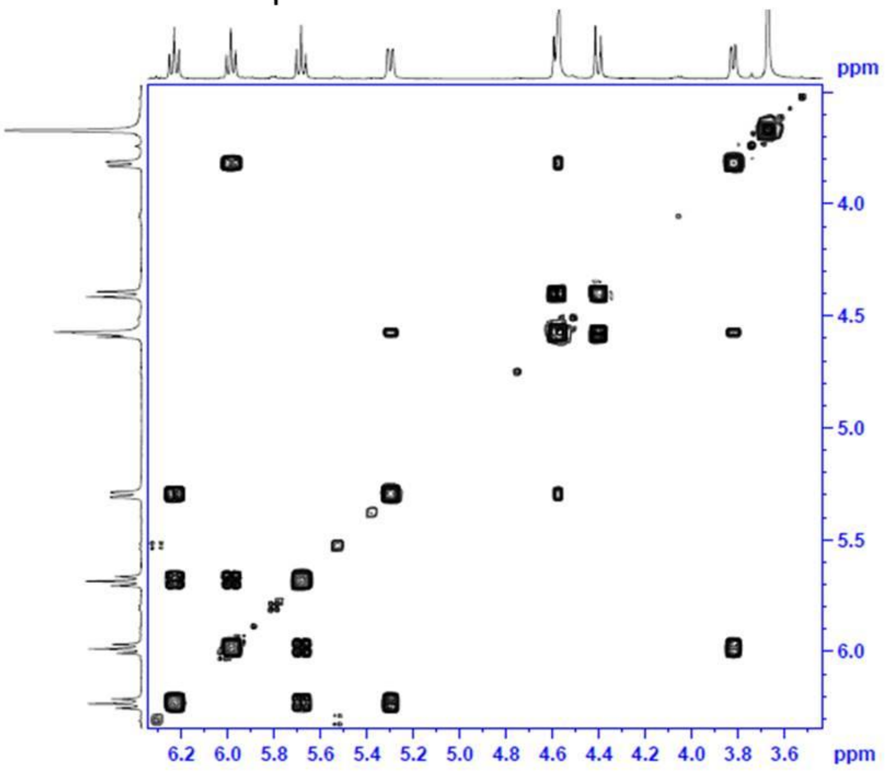

DEPT of compound 11

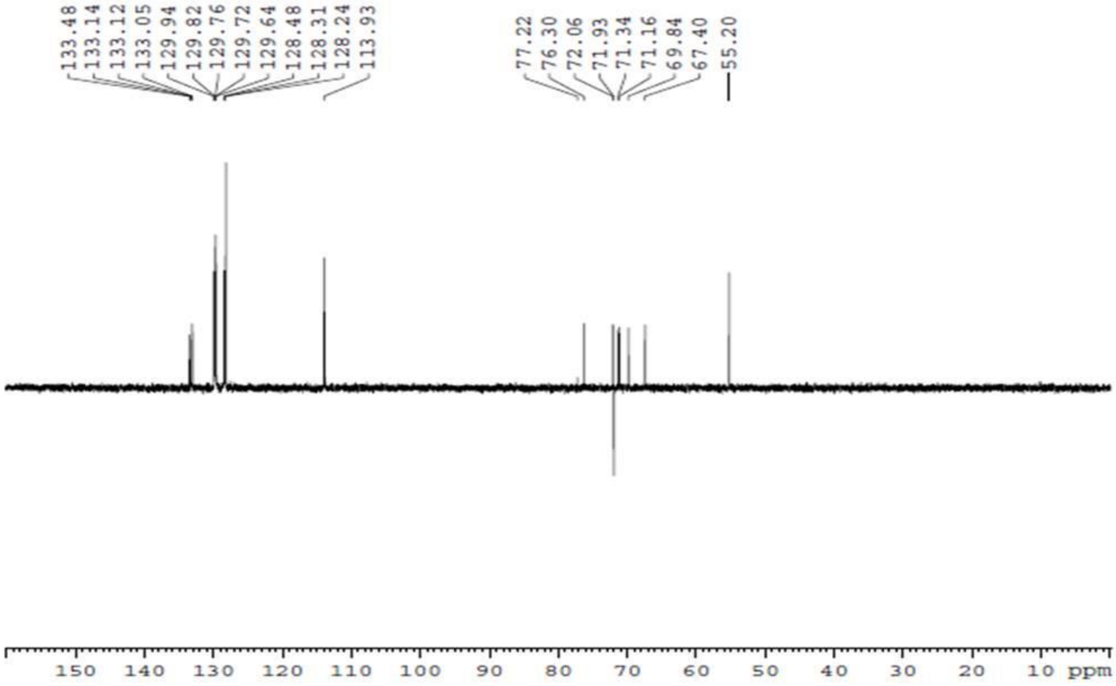

^{13}C of Compound 11

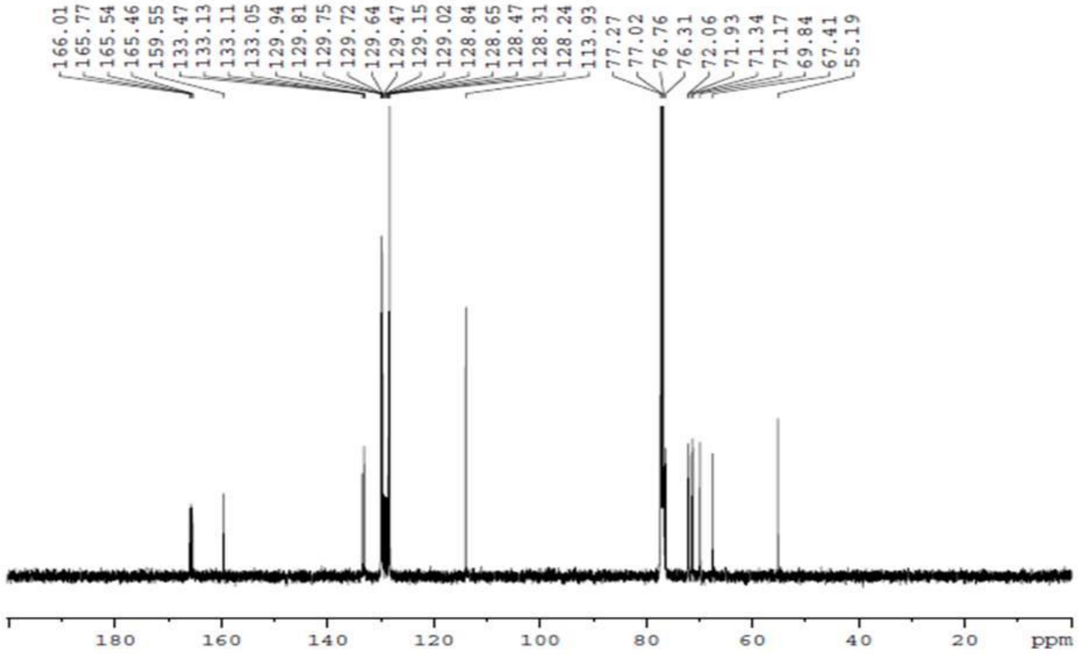

HMQC of compound 11

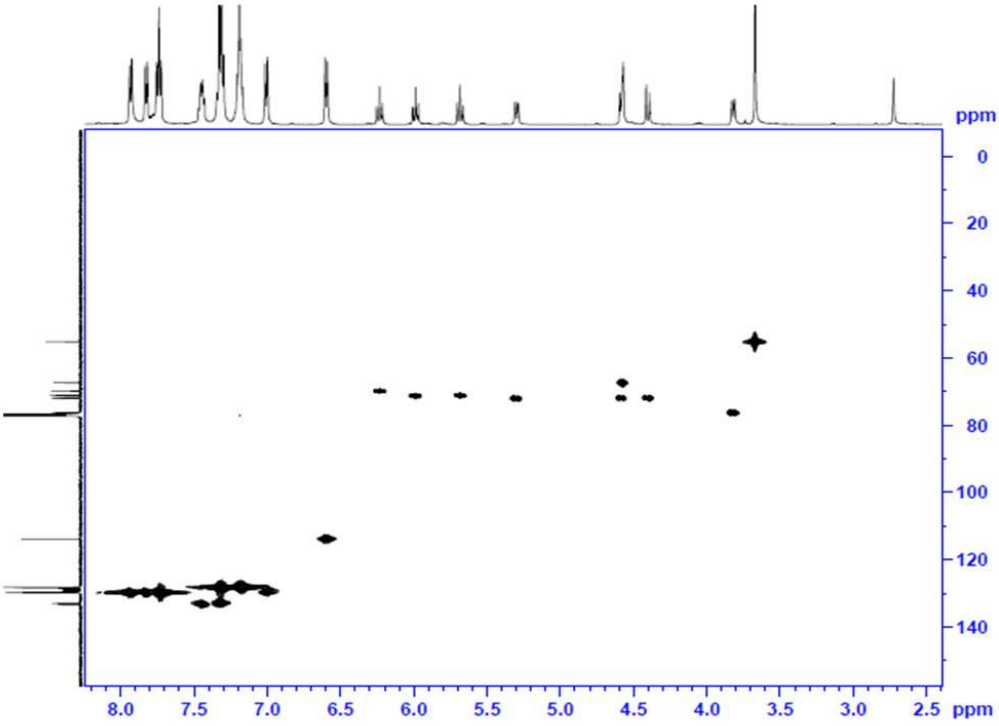

^{1}H NMR of compound 12

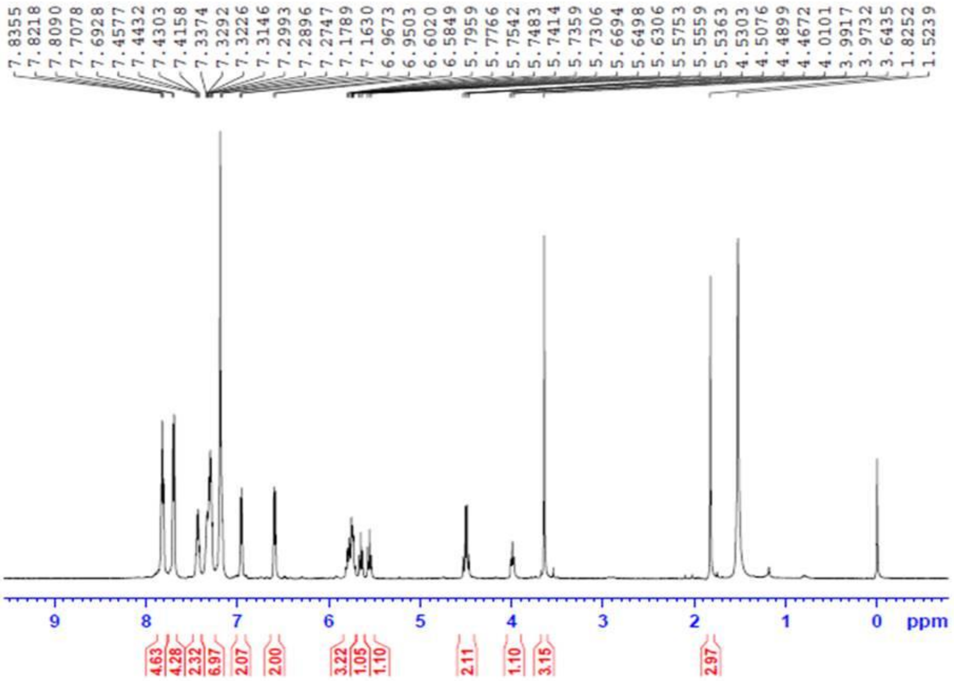

COSY of compound of 12

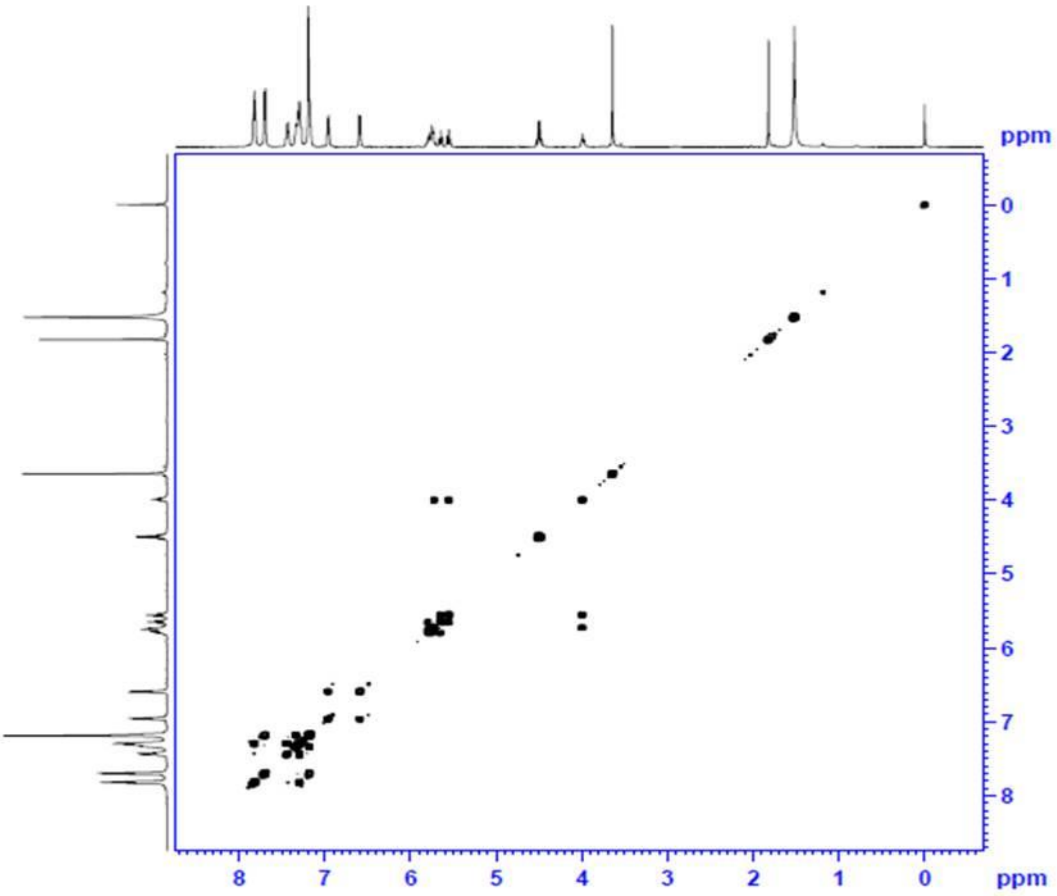

DEPT of compound 12

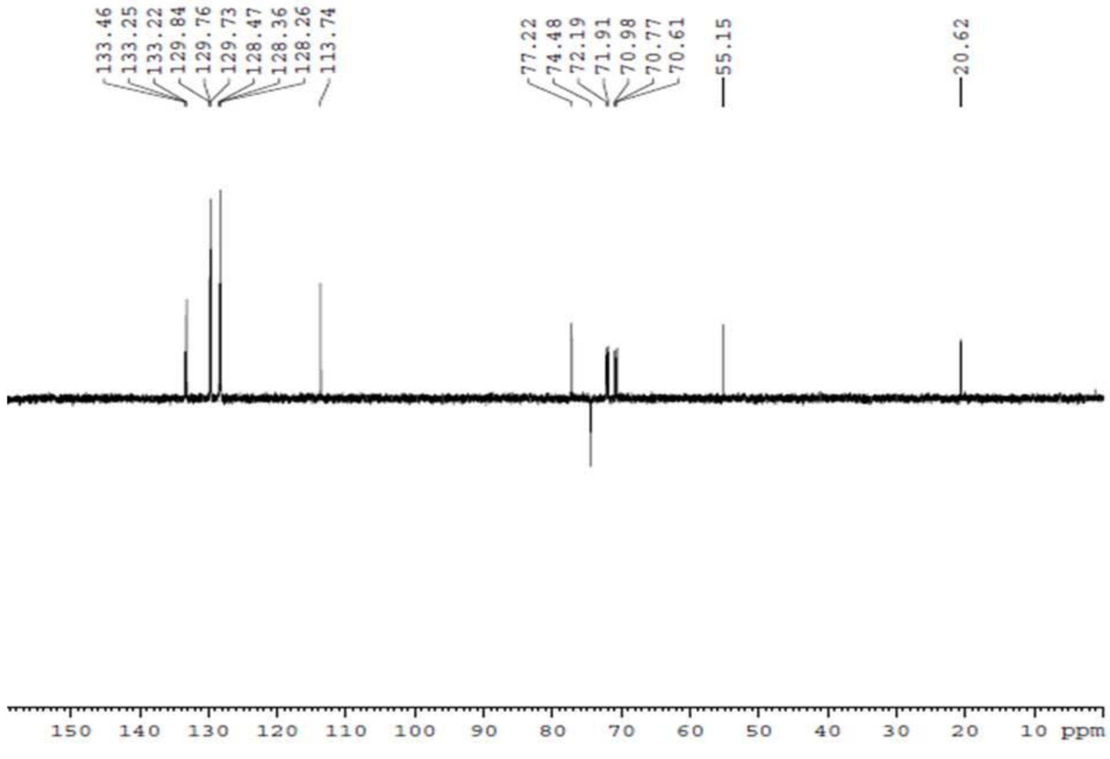

[13]C of compound 12

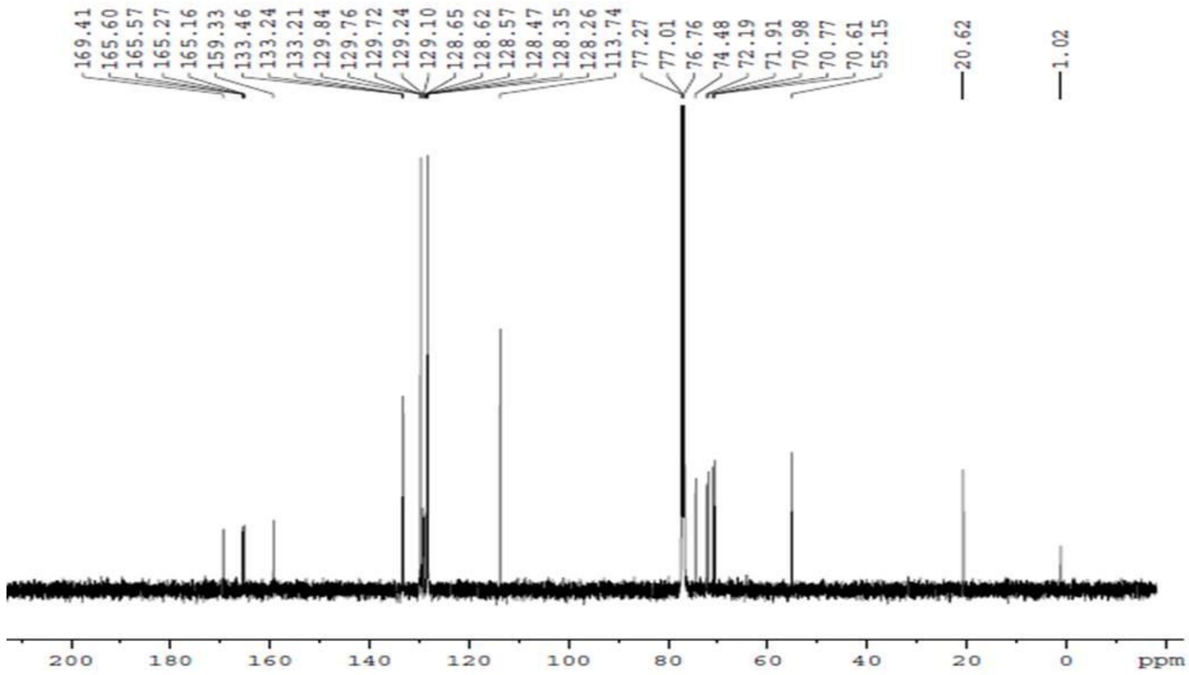

HMQC of compound 12

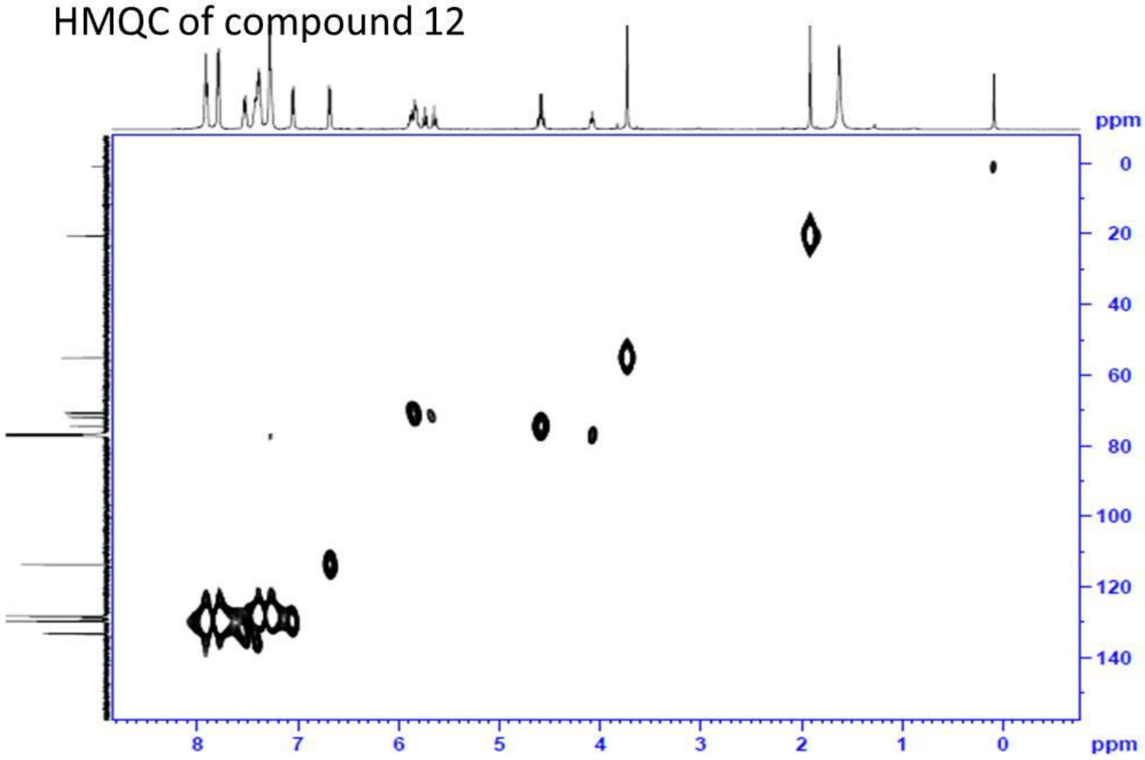

^{1}H NMR of compound 13

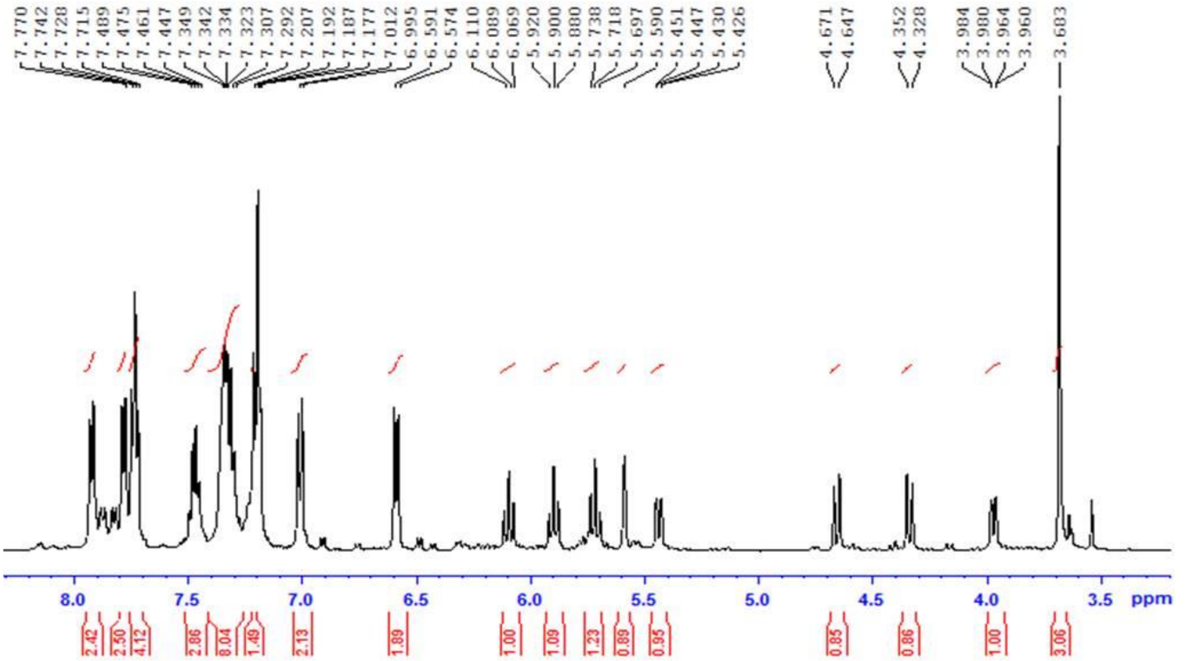

COSY of compound 13

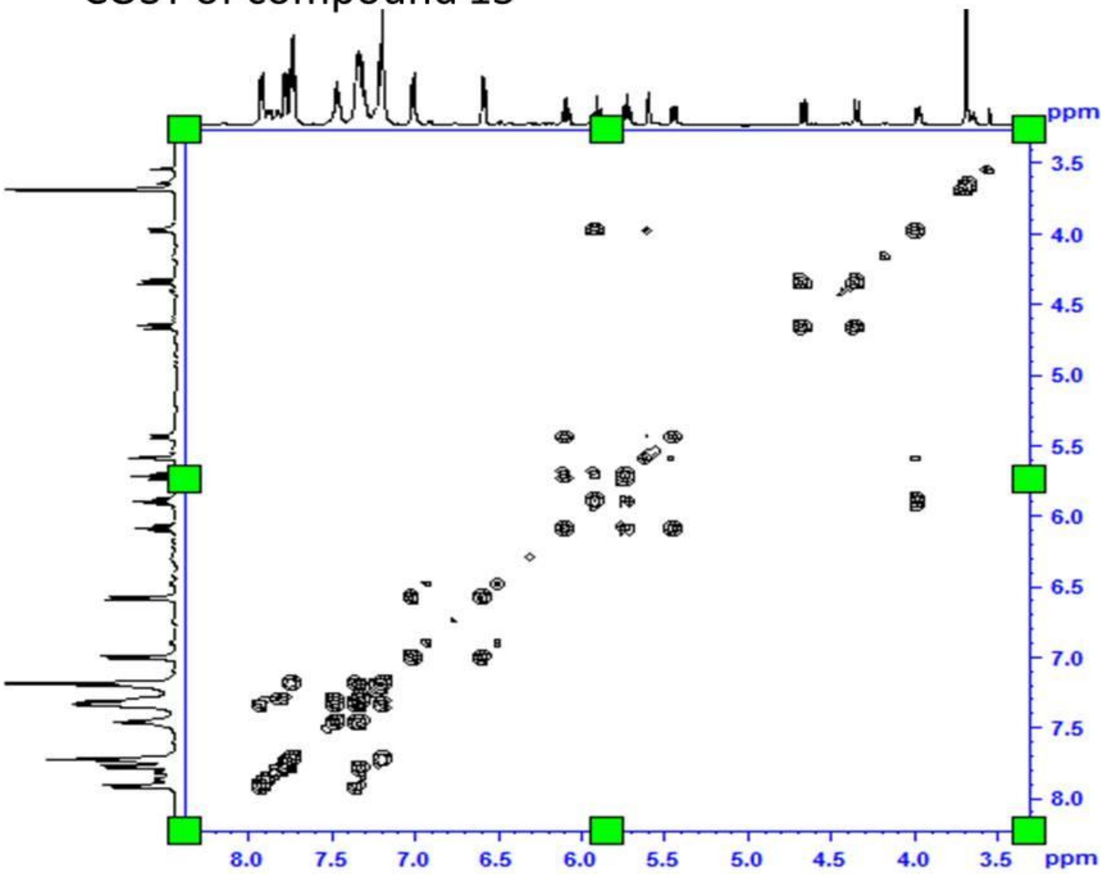

DEPT of compound 13

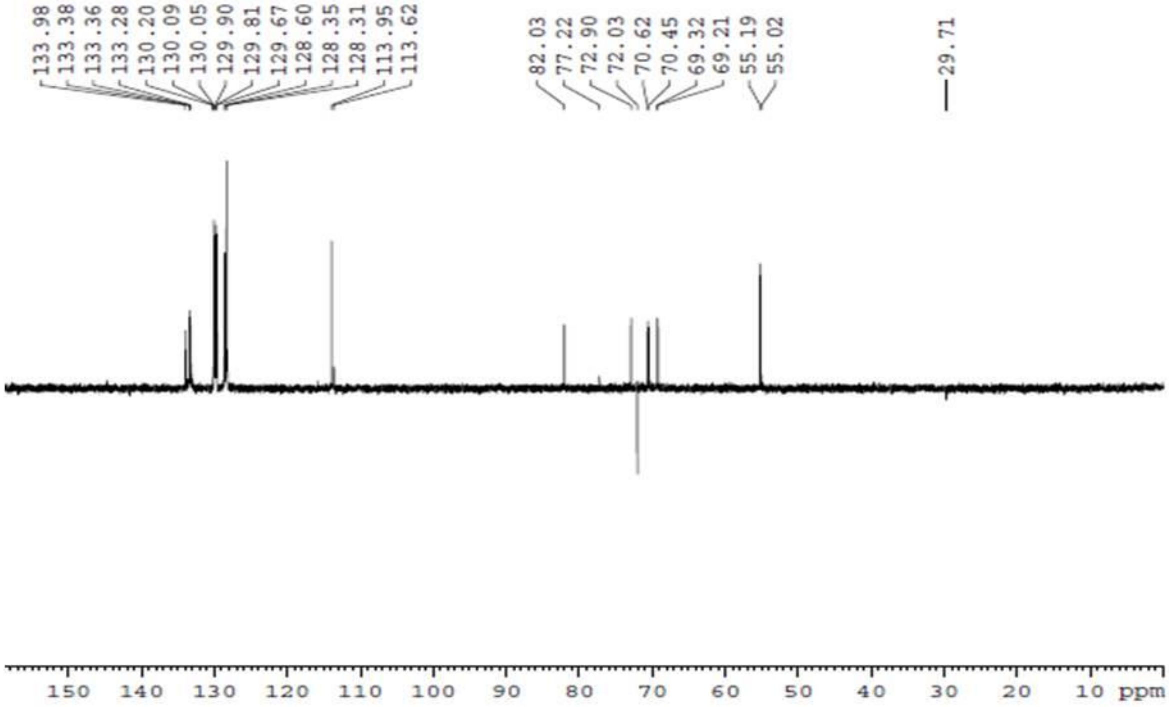

^{13}C of compound 13

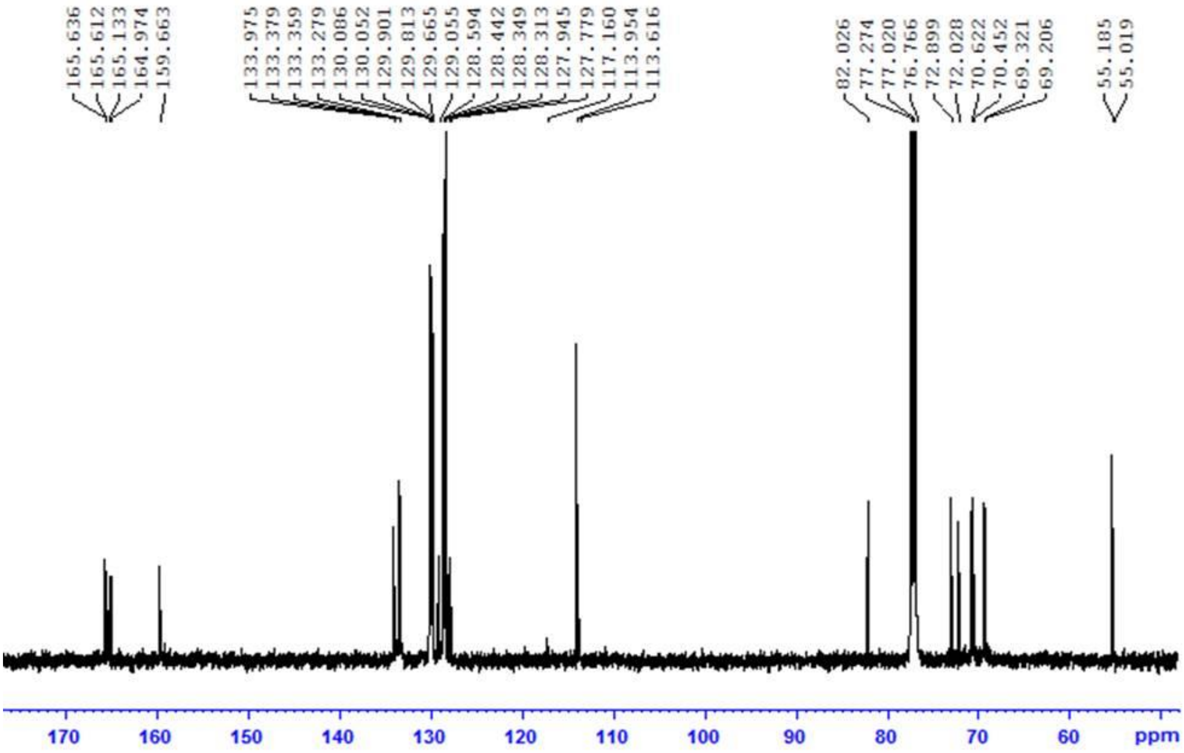

HMQC of compound 13

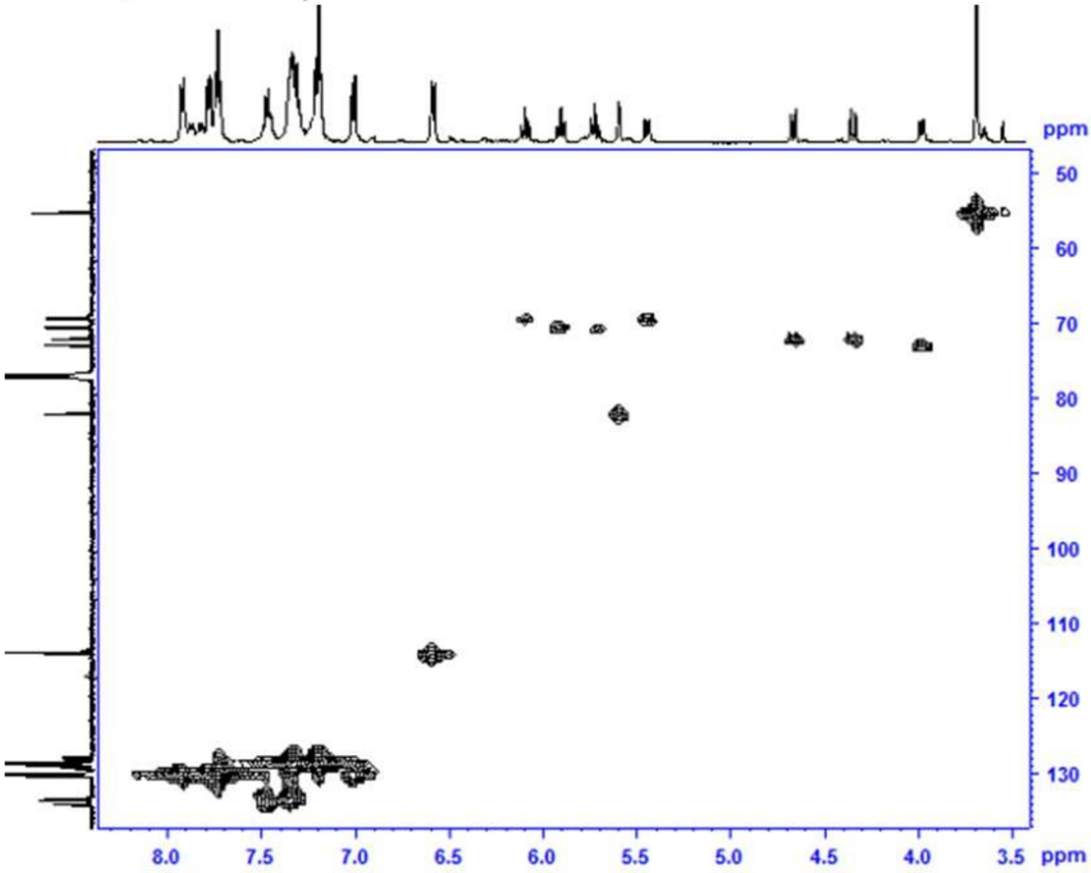

^{1}H NMR of compound 14

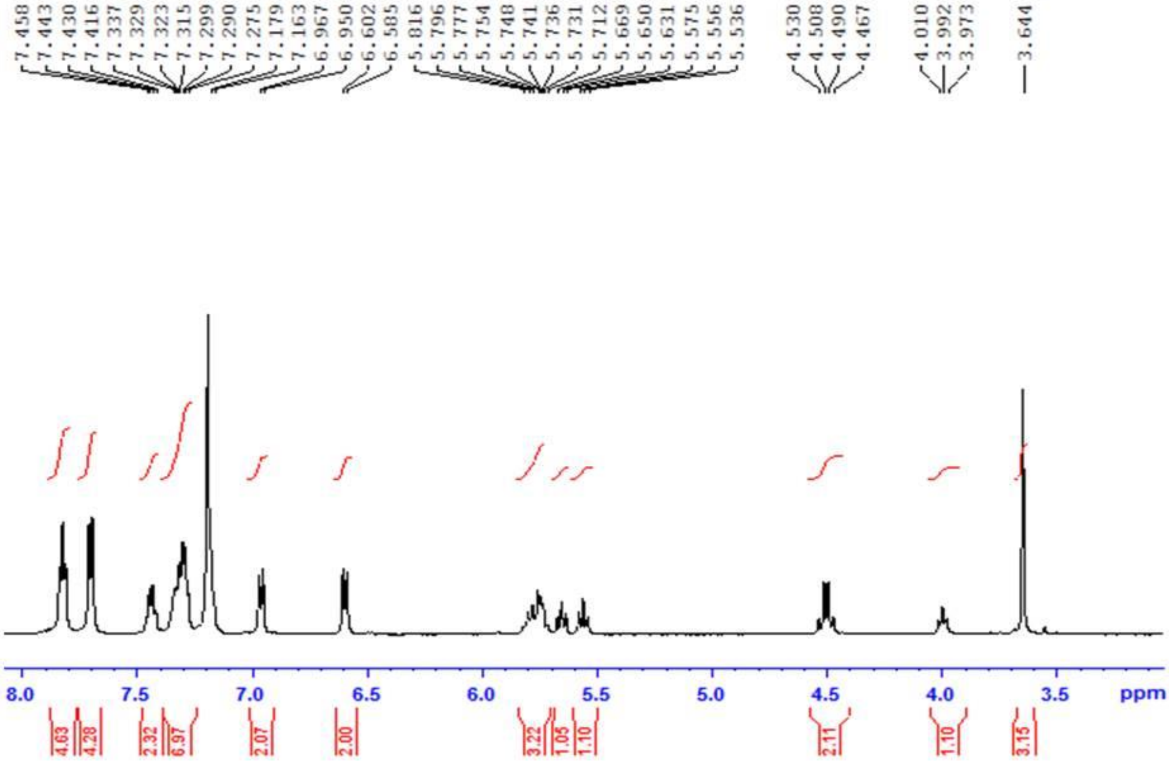

COSY of compound 14

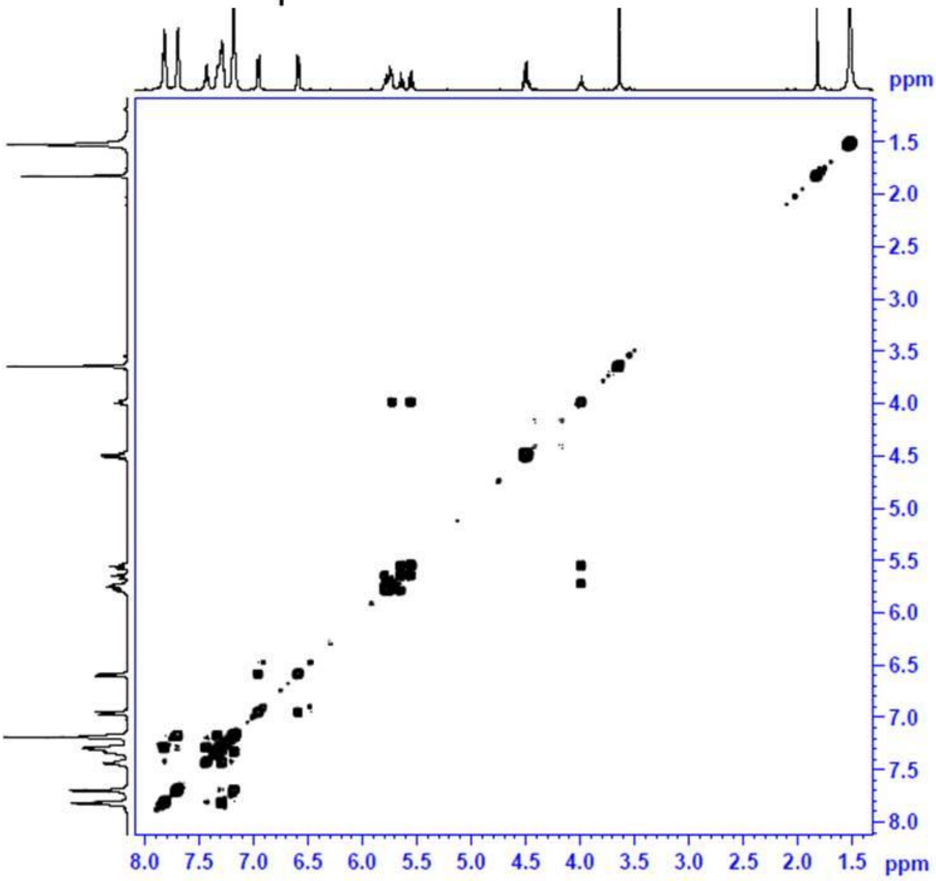

DEPT of compound 13

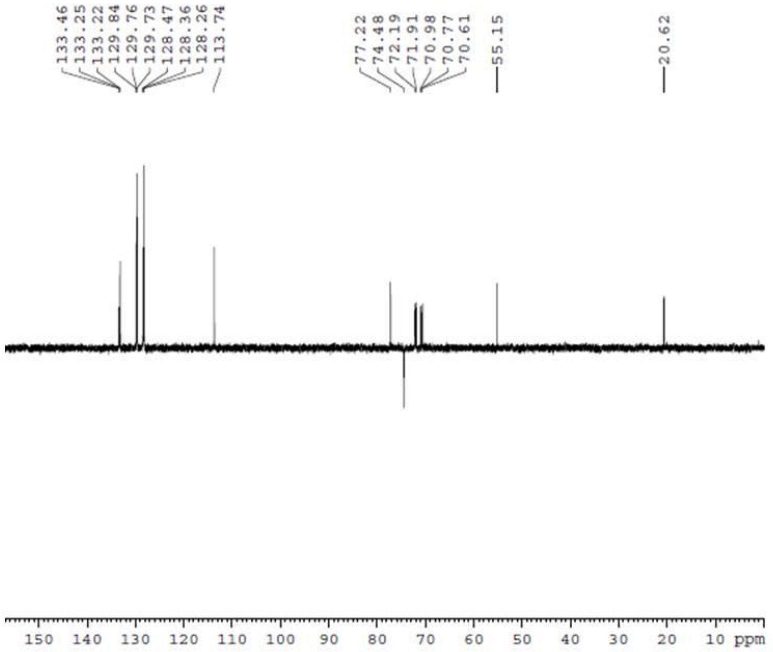

^{13}C of compound 13

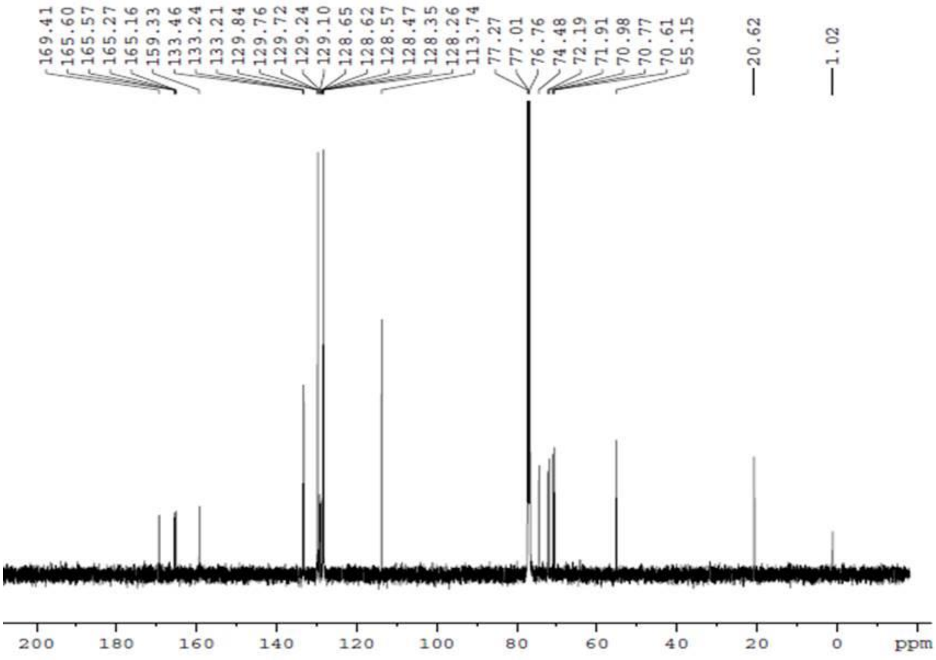

HMQC of compound 13

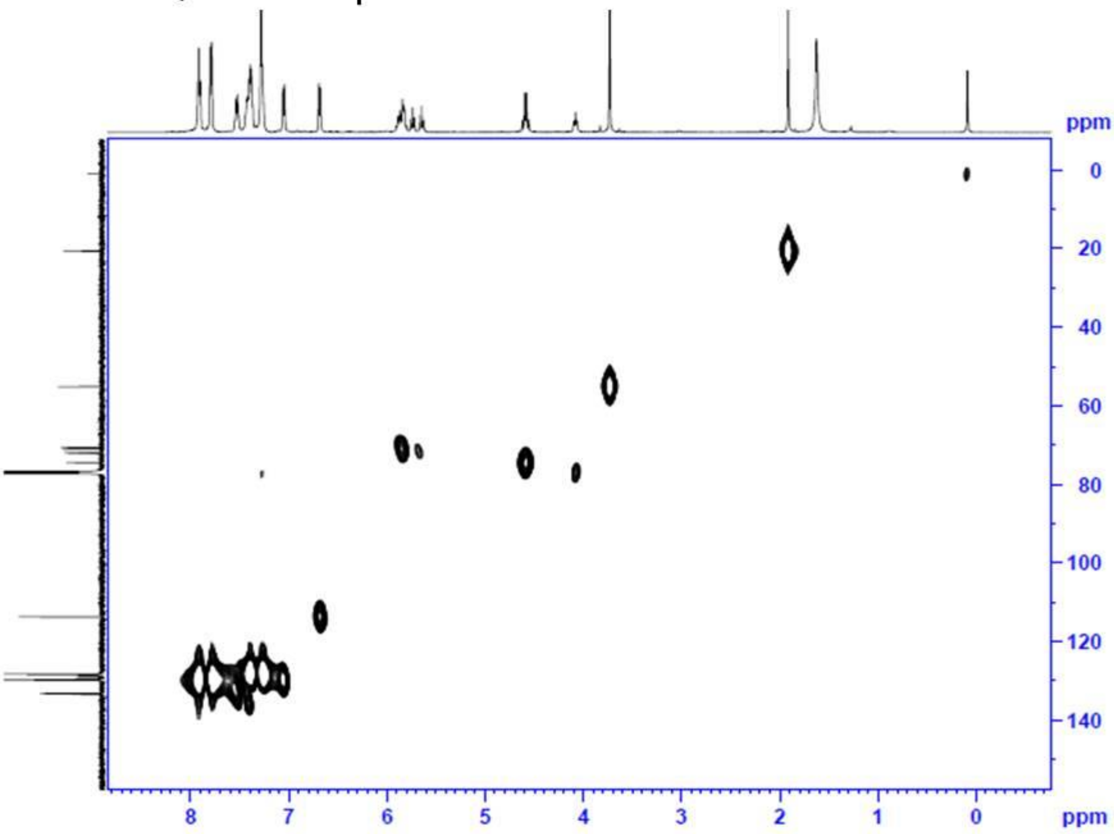

^{1}H NMR of compound 14

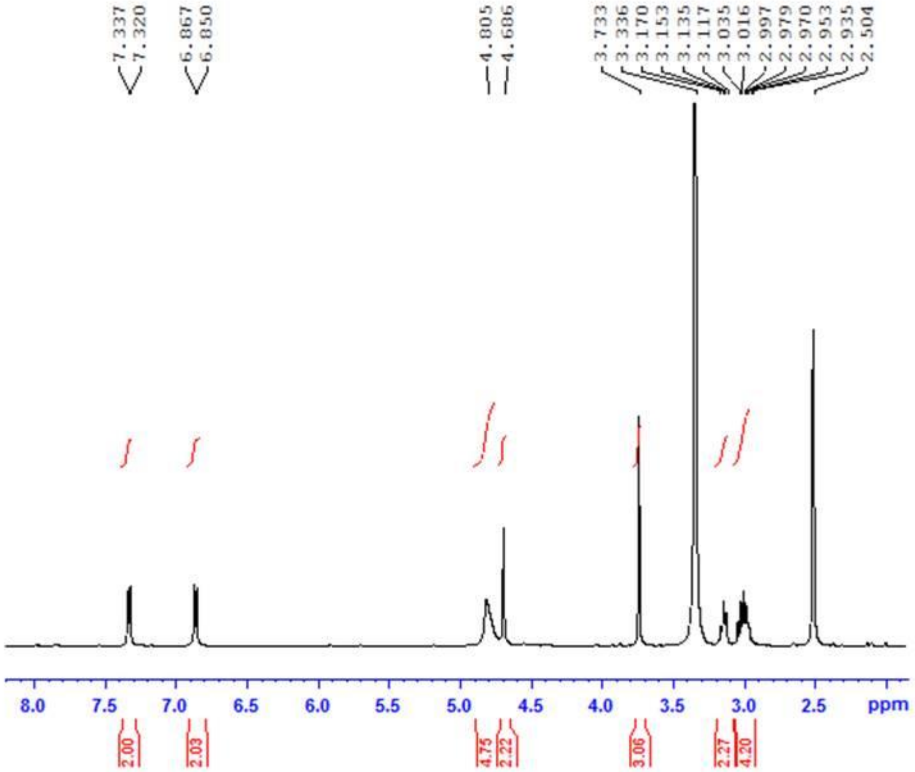

COSY of compound 14

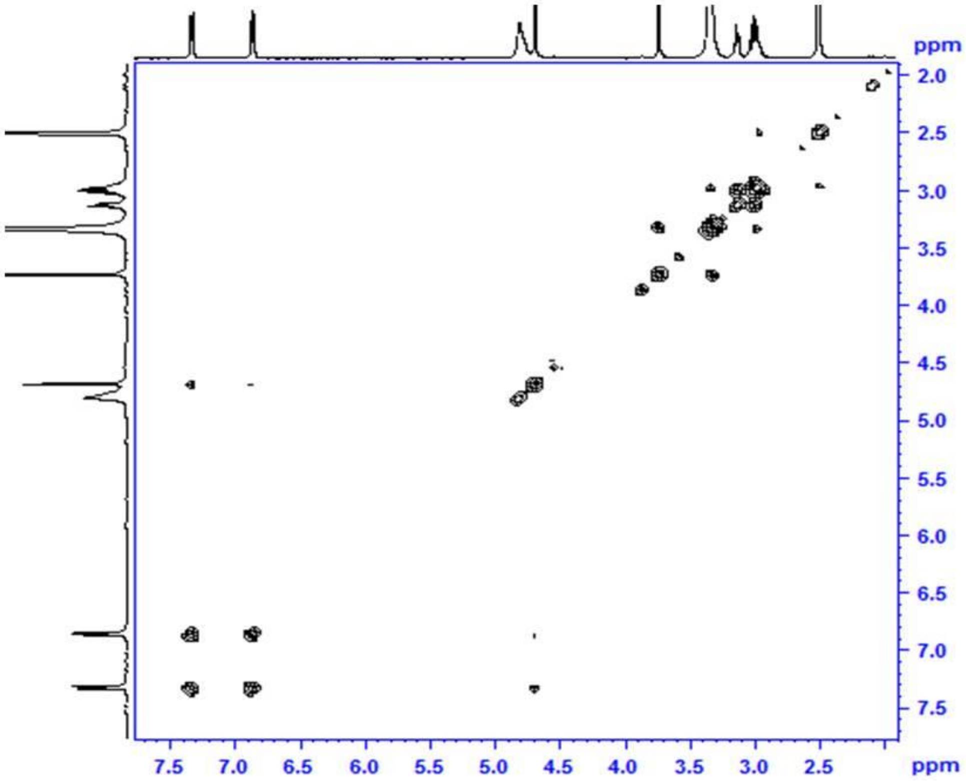

DEPT of compound 14

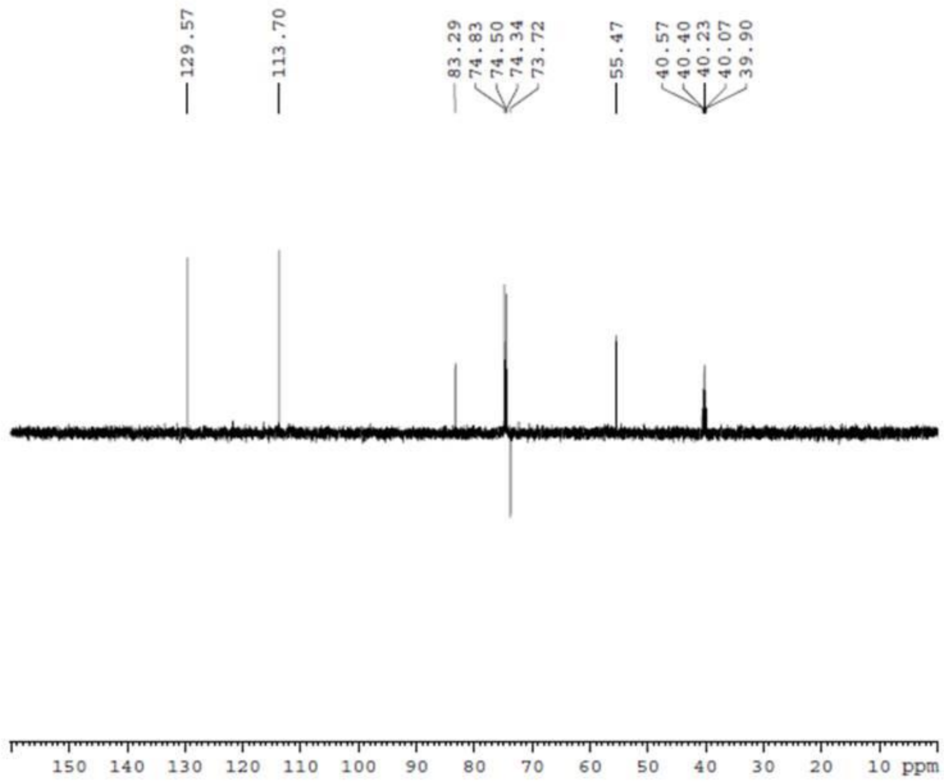

^{13}C of compound 14

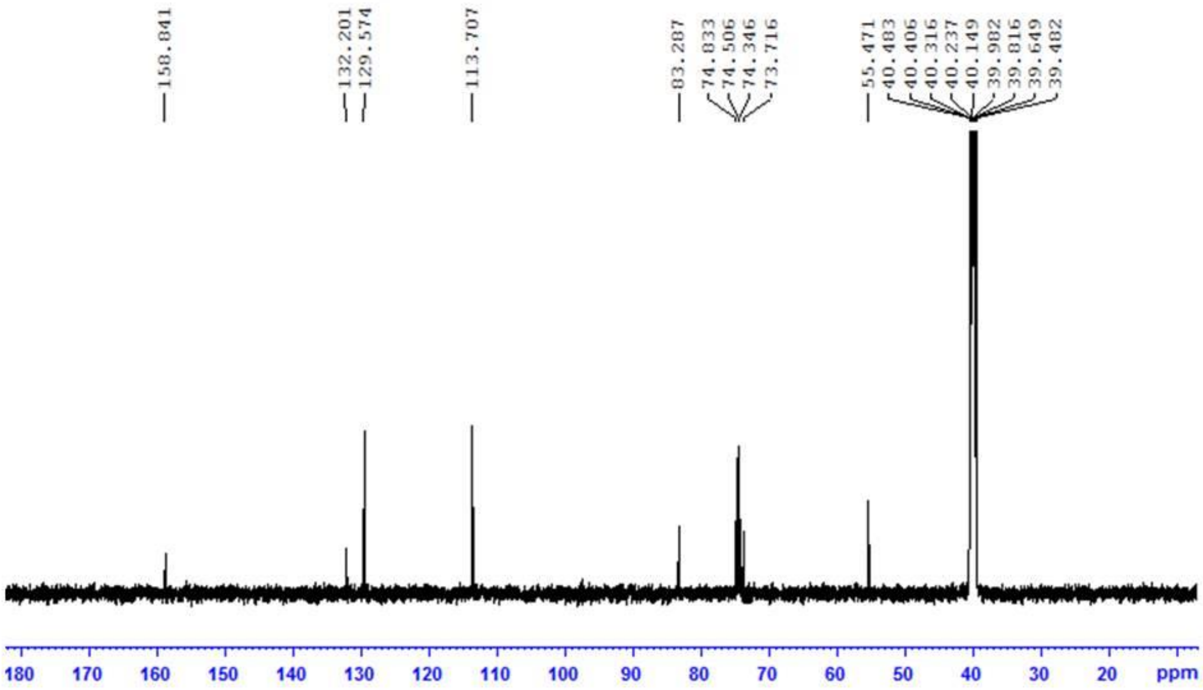

HMQC of compound 14

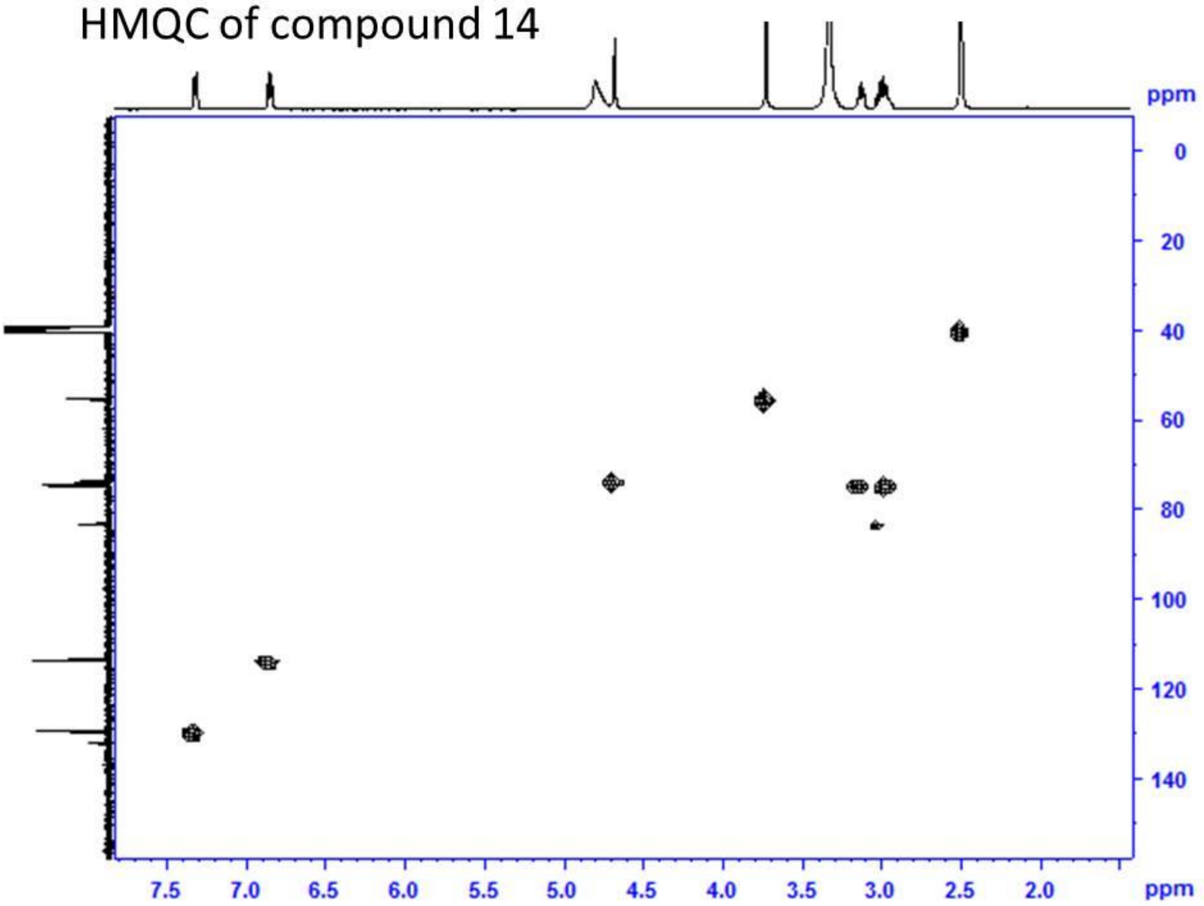

^{1}H NMR of compound 15

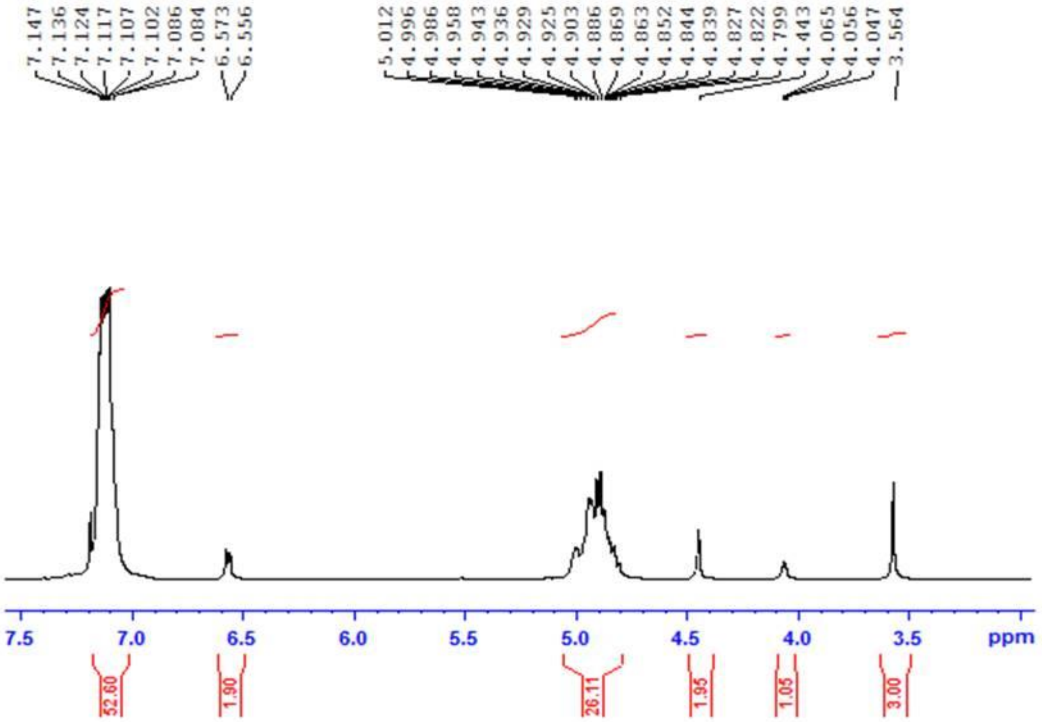

COSY of compound 15

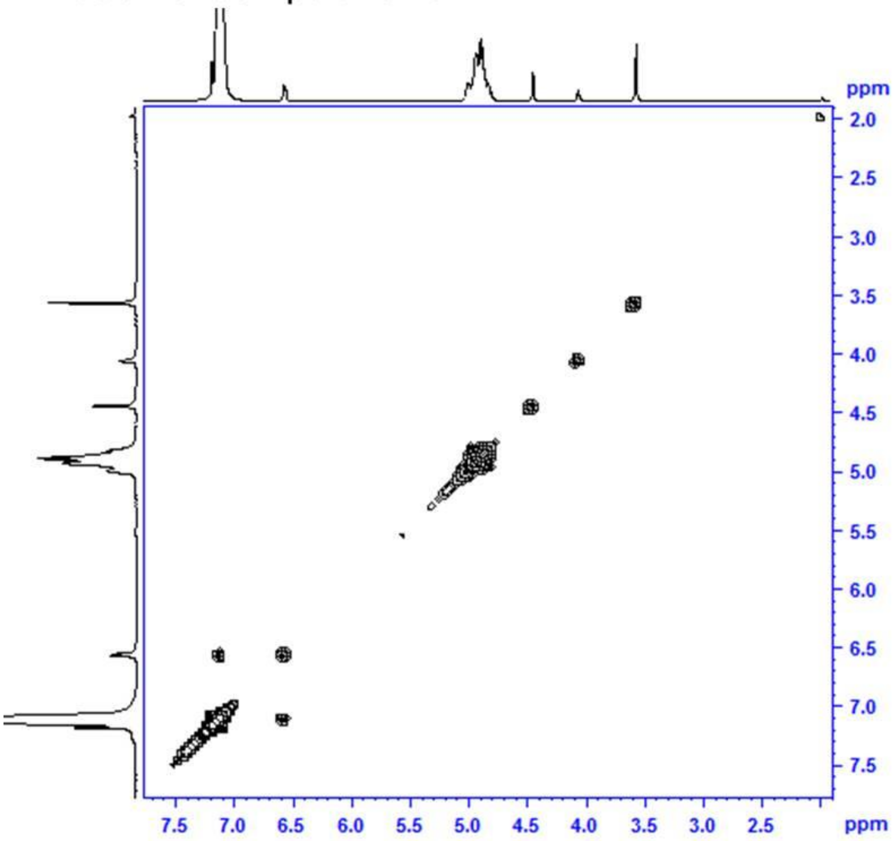

DEPT of compound 15

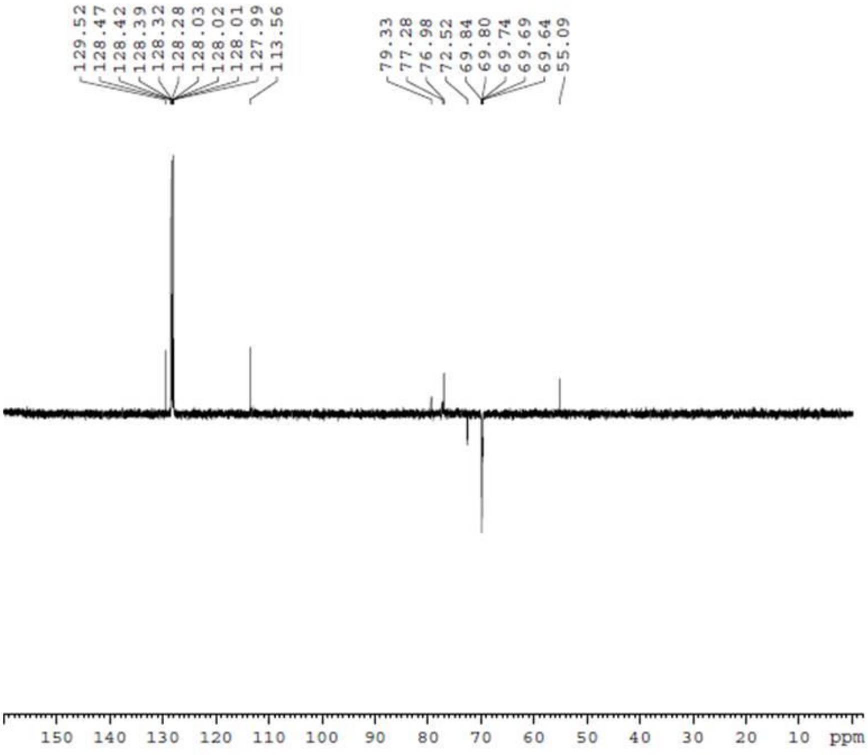

^{13}C of compound 15

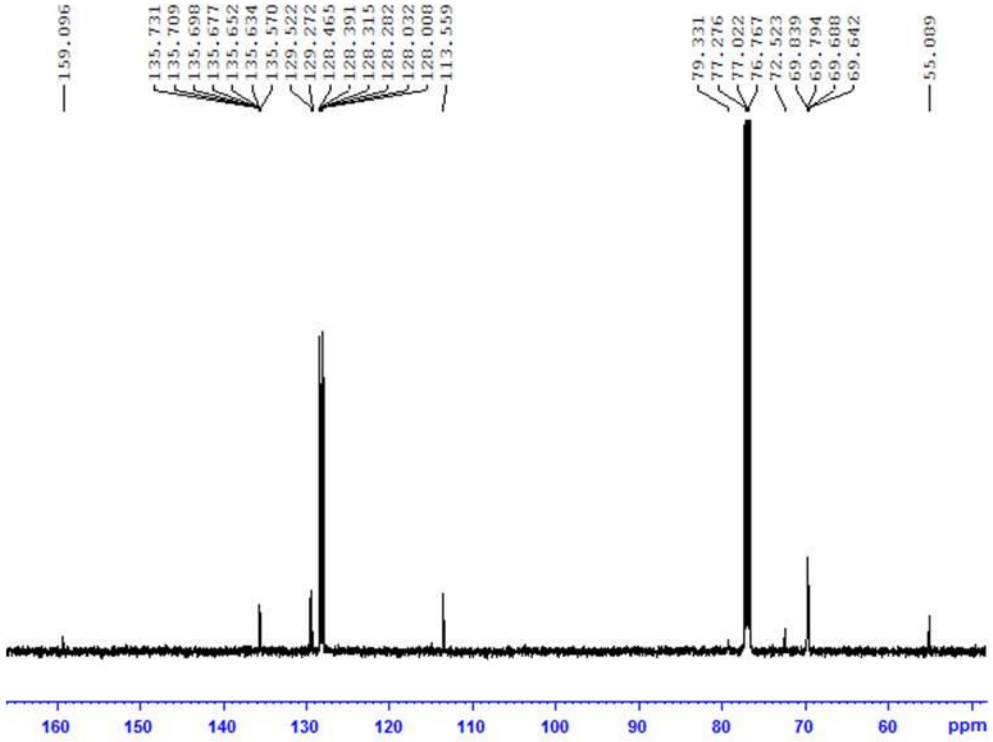

HMQC of compound 15

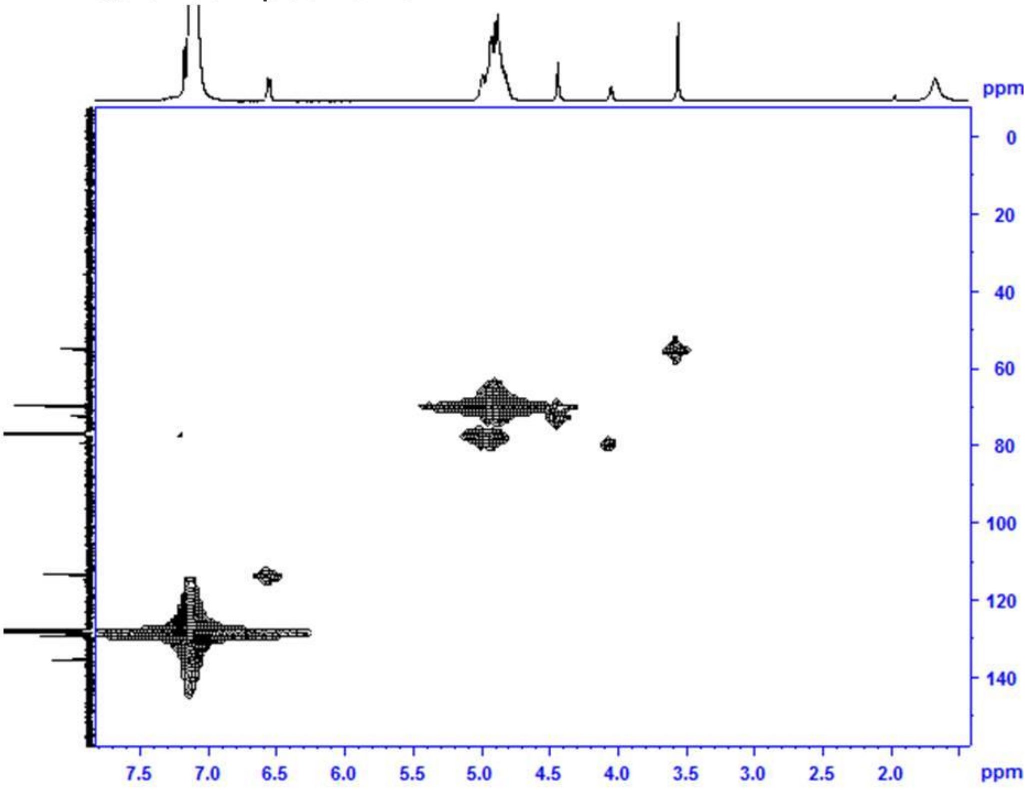

^{31}P of compound 15

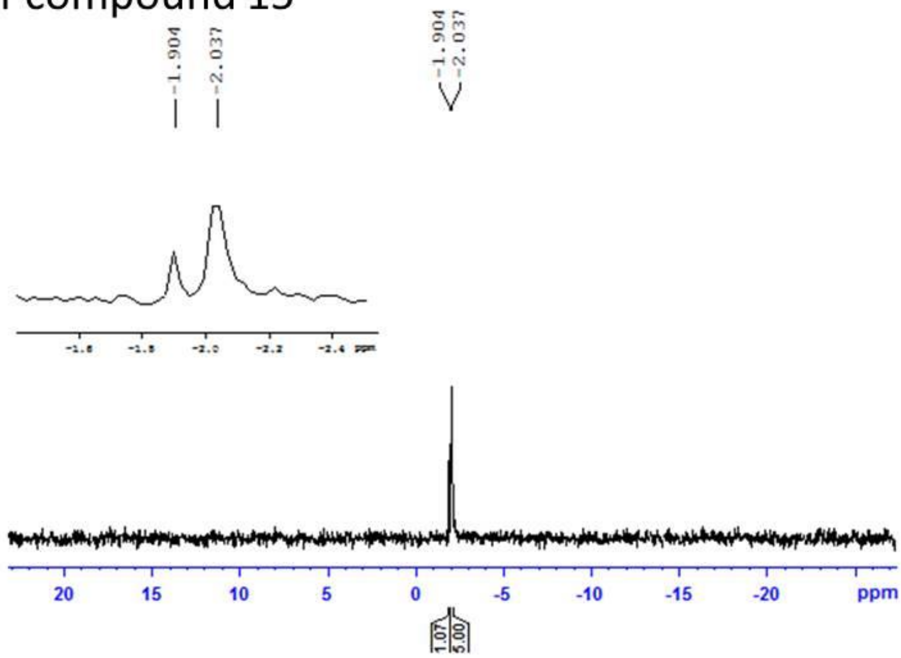

^{1}H NMR of compound 16

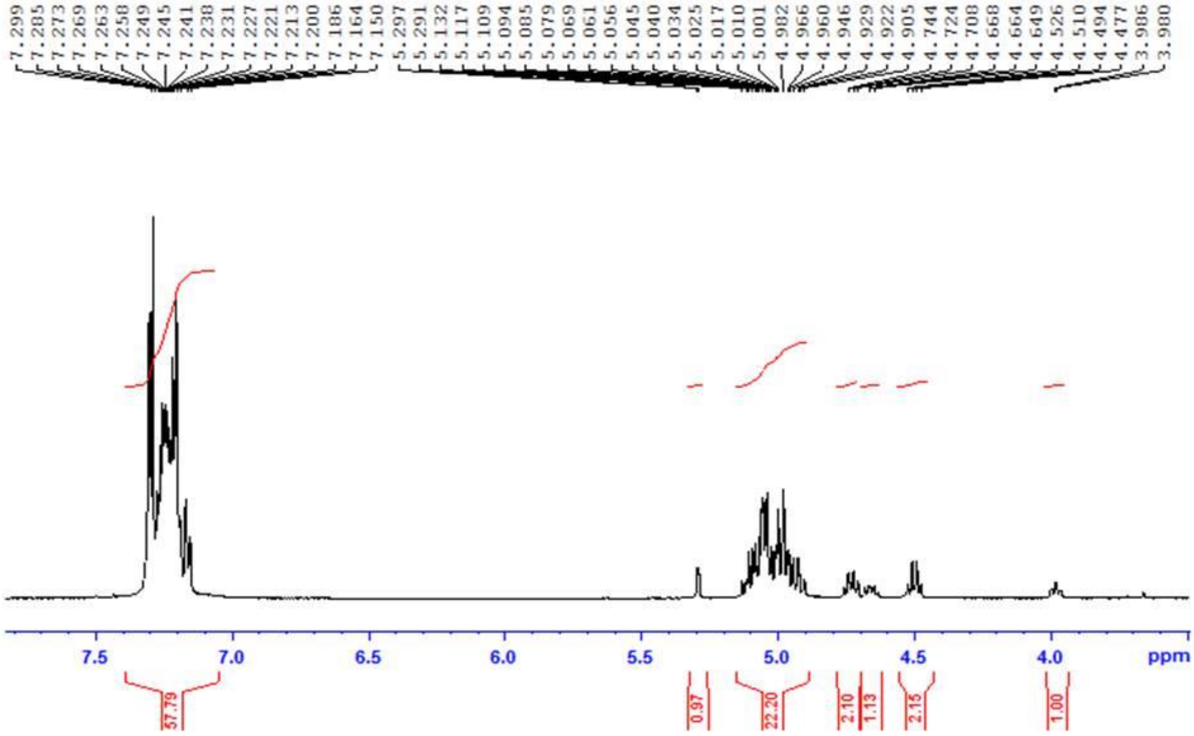

COSY of compound 16

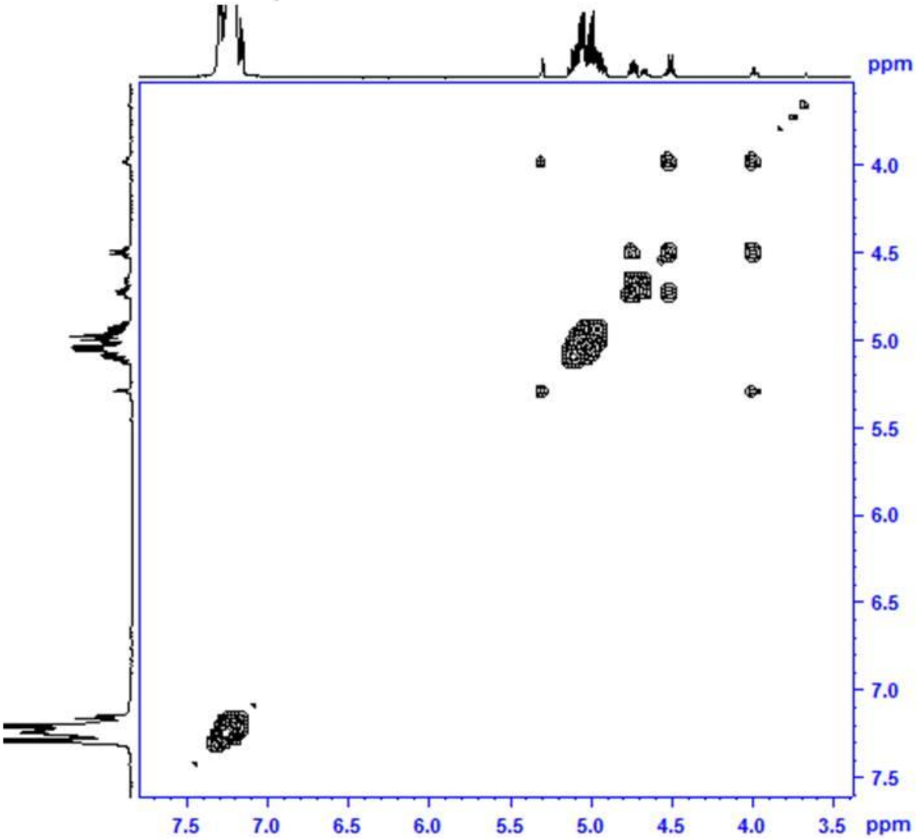

DEPT of compound 16

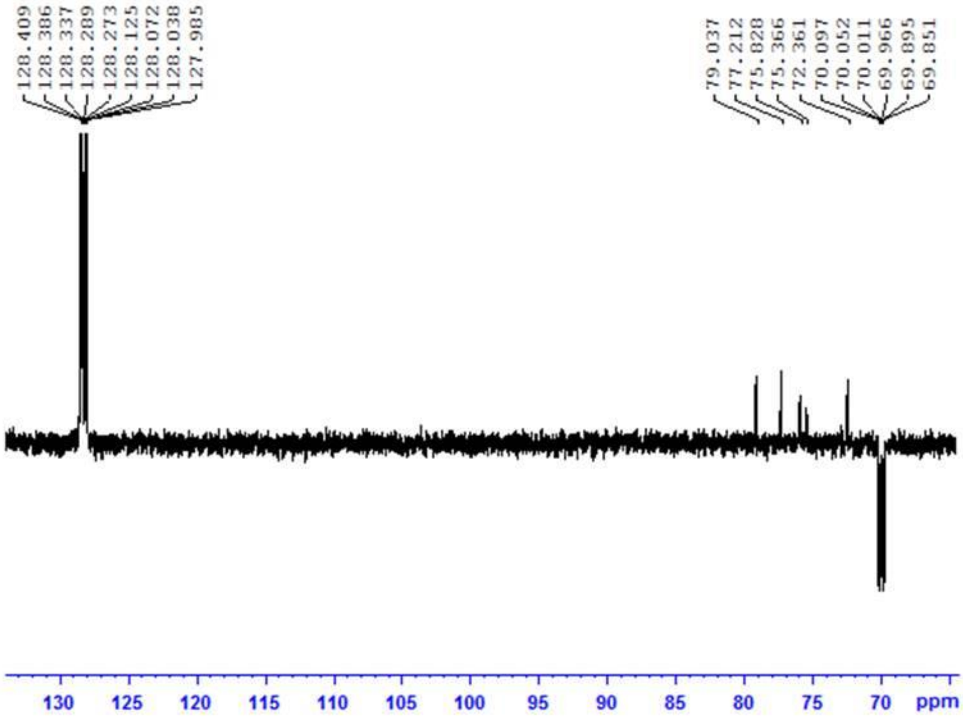

^{13}C of compound 16

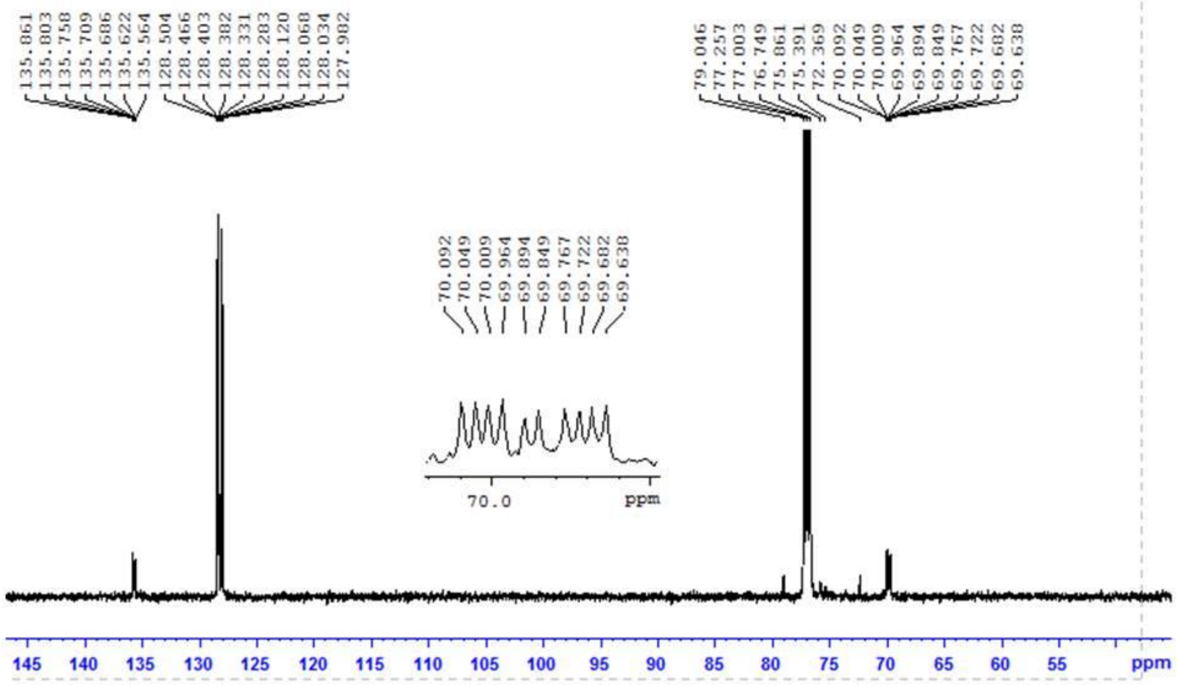

HMQC of compound 16

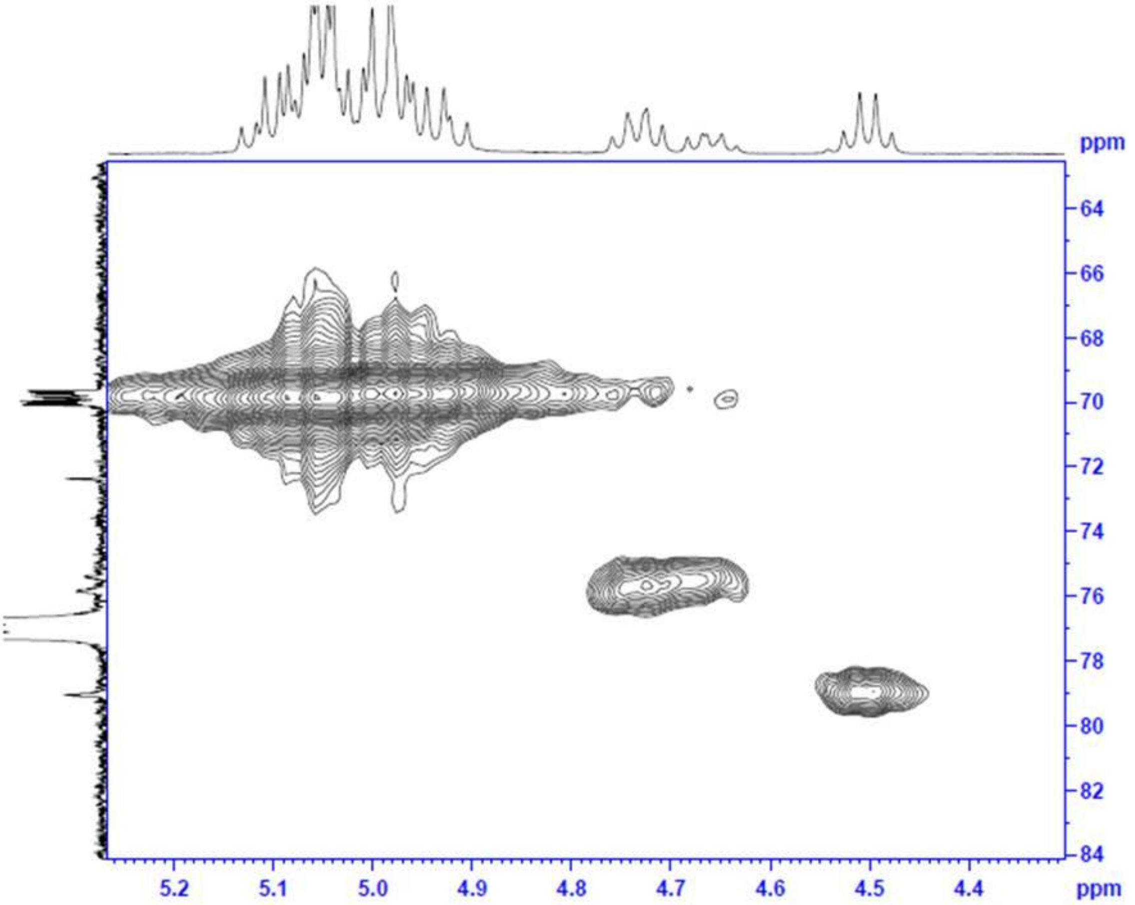

^{31}P of compound 16

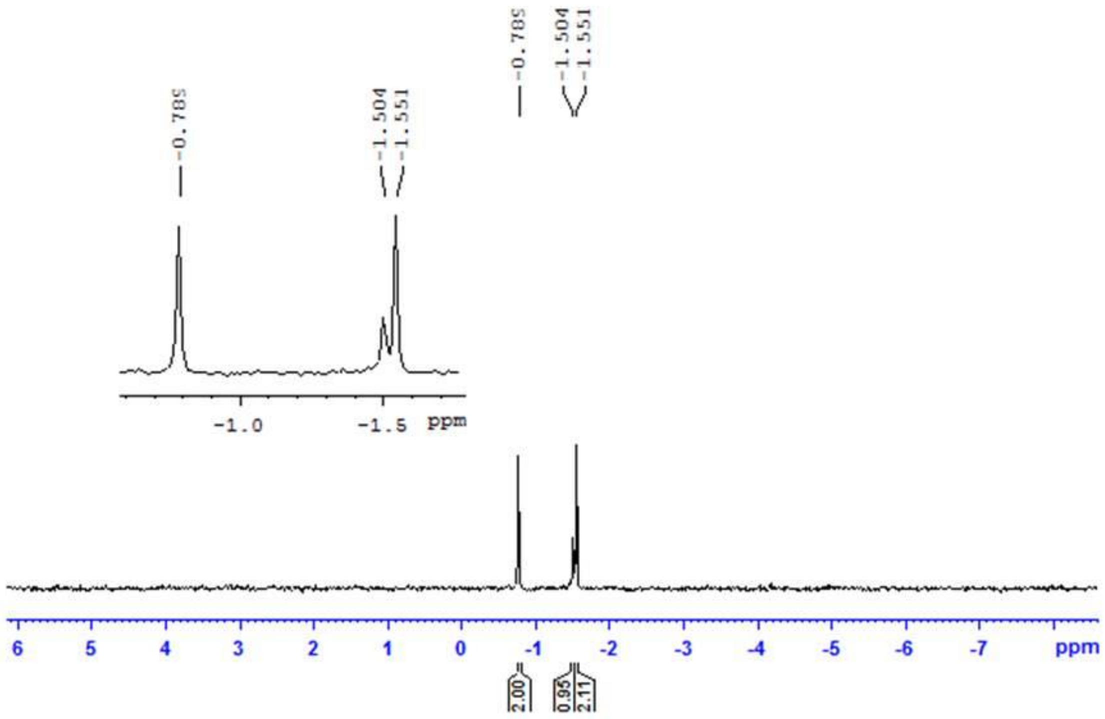

^{1}H NMR of compound 17

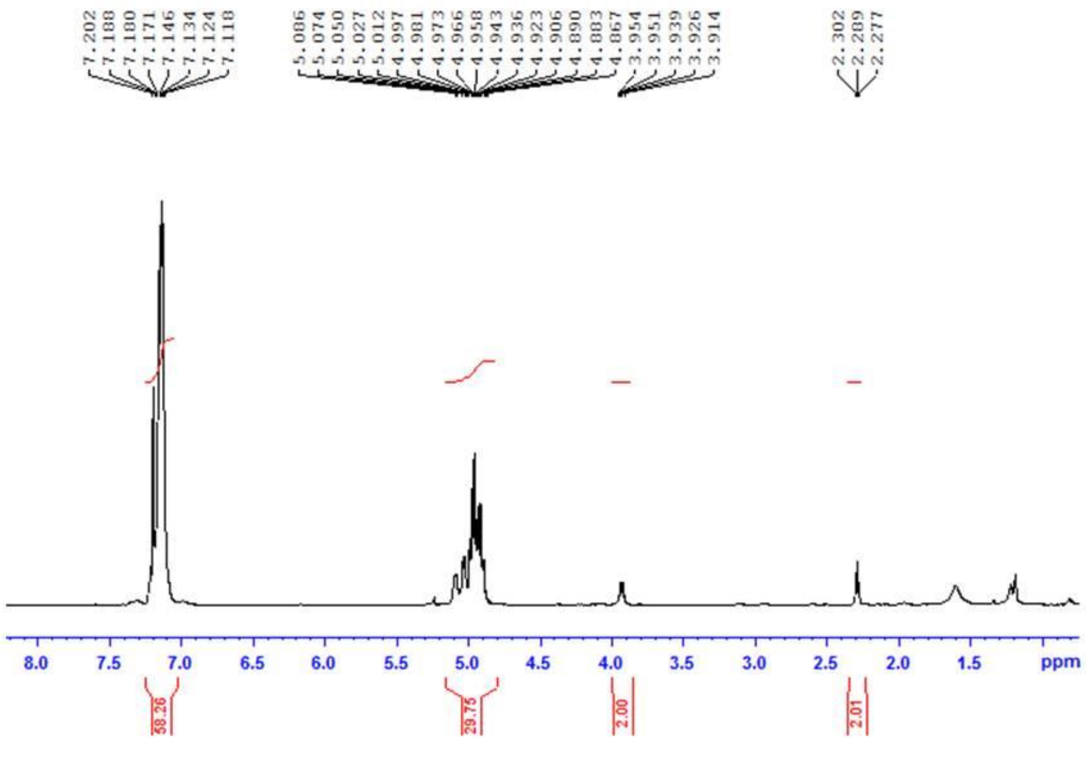

COSY of compound 17

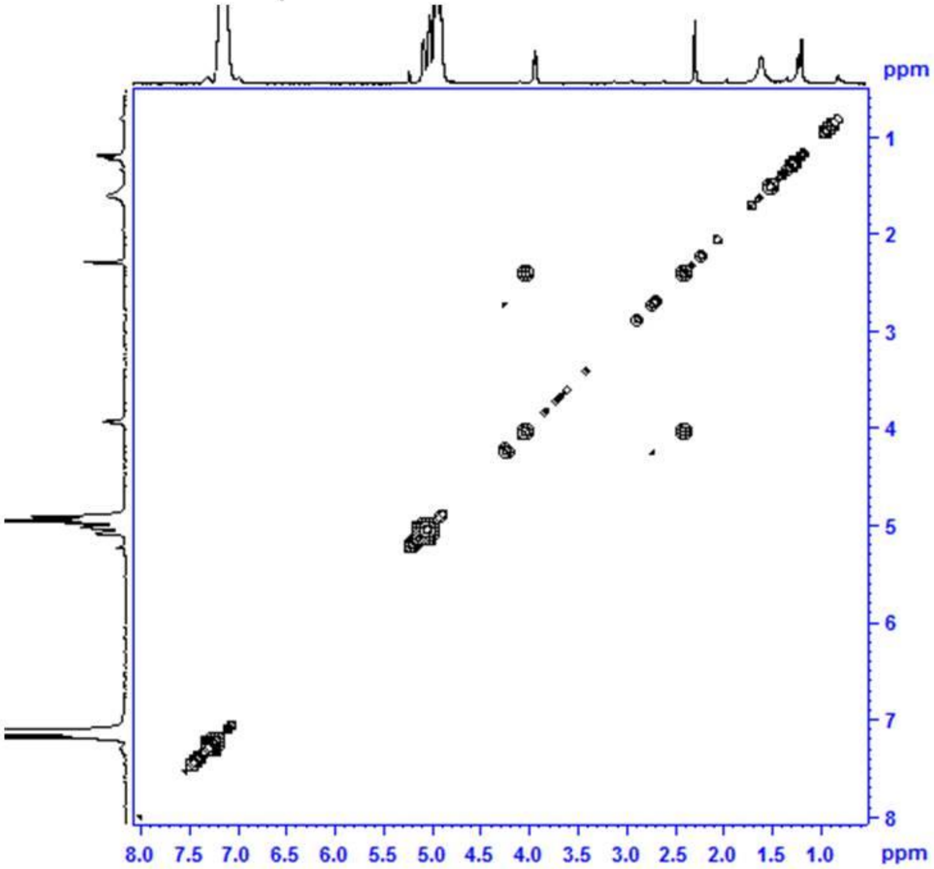

DEPT of compound 17

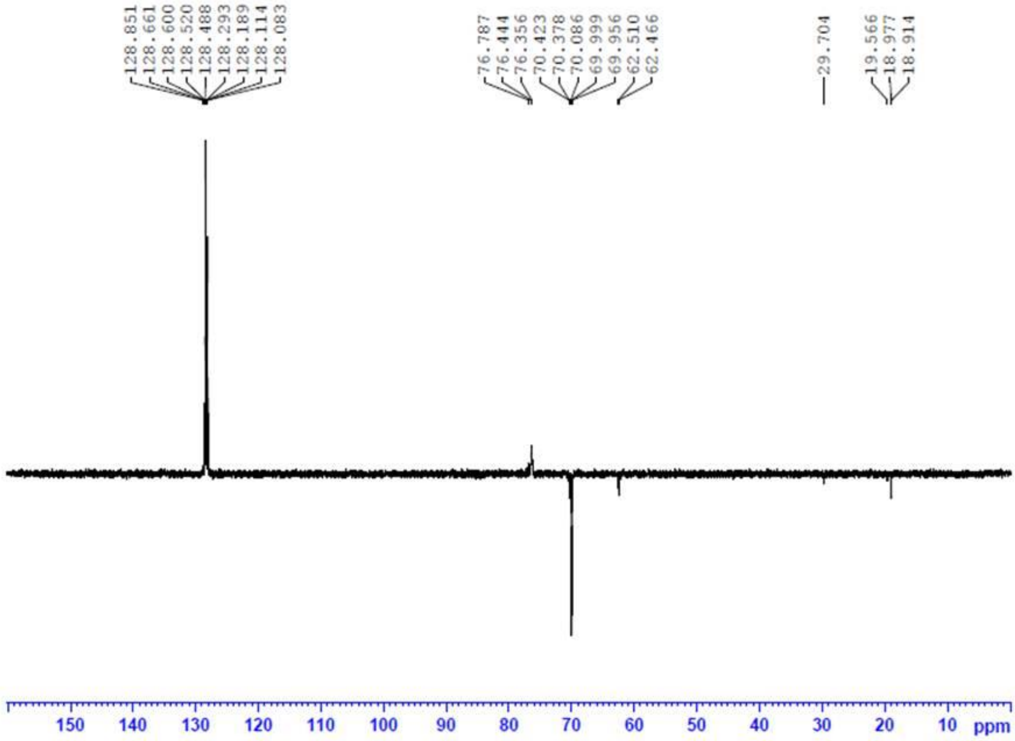

^{13}C of compound 17

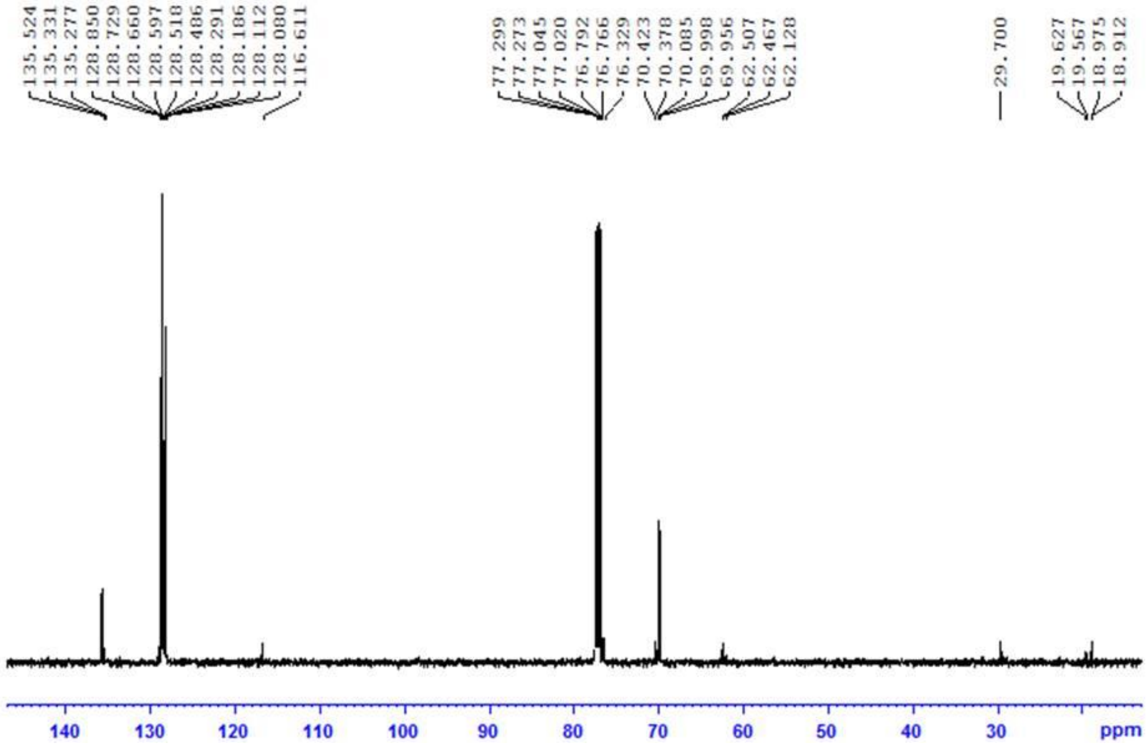

HMQC of compound 17

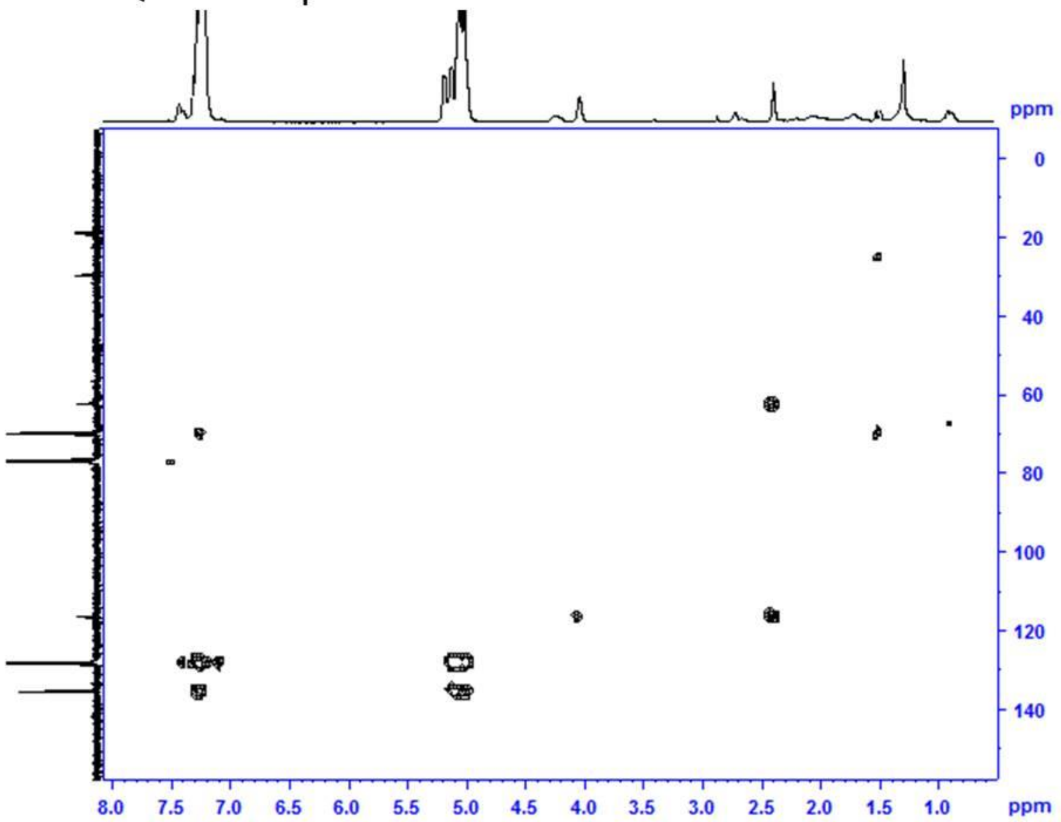

^{31}P of compound 17

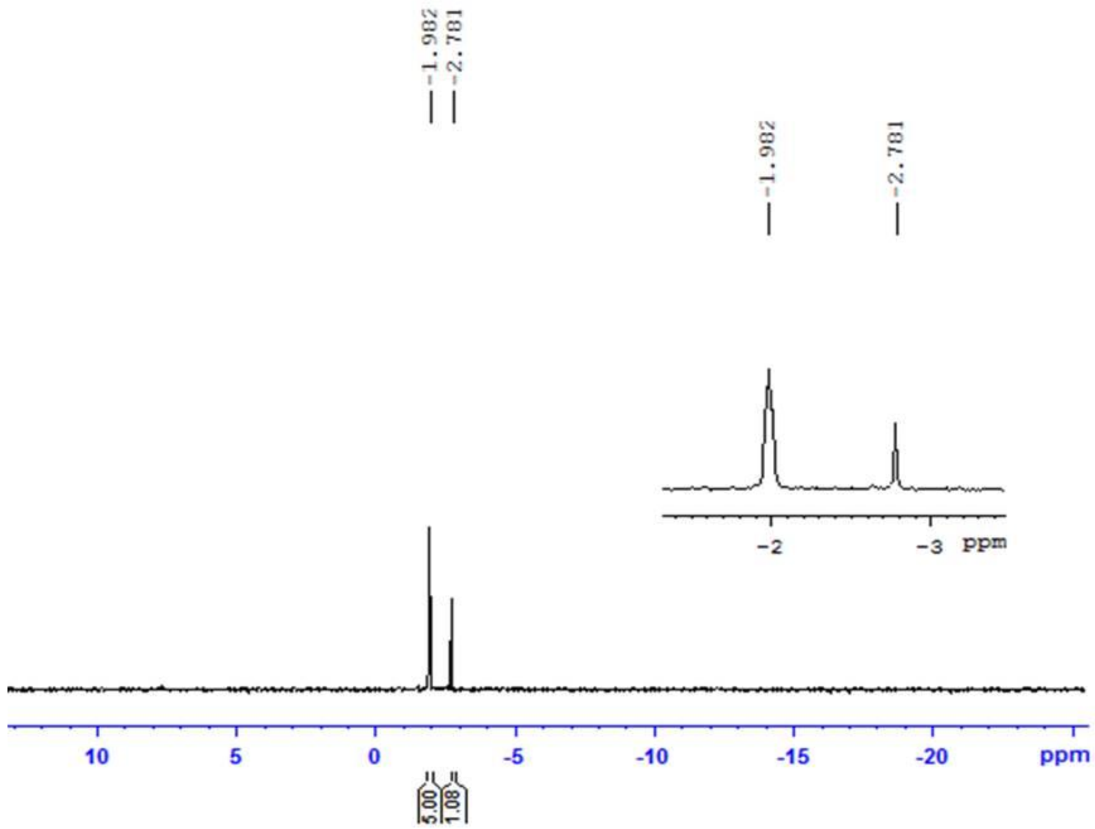

^{1}H of compound 18

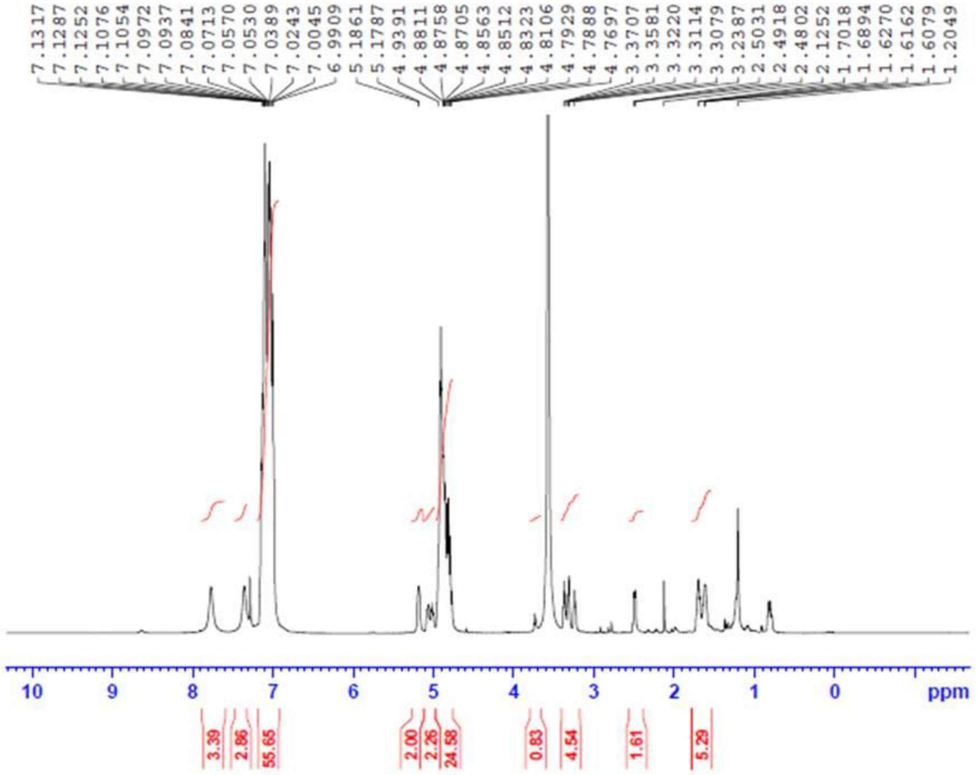

COSY of compound 18

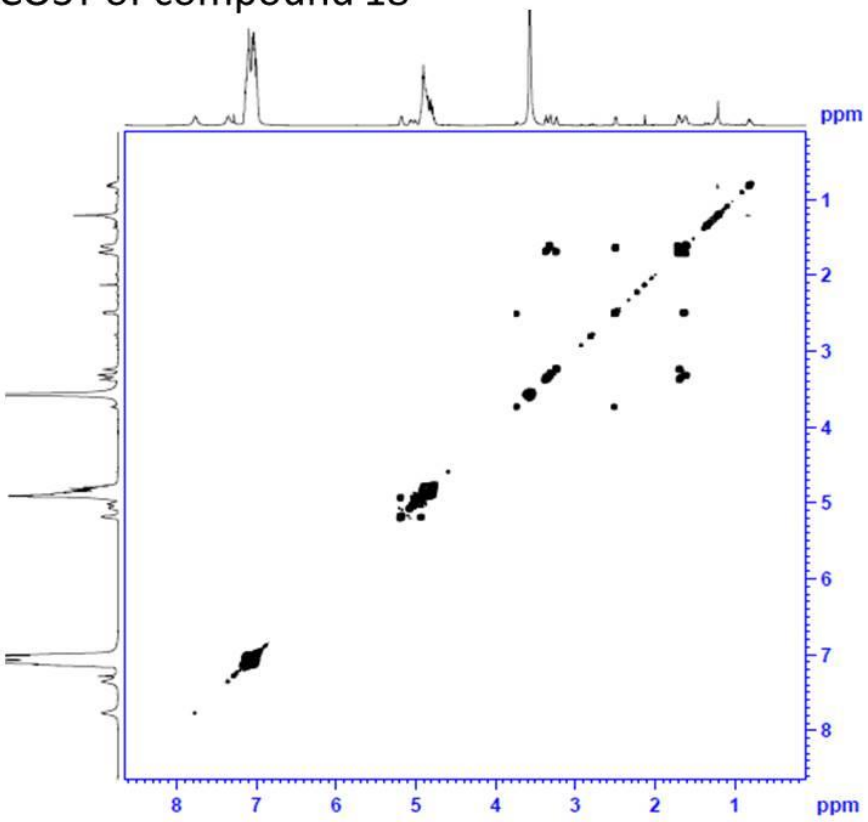

DEPT of compound 18

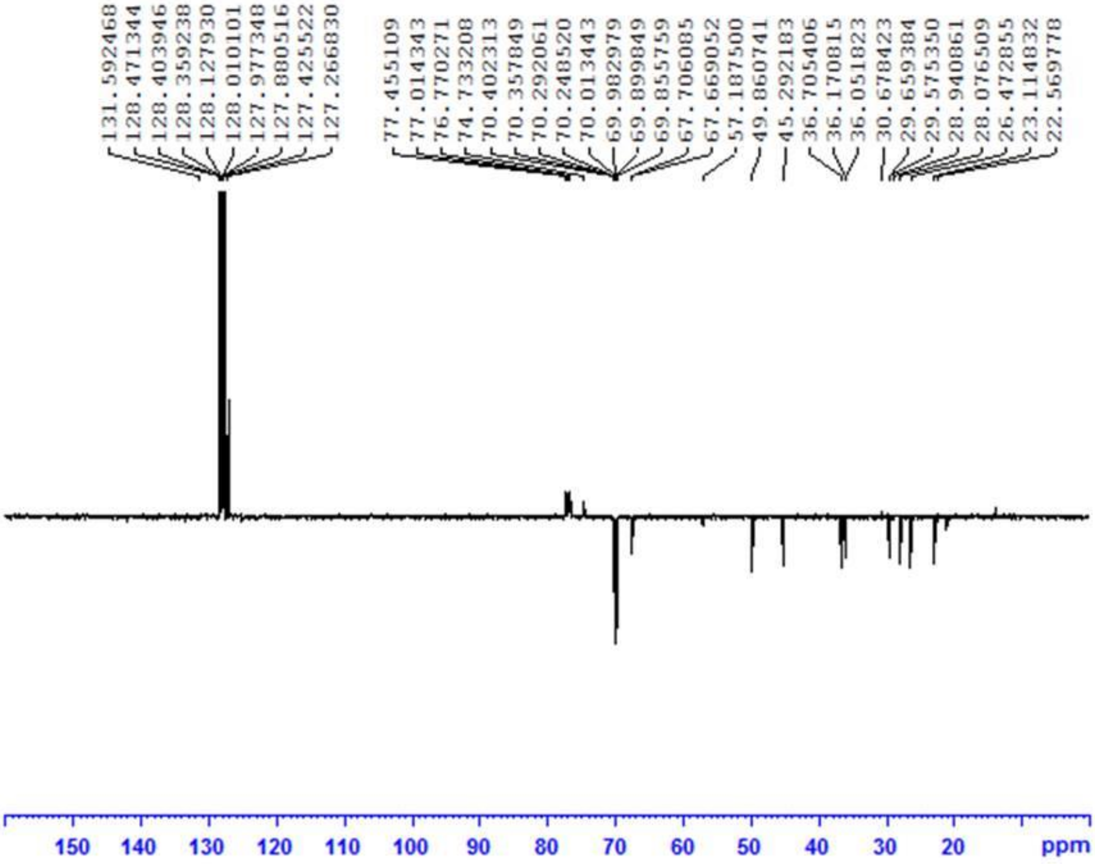

^{13}C of compound 18

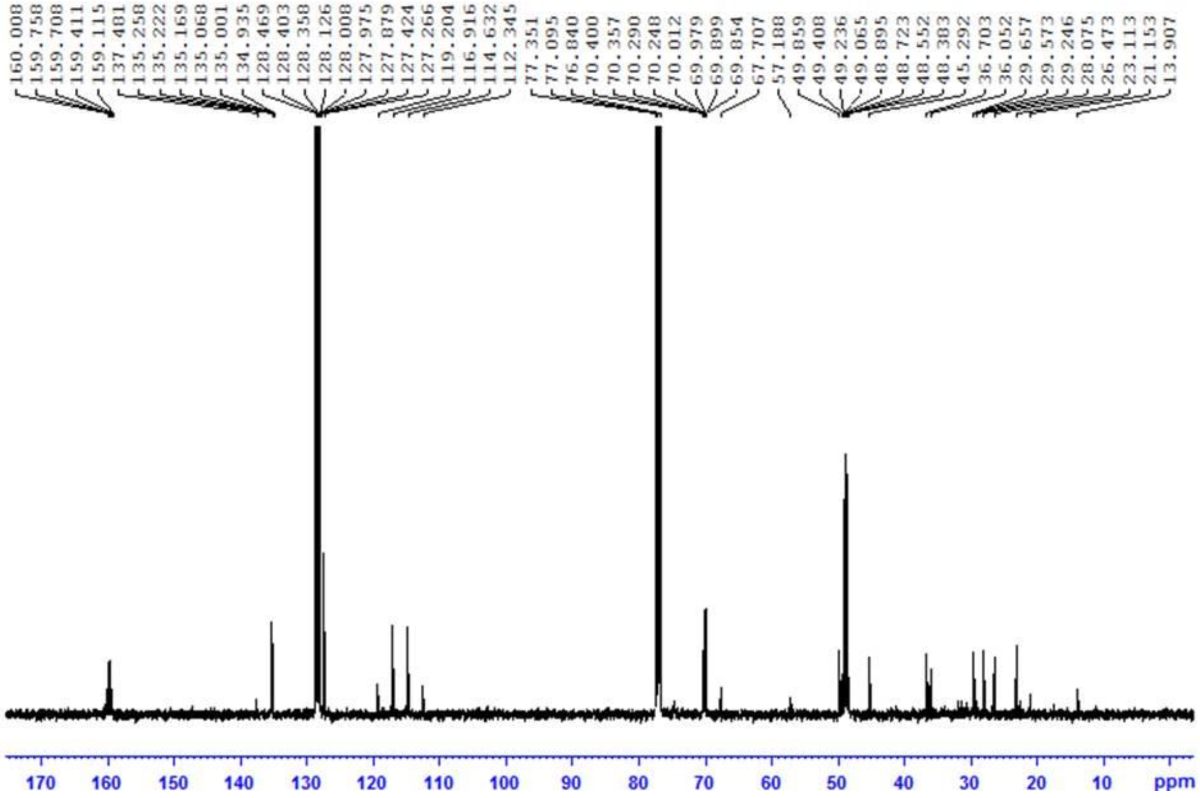

HMQC of compound 18

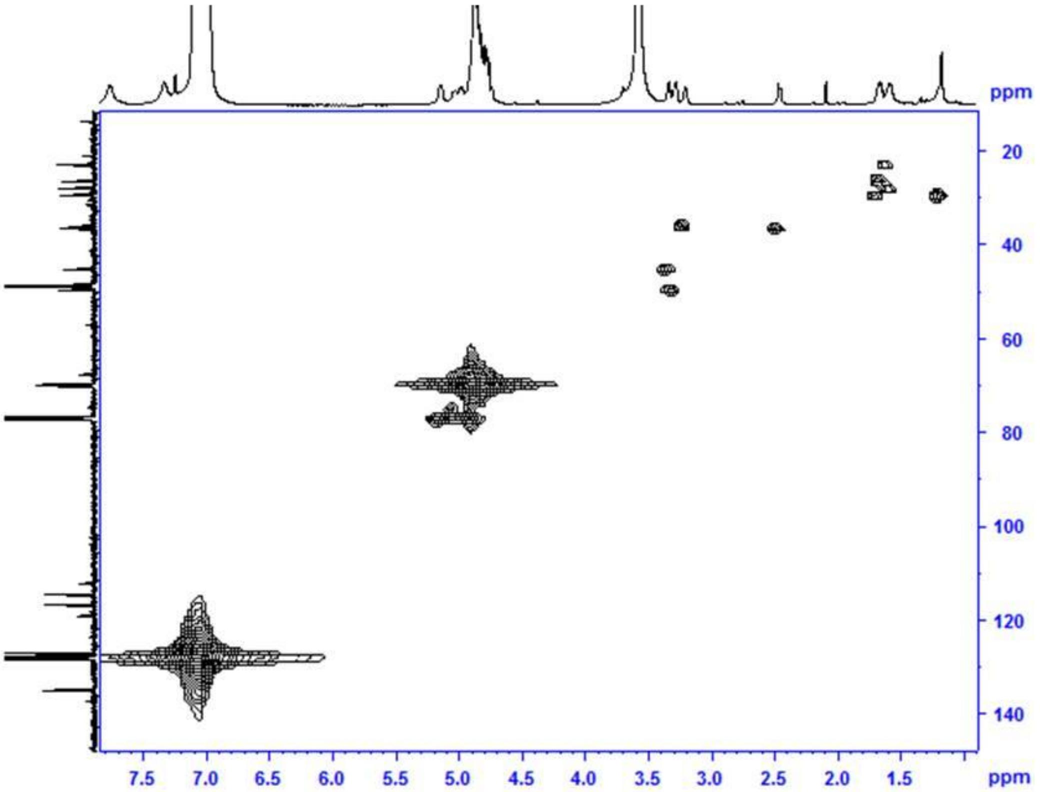

^{31}P of compound 18

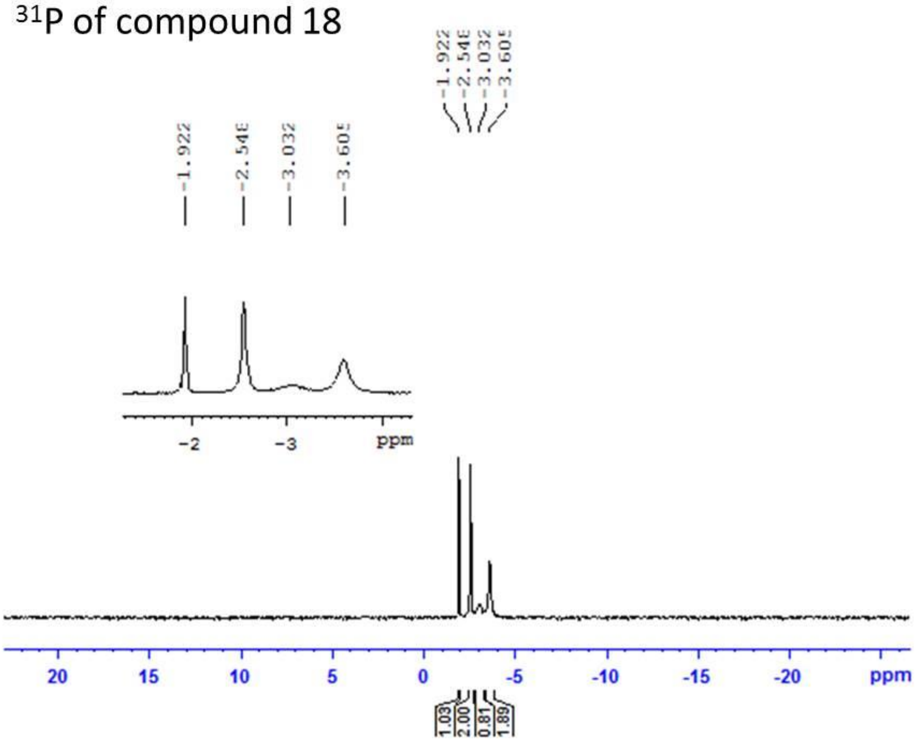

^{1}H of compound 20

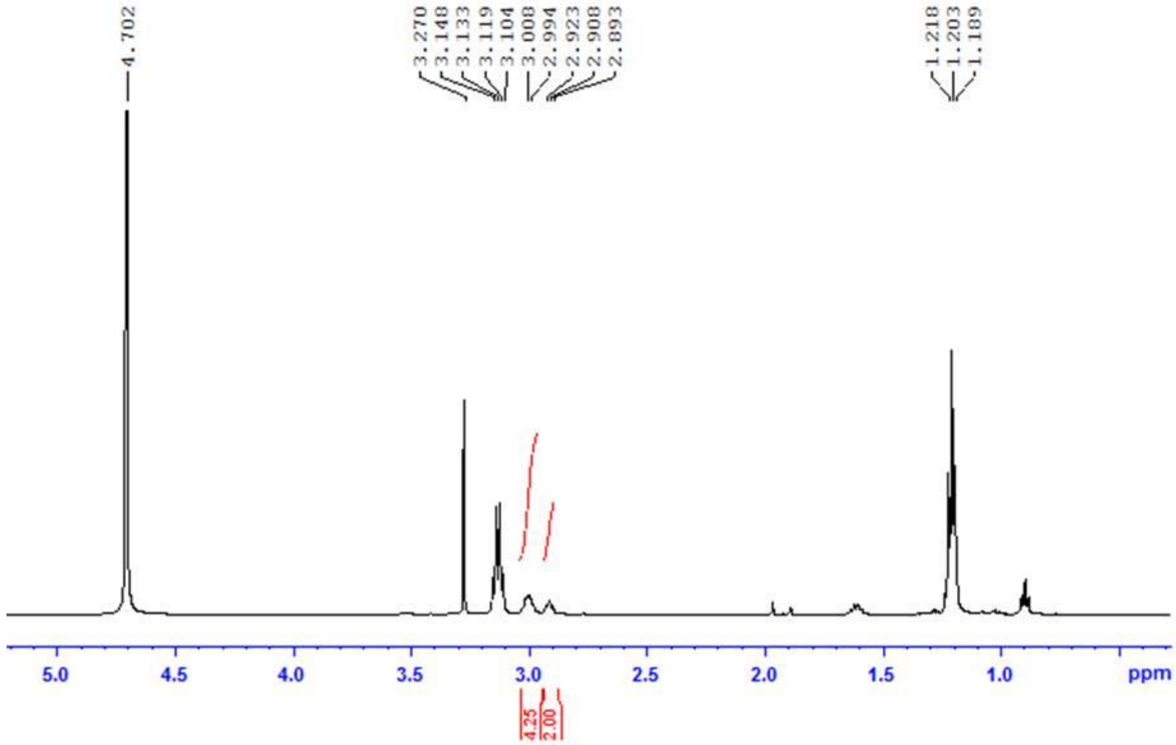

^{31}P of compound 20

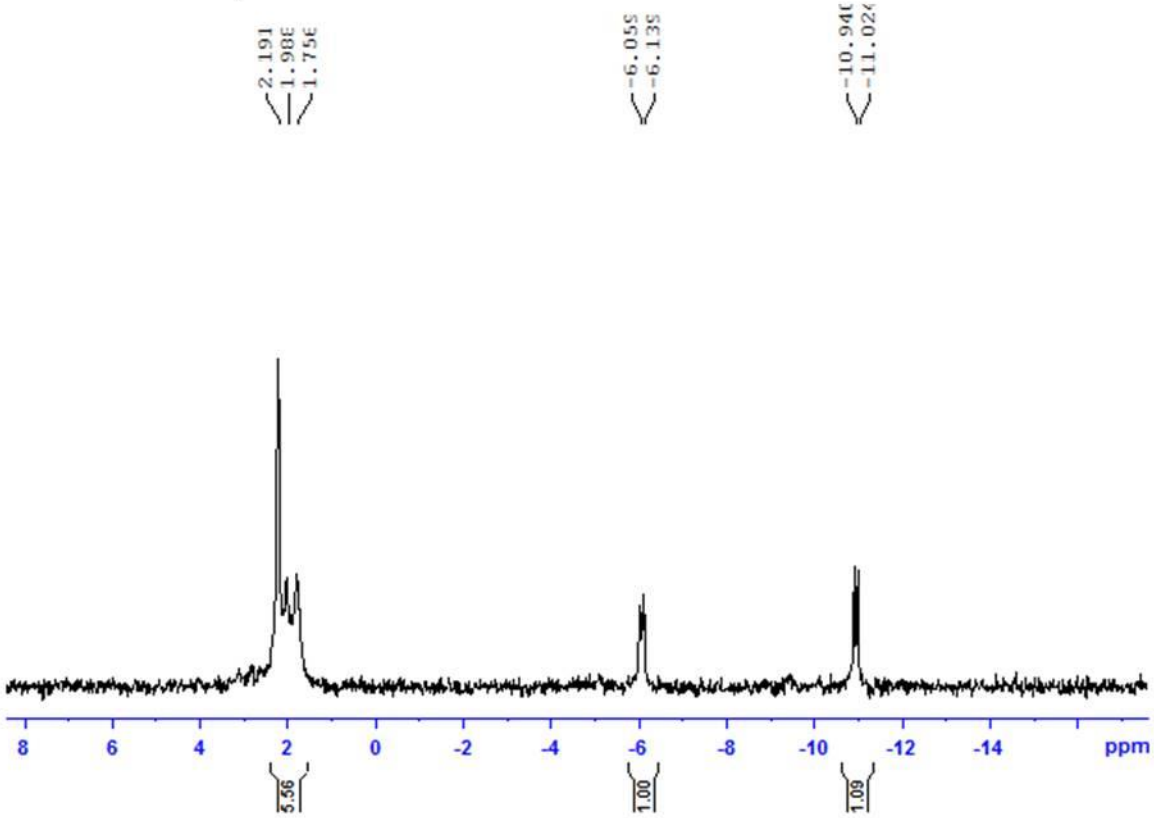

HRMS spectra of compound **20**

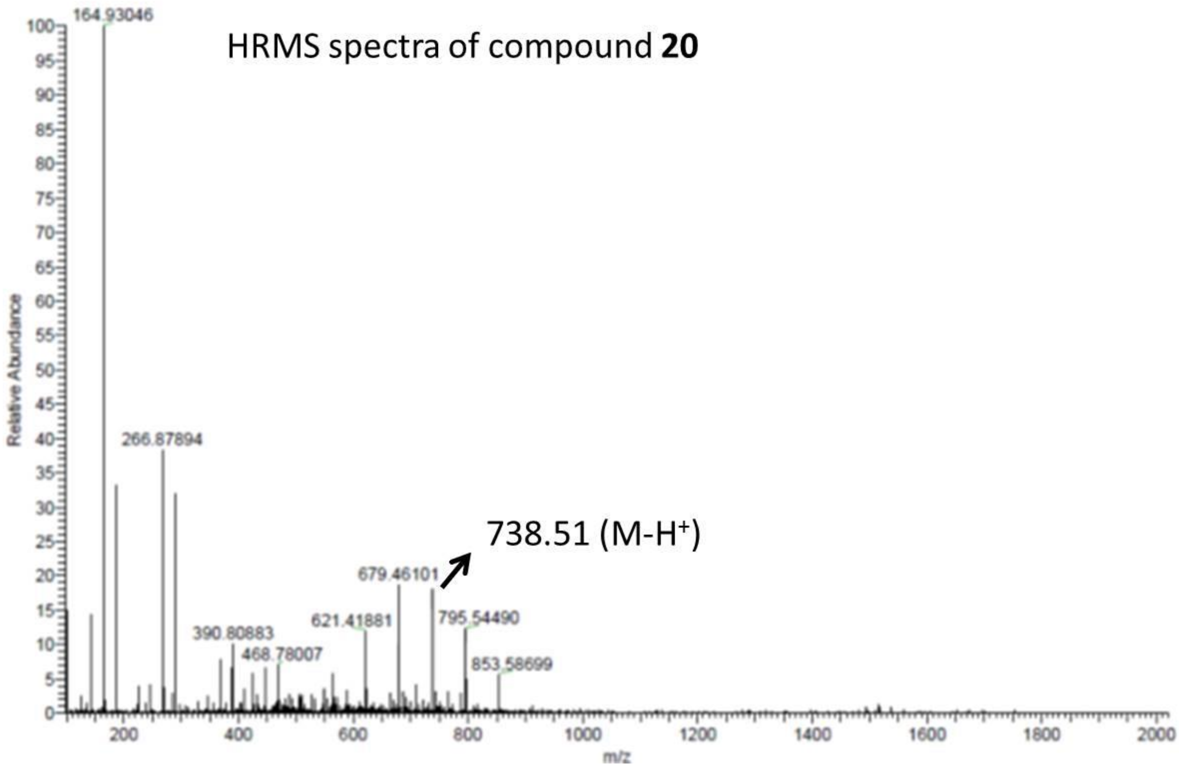

References

1. Losito, O.; Szijgyarto, Z.; Resnick, A.C.; Saiardi, A. Inositol Pyrophosphates and Their Unique Metabolic Complexity: Analysis by Gel Electrophoresis. *PLoS ONE* **2009**, *4*, e5580, doi:10.1371/journal.pone.0005580.
2. Azevedo, C.; Saiardi, A. Extraction and analysis of soluble inositol polyphosphates from yeast. *Nat. Protoc.* **2006**, *1*, 2416–2422, doi:10.1038/nprot.2006.337.
3. Onnebo, S.M.N.; Saiardi, A. Inositol pyrophosphates modulate hydrogen peroxide signalling. *Biochem. J.* **2009**, *423*, 109–118, doi:10.1042/bj20090241.
4. Jones, G.;Willett, P.; Glen, R.C.; Leach, A.R.; Taylor, R. Development and validation of a genetic algorithm for flexible docking. *J. Mol. Biol.*, **1997**, *267*, 727-748, doi.org/10.1006/jmbi.1996.0897.
5. GaussView, Version 6.1, Dennington, R.; Keith, T. A.; Millam, J. M. Semichem Inc., Shawnee Mission, KS, 2016.
6. Avogadro: an open-source molecular builder and visualization tool. Version 1.2.0. Available online: http://avogadro.cc/ (Accessed on 11 June 2021)
7. Riley, A.M.; Unterlass, J.; Konieczny, V.; Taylor, C.W.; Helleday, T.; Potter, B.V.L. A synthetic diphosphoinositol phosphate analogue of inositol trisphosphate. *MedChemComm* **2018**, *9*, 1105–1113, doi:.org/10.1039/c8md00149a.
8. Salentin, S.; Schreiber, S.; Haupt, V.J.; Adasme, M.F.; Schroeder, M. PLIP: fully automated protein–ligand interaction profiler. *Nucleic Acids Res.* **2015**, *43*, W443–W447, doi:10.1093/nar/gkv315.
